# Transcriptome of *Pneumocystis carinii* during Fulminate Infection: Carbohydrate Metabolism and the Concept of a Compatible Parasite

**DOI:** 10.1371/journal.pone.0000423

**Published:** 2007-05-09

**Authors:** Melanie T. Cushion, A. George Smulian, Bradley E. Slaven, Tom Sesterhenn, Jonathan Arnold, Chuck Staben, Aleksey Porollo, Rafal Adamczak, Jarek Meller

**Affiliations:** 1 University of Cincinnati College of Medicine, Department of Internal Medicine, Division of Infectious Diseases, Cincinnati, Ohio, United States of America; 2 University of Georgia, Department of Genetics, Athens, Georgia, United States of America; 3 University of Kentucky, Lexington, Kentucky, United States of America; 4 Division of Biomedical Informatics, Children's Hospital Research Foundation, Cincinnati, Ohio, United States of America; 5 Cincinnati Veterans Administration Medical Center, Cincinnati, Ohio, United States of America; The Research Institute for Children, United States of America

## Abstract

Members of the genus *Pneumocystis* are fungal pathogens that cause pneumonia in a wide variety of mammals with debilitated immune systems. Little is known about their basic biological functions, including life cycle, since no species can be cultured continuously outside the mammalian lung. To better understand the pathological process, about 4500 ESTS derived from sequencing of the poly(A) tail ends of *P. carinii* mRNAs during fulminate infection were annotated and functionally characterized as unassembled reads, and then clustered and reduced to a unigene set with 1042 members. Because of the presence of sequences from other microbial genomes and the rat host, the analysis and compression to a unigene set was necessarily an iterative process. BLASTx analysis of the unassembled reads (UR) vs. the Uni-Prot and TREMBL databases revealed 56% had similarities to existing polypeptides at E values of≤10^−6^, with the remainder lacking any significant homology. The most abundant transcripts in the UR were associated with stress responses, energy production, transcription and translation. Most (70%) of the UR had similarities to proteins from filamentous fungi (e.g., Aspergillus, Neurospora) and existing *P. carinii* gene products. In contrast, similarities to proteins of the yeast-like fungi, *Schizosaccharomyces pombe* and *Saccharomyces cerevisiae*, predominated in the unigene set. Gene Ontology analysis using BLAST2GO revealed *P. carinii* dedicated most of its transcripts to cellular and physiological processes (∼80%), molecular binding and catalytic activities (∼70%), and were primarily derived from cell and organellar compartments (∼80%). KEGG Pathway mapping showed the putative *P. carinii* genes represented most standard metabolic pathways and cellular processes, including the tricarboxylic acid cycle, glycolysis, amino acid biosynthesis, cell cycle and mitochondrial function. Several gene homologs associated with mating, meiosis, and sterol biosynthesis in fungi were identified. Genes encoding the major surface glycoprotein family (MSG), heat shock (HSP70), and proteases (PROT/KEX) were the most abundantly expressed of known *P. carinii* genes. The apparent presence of many metabolic pathways in *P. carinii*, sexual reproduction within the host, and lack of an invasive infection process in the immunologically intact host suggest members of the genus Pneumocystis may be adapted parasites and have a compatible relationship with their mammalian hosts. This study represents the first characterization of the expressed genes of a non-culturable fungal pathogen of mammals during the infective process.

## Introduction

Once thought to be protozoan parasites, members of the genus *Pneumocystis* were placed in the fungal kingdom by phylogenetic analyses of several genes [1]–[5]. The genus Pneumocystis was then placed in the fungal phylum Ascomycota, subphylum Taphrinomycotina (O.E. Eriksson and Winka 1997), Order Pneumocystidales (O.E. Erikss. 1994), Class Pneumocystidomycetes (sensu O.E. Erikss.&Winka 1997), Family Pneumocystidaceae (O.E. Erikss. 1994), Genus Pneumocystis (Delanoë&Delanoë 1912) [6]. The Taphrinomycotina are a paraphyletic group of organisms and the identity of the closest extant relative to the genus Pneumocystis is not yet clear and varies by gene sequences examined and method of comparison. The fungi included within this group are highly diverse and include such members as the fission yeast, *Schizosaccharomyces pombe*, the plant pathogen, *Taphrina deformans*, and *Neolecta vitellina*, the only member with a fruiting body structure [7]. The genus, *Pneumocystis*, is comprised of multiple species that inhabit specific mammalian hosts. To date, 5 species have been formally described [8]. *Pneumocystis jirovecii* infects human beings [9], [10]; *P. murina* is found in mice [11]; *P. oryctolagi* infects rabbits [12] and P. carinii [9], [10]and *P. wakefieldiae* [13], [14] both inhabit the lungs of rats.

These non-filamentous, yeast-like fungal organisms inhabit the lungs of mammals and can cause a lethal pneumonia when the host immune system becomes debilitated or compromised. Infection due to viruses, such as the Human Immunodeficiency Virus (HIV); malnutrition; chemotherapeutic agents; and other underlying diseases can create an environment that permits the growth of *Pneumocystis*. In persons with HIV, pneumonia caused by *Pneumocystis* (PCP) had been a major cause of mortality prior to the advent of Highly Affective Anti-Retroviral Therapy (HAART) [15]. Although treatment with HAART reduced the frequency of infections with *P. jirovecii* and other opportunistic microbes in the United States and Europe, PCP remains an important disease of the immunocompromised. In contrast, there has been a sharp increase in PCP in HIV-infected individuals in underdeveloped and developing countries, such as in sub-Saharan Africa, Asia, and in India where access to HAART is limited or unavailable [16]–[18]. The role *of P. jirovecii* as a potential co-morbidity factor in underlying diseases processes such as chronic obstructive pulmonary disease (COPD) is a focus of several ongoing investigations [19], [20].

Limited therapy is available with which to treat PCP, since these fungi are not susceptible to standard anti-fungal drugs like Amphotericin B or the azole family of compounds. Exacerbating the problem of few alternative chemotherapeutic options is the emergence of mutations in the gene encoding dihydropteroate synthase [21]–[24], the target of the sulfa component of the most efficacious therapy used to treat PCP, trimethoprim-sulfamethoxazole, and in the gene encoding cytochrome b, a target of a secondary therapy, atovaquone [25]. Such mutations in other organisms increased the resistance to these therapies and have been linked to failure of PCP prophylaxis.

*Pneumocystis* maintain an extracellular existence in lung alveoli. Microscopic studies at the light and electron microscopic levels have lead to several proposed life cycles, reviewed [26]. Most include an asexual mode of replication via binary fission of the trophic form and a sexual mode resulting in formation of an ascus (cyst) containing 8 ascospores. Mating is likely mediated by the trophic forms, as evidenced by homologs to yeast pheromone receptor genes present in the *P. carinii* genome [27], [28] and the expression of a pheromone receptor protein on the surface of some trophic forms [28]. Besides the cyst and trophs, there are several intermediate forms that likely represent the progression from zygote through meiosis; the additional mitotic step to produce 8 nuclei; then separation into ascospores. The infection is thought to be initiated by attachment of the trophic forms to the Type I pneumocyte in the host alveoli. However, the mode of travel by the trophic form to the alveoli is unknown, as is the actual infectious propagule. Once in the alveolus, clusters of organisms grow from trophic forms anchored to the Type I cells and fill the lumen. The mode of transmission from one host to another is not known. No environmental form or cycle has been identified. All of the current information on the life cycle has been derived from the study of organisms in the lungs of mammals with debilitated immune systems.

Experimental approaches for the study of these fungi have been limited by the lack of an in vitro culture system. Research has relied on animal models of infection as a source of organisms for biochemical testing, drug evaluation, and microscopic visualization for life cycle analyses. This report describes the transcriptional analysis of *P. carinii* during fulminate infection in the immunosuppressed rat host.

A genome sequencing project was undertaken to probe the complexity of the *Pneumocystis* genome and identify genes that may serve as potential therapeutic targets [29]. The species *P. carinii* was chosen for the project because the immunosuppressed rat provides the highest numbers of organisms that can be obtained reliably from any animal model. Sources of the species found in humans, *P. jirovecii,* are limited and often low in organism numbers. Although similar in some phenotypic traits such as response to therapies and expression of surface glycoprotein variants, the genomes of each species are likely to have unique characteristics. It is a goal of this first *Pneumocystis* genome project to provide a potential genomic scaffold for assembly and comparison of the other members of this genus.

One aim of the project was to create an expressed sequence tag (EST) database from organisms harvested during fulminate pneumonia to identify genes that may be associated with the pathogenic process. Assembly and annotation of the ESTs produced approximately 1,632 gene transcripts. The corresponding cDNA clones were sequenced in the forward and reverse directions to obtain full length gene sequences. In some cases, additional closure was needed to complete the sequences. These sequences were further purged of duplicated genes and sequences from other microbes and host cDNAs to establish a unigene set of 1042 members. Analysis of ESTs and cDNAs showed overwhelming homologies to fungi, further supporting placement of the genus into the fungal kingdom. Functional analyses using the Gene Ontology and KEGG processes showed that *P. carinii* is likely to be capable of a wide variety of metabolic functions but devotes large portions of its transcriptome to the expression of *Pneumocystis-*specific Major Surface Glycoprotein genes (*MSG*) and to energy production during infection.

## Materials and Methods

### cDNA library construction and generation of an EST database

A cDNA library of *Pneumocystis carinii* karyotype form 1 organisms was made from RNA purified using the TriZOL reagent (Invitrogen, Carlsbad, CA) from the lungs of a single, naturally-infected Long Evans rat with a fulminate infection, by construction in the Uni-ZAP XR vector (Stratagene Inc., LaJolla, CA) [27]. The rat was a member of a Pneumocystis-infected rat colony maintained at the Cincinnati Veterinary Medical Unit, Veterans Affairs Medical Center in standard caging racks with access to room air. Fulminate infection in selected members was induced by chronic administration of dexamethasone (4 mg/kg/week) for 10 to 14 weeks as previously described [30]. The primary library consisted of 5×10^5^ clones which was amplified once to a titer of 9×10^11^. The ESTs were sequenced at the University of Georgia sequence center from the 3′ polyA tails (Athens, GA) with Big Dye termination protocols using ABI 3700 instrumentation (Applied Biosystems, Foster City, CA) resulting in about 4500 reads. The average length of a read was about 500 bp.

### Unassembled EST sequences

**Figure 1:**
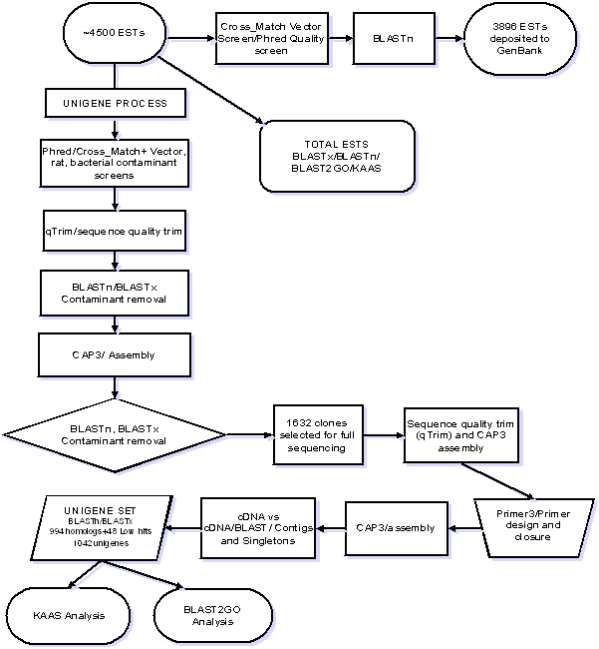
EST Analysis and Unigene Process. The raw sequence reads of the Expressed Sequence Tags were first purged of poor quality sequence (arrows to right of starting point). The resulting 3,896 reads were deposited to NHLBI GenBank and further analyzed for similarities to genes and gene products in the UNI-PROT_TREMBL databases using BLASTn and BLASTx and putative function with BLAST2GO and KASS. The raw reads were then processed using an iterative scheme to form the unigene set starting with a primary screen to purge sequences originating from the cloning vector, rat host and bacteria, followed by a trimming of the sequence ends to reduce poor quality sequence, using an in house program (qTrim). After trimming, the sequences were again purged of contaminants using BLASTn and BLASTx, then assembled using the CAP3 assembly program. After another round of contaminant removal, 1,632 cDNA clones representing putative unique genes were selected for full sequencing. These sequences were then subjected to the same qTrim program, then assembled by CAP3. The primer design program, Primer3 was used to design primers to close gaps in those clone sequence that did not represent full sequences. After another round of assembly, the cDNA sequences were compared to one another for sequence identity using BLASTn, to identify any redundant gene sequences. This resulted in a set of 1042 unique sequences (both contigs and singletons) of which 994 had significant similarities (E≤10^−6^) to genes within the UNI-PROT_TREMBL and 48 did not have significant similarities to existing genes (E≥10^−6^). The unigene set was then analyzed for putative functions by BLAST2GO and KAAS.

The ESTs were processed according to the scheme outlined in Figure 1. In the initial analysis, the sequences were screened for quality, vector and other contaminants (e.g. rat, bacteria) using the Phred and Cross_ Match processing tools (http://www.phrap.org/) [31]–[34] and BLASTn resulting in 3896 ESTs which were submitted to GenBank with ID numbers from AW331850-335745 (Jan. 31, 2000). This initial step was performed in compliance with the Bermuda Principles [35]. The 4500 unassembled ESTs were then analyzed by BLASTx and BLASTn (Oct. 20, 2003) against the UNI-PROT_TREMBL databases (http://www.pir.uniprot.org) to provide an assessment of overall contamination, gene homologies, and relative transcript expression. This set of sequences is referred to as the **“Total ESTs”** throughout the study. The Total EST set was used to populate BLAST2GO and KASS categories at a BLASTx E-value of 10^−6 ^or less required for entry.

### Processing of the unigene set

To arrive at a unigene set, the 4500 sequences were re-processed using Phred and Cross_Match, then trimmed for quality, using an in-house Perl program qTrim available at pgp.cchmc.org, that removed lesser quality flanking sequence that contributed to error rates of 10% or greater [36] (Figure 1). In addition to standard vector screens, several genomes of potential microbes living within the immunosuppressed rat lung were added to the screening library. They included the genomes of *Bacillus subtilis, Pseumodmonas aeruginosa, Pasteurella putida, P. multocida, Staphylococcus aureus,* adenovirus type 12, *Haemophilus influenzae,* mouse adenovirus 1, mouse adenovirusA, and murid herpes virus. The trimmed sequences were then subjected to homology analysis using BLASTn and BLASTx against the NCBI databases (www.ncbi.nlm.nih.gov) (Oct. 2003). *P. carinii* sequences with BLASTn Expect values (E) of less than 10^−100^ to rat, mouse, or bacterial sequences were removed; a BLASTx E value of less than 10^−100^ (i.e. 10^−101^ to complete identity of 0) to rat, mouse or bacterial proteins were also eliminated from the set. The sequences were then assembled using the Cap3 Assembler downloaded from http://www.cs.iastate.edu/∼xqhuang/[37] to reduce redundancy and increase read reliability by condensing overlapping sequence and associated quality scores. Sequences were evaluated for similarity using BLASTx and BLASTn [38] against the NCBI non-redundant database (http://www.ncbi.nlm.nih.gov/Ftp/); UniPROT and UNI-PROT_TREMBL databases (http://www.pir.uniprot.org). Data were analyzed and compiled using Microsoft Excel XP. The UNI-PROT_TREMBL database was chosen for the EST and cDNA sequence analyses due to the extensive degree of annotation and output format.

After manual screening for additional contaminating sequences, 1632 cDNA clones were submitted for sequencing of the full length inserts using the forward and reverse primers of the pBluescript plasmid (T3, T7) to the Cincinnati Childrens Hospital Medical Center Sequencing Core, Cincinnati, OH. The cDNA clones represented what we believed to be non-redundant, non-contaminant sequences. The sequences were evaluated for quality, and trimmed as described above, then assembled with CAP3. In 800 cases, the clone was not fully sequenced and primers were designed to close the gap using Primer3 [39]. The cDNA sequences were screened for redundancy by BLAST analysis of each constituent against the entire cDNA dataset. This resulted in 2 groups within the dataset; one group of contiguous sequences and another comprised of singletons, identified as “cDNAv1_0.fasta.screen.ContigX” and Plate No., Well No.uni.t/f.ab1 (e.g. 14e02.uni.t.ab1), respectively. Both groups were evaluated for homology to other gene sequences using BLASTx and BLASTn against the databases from NCBI (nr), Swiss Prot, and TREMBL (May 2004). This resulted in removal of additional rat/mouse and bacterial sequences (E≤10^−40^) and resulted in 981 sequences in the unigene collection that had homology to proteins in the databases, including hypothetical proteins. An additional 74 had E-values greater than 10^−6^ . Within the 981 sequences were 45 contigs with similarities to mammalian genes (E values≥10^−40^). During the revision of this manuscript, the 74 “low score” hits and the putative mammalian conserved genes were re-analyzed. Twenty-six of the low score contigs were found to have similarities to proteins in the database with E values≤10^−6^ resulting in a shift of 26 contigs to the 981 sequences with significant identities to gene homologs. The remaining 48 sequences with E values of 10^−6^ or greater were retained in the unigene set as well. The 45 sequences with BLASTX E-values of≥10^−40^ to mammalian gene homologs were also re-analyzed to assess whether these were host in origin, or if they were indeed conserved genes in the *P. carinii* genome. The sequences were analyzed for total AT content, then re-assessed in relation to their BLASTx and BLASTn E-values. The *P. carinii* genome has a high AT content (∼68%) vs. the rat genome (∼50%). There was a clear demarcation between sequences in the 45 contig set based on AT content. Thirteen contigs were characterized as mammalian genes and eliminated from the unigene set. The 13 eliminated sequences had an average AT content of 52.6 while the retained sequences had an AT content average of 68.8. The remaining 33 sequences all had significant similarities (E values of≤10-6) to fungal or other protistan proteins and were included in the subsequent unigene homolog analyses. Within the unigene set were 994 sequences with significant similarities to proteins within the databases queried plus 48 sequences that did not have any significant similarities for a total of 1042 unigenes. This set is referred to as the “Unigene set” throughout the study.

### Analysis of function

The 1042 unigenes and the ∼4500 ESTs were submitted for Gene Ontology (GO) annotation to the online version of the BLAST2GO v1 program (www.Blast2GO.de) [40] (November 2006) . The program extracts the GO terms associated with homologies identified with NCBI's QBLAST and returns a list of GO annotations represented as hierarchical categories of increasing specificity. BLAST2GO allows the selection of a significance level for the False Discovery Rate (FDR) which was used as a cut-off at a 0.05% probability level. The data presented herein represent the level 2 analysis, illustrating general functional categories.

Placement into metabolic pathways was accomplished with the tools supplied by the Kyoto Encyclopedia of Genes and Genomes (KEGG) (June, 2006), located at the KEGG Automatic Annotation Server (KAAS), http://www.genome.jp/kegg/kaas/. The EST reads and cDNAs were processed using the bi-directional best hit method (forward and reverse reads) to assign orthologs. KAAS provides functional annotation of putative genes by BLAST comparisons against the KEGG GENES database. The output includes KO (KEGG Orthology) assignments and automatically generated KEGG pathways that are populated with the KO assignments. The sequences were submitted for analysis using all available databases and to those databases that only included fungal genomes. In some cases, manual annotation, literature searches and the yeast website, http://www.yeastgenome.org were used to supplement pathway details.

### Transcript abundance

The unassembled and trimmed ESTs were aligned with the unigene set prior to purging of redundant genes using the BLAST algorithm to identify those genes with the highest transcription abundance [38]. The cutoff for identity of the aligned ESTs to each unigene was set at E≤10^−50^.

## Results

### Transcript abundance

Members of the genus Pneumocystis conduct their extra cellular life cycle in the presence of a mammalian immune system and in an environment provided by the host lung which includes many factors such as surfactant proteins, lipids, and extra cellular matrices. Analysis of the 17 most abundant transcripts used by the organisms in the context of this milieu revealed a striking pattern (Table 1.). The majority of the transcripts were related to stress responses (7/17). Other abundant transcripts included gene homologs associated with aerobic respiration (2/17), transcription and translation (6/17), and sporogenesis and mating (2/17). The stress responses may have been induced by nutritional limitation or other adverse factors within the lung alveoli induced by late stage infection; by oxidative stress initiated by the host immune system; or due to the isolation process that was used to separate the organisms from the host tissue. Of interest was the expression of genes involved in sporogenesis and mating (*STE11, CON7*). Many fungi initiate sexual reproduction resulting in spores as a result of stress stimuli or nutritional limitation. The co-expression of the stress-related gene homologs and those involved in sporogenesis may indicate that sexual replication in *P. carinii* may also be induced by such factors.

**Table 1:** Transcript abundance.

Unigene identifier	No. ESTs	Gene homolog	Organism	Metabolic role
Contig1035	281	HSP70	*Pneumocystis carinii*	Stress related
Contig1031/147*	148	STE11	*Pneumocystis carinii*	Mating; nutritional stress
Contig1055	140	SFP1	*Saccharomyces cerevisiae*	Stress related; nutritional limitation
Contig1034/946*	138	COX II precursor	*Saccharomyces cerevisiae*	Aerobic respiration
Contig1039	89	HSP90	*Schizosaccharomyces pombe*	Stress-related
Contig498	84	MSG**	*Pneumocystis carinii*	Variable surface antigen
Contig520	84	CON7	*Magnaporthe grisea*	Sporogenesis
Contig1056	81	PDR3	*Saccharomyces cerevisiae*	Transcriptional activator; oxidative stress
11d08	74	Q9AVH2	*Pisum sativum*	Senescence-associated; dehydration
Contig909	69	SIS1	*Yarrowia lipolytica*I	Stress related
Contig1037/287*	68	MLO3	*Schizosaccharomyces pombe*	mRNA export
Contig176	66	Mitochondrial genome§	*Saccharomyces cerevisiae*	Aerobic respiration
Contig1016	60	PSI1	*Schizosaccharomyces pombe*	Translation
Contig799	38	BBP	*Schizosaccharomyces pombe*	Intron splicing
Contig8	24	SIN1	*Schizosaccharomyces pombe*	Oxidative stress related; mitosis
Contig298	22	EF1A	*Candida albicans*	Protein synthesis
Contig 947	21	TFIID	*Pneumocystis carinii*	Transcription

* Contig purged from final unigene set due to redundancy

** Variant 2 major surface glycoprotein isotype

§ Low homology

### Analysis of unassembled EST sequences

**Figure 2:**
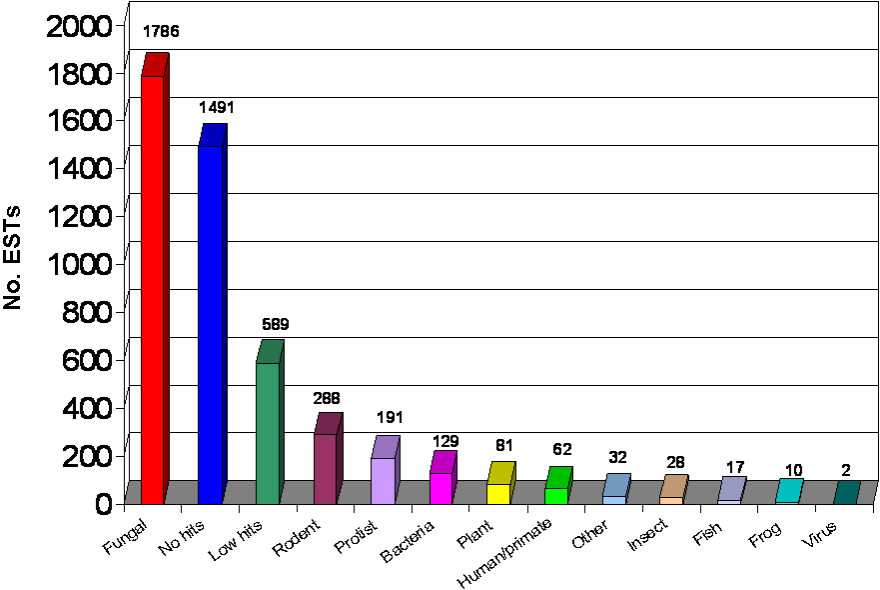
Binning of *P. carinii* EST gene homologs by general organism groups. After trimming the raw EST reads for poor quality sequence (qTrim), the ESTs were analyzed for similarities to gene products of other organisms using BLASTx. Significance was set at E≤10^−6^.

EST sequence homologies with E values of 10^−6^ or less after BLASTx analysis were sorted into 13 general categories shown in Figure 2. Most of the sequences shared similarities with known genes (56%) while 44% showed little to no similarities with genes in the UniProt_TREMBL database. Of those *P. carinii* sequences with shared protein similarities, the greatest number fell within the fungal category (44%). Bacterial contaminants, either from the rat lung or from the *E. coli* used to produce the plasmids for sequencing, accounted for less than 3% of the reads. There was very little viral contamination, but 7% of the sequences were identified as homologs to rodent genes, from either mouse or rat. These were ultimately purged from the unigene collection. A small percentage of the *P. carinii* sequences (5%) were similar to other protistan gene products, such as the Cryptosporidiidae, *Dictyostelium discoideum,* and trypanosomes. Plant genes were also represented with about 2% of the *P. carinii* ESTs showing similarity to a putative senescence-associated protein in the pea.

**Figure 3:**
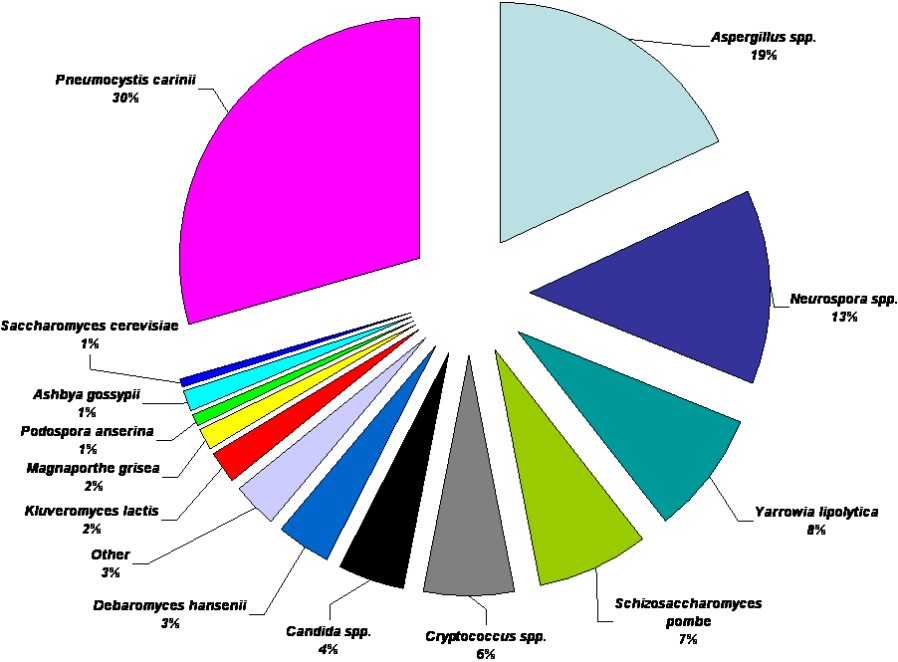
Binning of putative fungal gene homologs of *P. carinii* ESTs. EST sequences with significant similarities to fungal genes with BLASTx scores of E≤10^−6^ were binned according to fungal species.

Within those transcripts with similarities to fungal proteins, the majority identified previously known *Pneumocystis* proteins (30%) (Fig. 3). Outside of its own genus, sequences had the highest similarities to gene products of the filamentous fungal *Aspergillus* species (19%) and *Neurospora crassa* (13%). Similarities to yeast-like fungal proteins included the ascomycete, *Yarrowia lipolytica* (8%)(12% if combined with the Candida spp.)*;* the fission yeast, *Schizosaccharomyces pombe* (7%); and the *Saccharomyces* species (1%). The remaining homologs were spread over a wide variety of fungal species.

**Figure 4:**
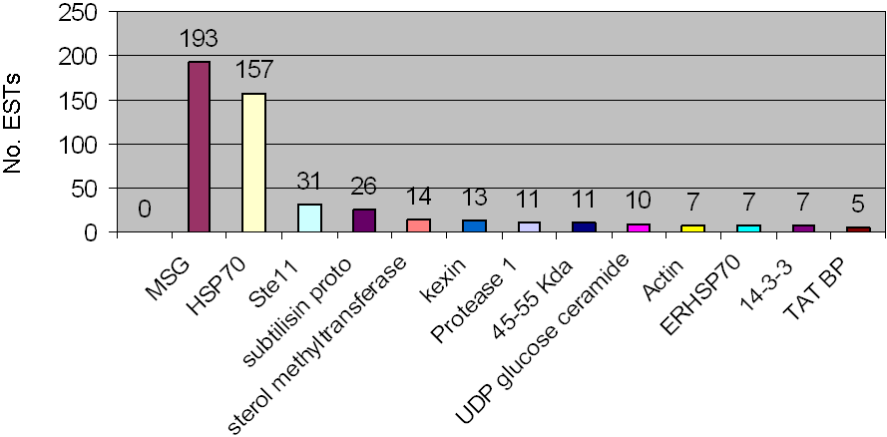
Distribution of *P. carinii* ESTs with significant similarities to known Pneumocystis genes. EST sequences with significant similarities to known *P. carinii* genes with BLASTx scores of E≤10^−6^

The distribution of ESTs with similarities to known *P. carinii* genes is shown in Figure 4. Only those genes with more than one EST are shown. The repetitive gene family of major surface glycoproteins (*MSG*) and the MSG-related genes, *MSR*, constituted almost 40% of the 526 expressed *P. carinii* genes. Another repetitive gene family, collectively called the kexin or protease family, accounted for about 10% of the ESTs. Heat Shock protein 70 genes and endoplasmic reticulum *HSP70* homolog precursors represented 30% of the 526 sequences. Putative single copy genes of *P. carinii* that were represented by several ESTs included the HMG box protein *STE11*, Actin, the 45-55 Kda antigen, a TATA-binding protein, proteins with the 14-3-3 motif, UDP-glucose: ceramide glucosyltransferase (cerebroside synthase), an *ERG6* homolog (sterol methyltransferase) and a potential drug target, inosine monophosphate dehydrogenase (*IMPDH*).

### Gene ontology analysis, unassembled ESTs

The B2G program uses BLAST to find homologous sequences to input sequences and extracts GO terms (Gene Ontology) to each obtained hit using existing annotations. These GO terms are assigned to the query sequence resulting in an assessment of the biological process, the molecular function and the cellular compartments represented. In this case, the GO assignments would provide a basic assessment of the more important organism processes occurring in severe infection. Most of the ESTs did not have GO assignments (∼2250) (Figure S1). This is likely due to the overabundance of *P. carinii* genes identified, since few have associated GO terms and to the 44% of the ESTs lacking significant similarity to any known protein. Of those sequences with GO assignments, many had 3–5 assignments each and a significant number (about 50) had 9 or more assignments. Despite these limitations, the present report provides a preliminary, yet informative picture of the metabolic processes that are likely to be operational during infection.

### Representation of the unassembled ESTs in the GO categories

**Figure 5:**
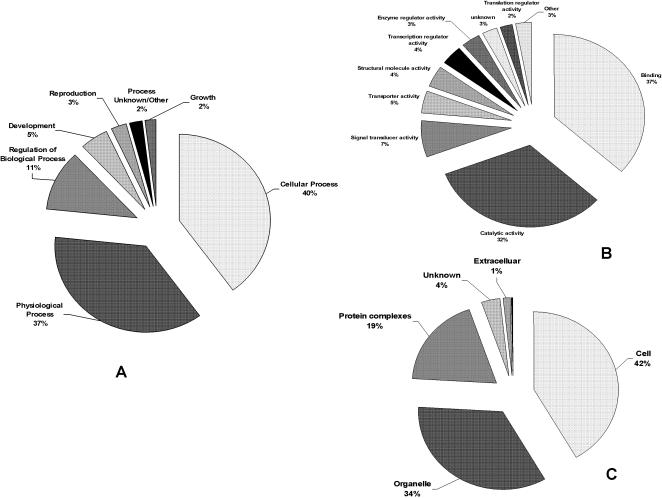
BLAST2GO categories of ESTs. ESTs were analyzed using the BLAST2GO software. Shown are level 2 categories for Biological Processes (A); Molecular Function (B); and Cellular Components (C).

The majority of the ESTs (almost 80%) were dedicated to cellular and physiological processes in the biological process category, with an additional 11% assigned to regulation of the biological processes (Figure 5A). The biological process category refers to a biological objective to which a gene contributes, but does not identify pathways. Examples of such processes include metabolism, cell communication, and sexual reproduction. Level 2 biological processes are shown throughout this report. A more fully detailed hierarchical breakdown for this and the other 2 GO categories can be found in the Tables S1, S2, and S3.

Molecular function in the GO resource is defined as “what a gene product does at the biochemical level”. This is a very narrow definition as it does not take into consideration the location of the event or the function in a broader, pathway or network context. In this category, most of the ESTs (∼70%) were dedicated to binding and catalytic functions (Fig. 5B). Most of the binding functions were at the intracellular level rather than external and included nucleotide and nucleic acid binding, protein binding and ion binding. Catalytic activities included transferase and hydrolase activities.

The final category identifies the locations in the cell where the gene products are found. These range from a general placement, such as in the “cell membrane” to more specific, such as the “histone deacteylase complex”. The *P. carinii* gene products were found generally associated with the cellular components, in the intracellular space (42%) or in organelles, (34%), of which more were associated with membrane bound organelles such as the mitochondrion (Fig. 5C). Almost 20% were found in protein complexes, such as with the ribonucleoprotein complex.

### Analysis of the Unigene set

**Figure 6:**
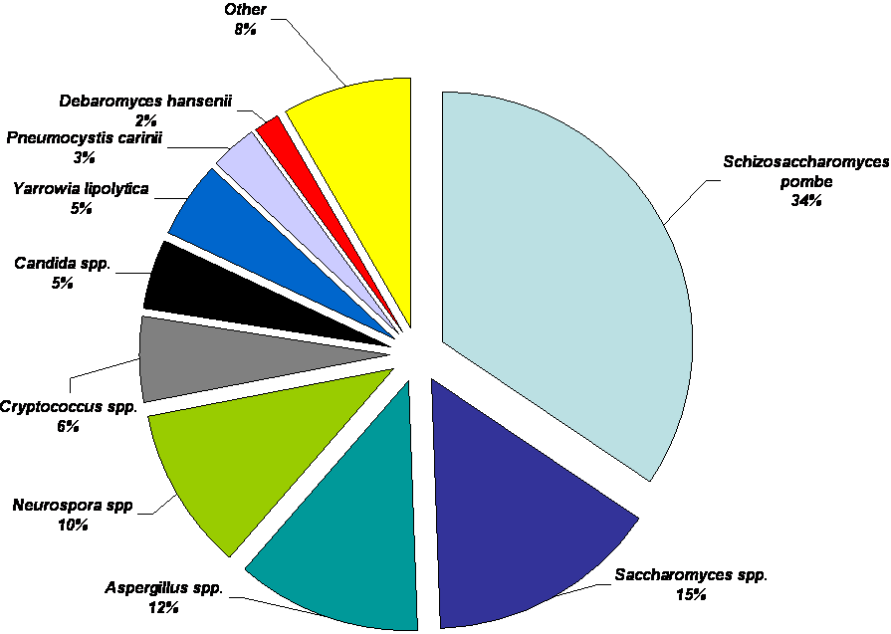
Binning of putative fungal gene homologs of *P. carinii* Unigenes. The 918 unigene sequences with significant similarities fungal genes (BLASTx of E≤10^−6^ ) were binned according to fungal organism of origin.

A total of 1042 non-redundant sequences were included in the unigene set after processing (Figure 1.). The unigenes were categorized first by gene homolog organism of origin (Figure S2). The overwhelming majority of the sequences were homologs of fungal genes (93%). In contrast to the EST data set, the unigenes had the highest similarities to proteins of yeast-like fungi (Fig. 6), including *S. pombe* (34%) and *S. cerevisiae* (15%) rather than the filamentous fungi, *Aspergillus* (12%) or *Neurospora* (10%). This apparent discrepancy was due to the overabundance of certain transcripts that were similar to a few proteins from filamentous fungi in the EST database, whereas these were collapsed into single genes in the unigene set.

### GO analysis of Unigene set

Functional assignments by the GO analyses of the unigene set were almost identical to the results from the ESTs, as expected. These graphs can be viewed in Figure S3A–C.

### KEGG analysis of the unigene set

Approximately 39% of the unigenes were assigned to KEGG pathways using the KEGG Automatic Annotation Server (KAAS) with all organism pathway databases included. When only the fungal databases were selected, slightly fewer sequences were used to populate the fungal pathways, perhaps due to *P. carinii* genes that have conserved function to genes within genomes other than fungi. The remaining 60% were not assigned due to lack of EC numbers in the initial BLAST analysis; the presence of *P. carinii*- specific genes without apparent pathway classifications, such as the *MSG*s; or the lack of homology to known pathway genes. Regardless, the pathways represented by the *P. carinii* transcripts provide us with the first insights of the metabolism of these fungal organisms residing in the host lung. Shown in Table 2. is the distribution of the unigenes into general metabolic pathways used by KEGG. Table S4. shows a detailed breakdown of the unigene locations within each pathway included within the general categories. Table S5 identifies each of the unigenes that entered into each pathway. The unigene identifiers can be used to retrieve the DNA sequences from the Pneumocystis Genome Project website: http://pgp.cchmc.org

**Table 2:** KEGG biochemical pathway mappings for *P. carinii* unigenes.

KEGG Category	Enzymes represented
01110 Carbohydrate Metabolism	48
01320 Signal Transduction	42
01420 Cell Growth and Death	36
01150 Amino Acid Metabolism	33
01120 Energy Metabolism	25
01220 Translation	18
01190 Metabolism of Cofactors and Vitamins	18
01430 Cell Communication	19
01230 Folding, Sorting and Degradation	17
01460 Immune System	16
01130 Lipid Metabolism	14
01140 Nucleotide Metabolism	14
01210 Transcription	14
01170 Glycan Biosynthesis and Metabolism	10
01410 Cell Motility	8
01195 Biosynthesis of Secondary Metabolites	6
01450Behavior	6
01510 Neurodegenerative Disorders	6
01196 Xenobiotics Biodegradation and Metabolism	5
01470 Nervous System	5
01160 Metabolism of Other Amino Acids	4
01440 Development	4
01530 Metabolic Disorders	2
01240 Replication and Repair	1
01330 Signaling Molecules and Interaction	1

The pathways with most representation by the unigenes were carbohydrate metabolism (47 members); signal transduction (42 members); cell growth and death (36 members); and amino acid metabolism (33 members) (Table 2.).

The gluconeogenesis/glycolysis pathways held the largest number of unigenes within the carbohydrate metabolism category. All yeast use sugars as carbon sources from which they convert glucose-6-phosphate or fructose-6-phosphate to pyruvate via glycolysis. The production of energy in the form of ATP in the 10 enzyme-catalyzed process of glycolysis is linked to generation of intermediates and reducing power in the form of NADH which are subsequently used in biosynthetic pathways. Under aerobic conditions, oxidation of pyruvate to carbon dioxide will predominate, whereas under anaerobic conditions, transformation to ethanol prevails. The fate of pyruvate in aerobic processes depends on the energy charge of the cell. In cells with a high energy charge, pyruvate is driven toward gluconeogenesis, when it is low, pyruvate is preferentially oxidized to CO_2_ and H_2_O in the TCA cycle.

### Glycolysis

Homologs to 8 of the 10 enzymes in the glycolytic pathway were identified in the unigene set or as genomic copies (Table 3.), suggesting this pathway is operational during infection in the mammalian lung. Of note, was the possession of only a single hexokinase gene homolog by *P. carinii* which was verified at the genome level. *Candida tropicalis, Schizosaccharomyces pombe* and *Saccharomyces cerevisiae* possess 2 hexokinases within their genomes, *HXK1* and *HXH2*, while *Kluveromyces lactis,* and *Yarrowia lipolytica* genomes contain only a single hexokinase gene. In contrast, like most other fungi, the *P. carinii* genome contained 2 homologs of phosphofructokinase , *PFK1* and *PKF2.* The energy rich acylphosphate derived by the action of 3-phosphoglycerate kinase on 1,3-bisphosphoglycerate, is used to produce ATP. In yeast, the *PGK1* gene encoding this protein is one of the mostly highly expressed. The gene homolog was represented in the *P. carinii* ESTs and as a contig in the unigene set. Homologs to the proteins directing the last 3 reactions of glyclolysis were all identified in the genome and two of three were expressed as transcripts (Table 3.). Like *S. cerevisiae* and *C. albicans, P. carinii* possesses 2 enolase gene homologs, *ENO1* and *ENO2*, encoding subunits of the homodimeric protein [41]. *S. pombe* also appears to contain 2 enolase genes, the second of which was identified as a result of its genome project (http://www.genedb.org/genedb/)/. Pyruvate kinase, catalyzing the final reaction in glycolysis, is encoded by 2 genes in *S. cerevisiae* that encode 2 different proteins. Genomic surveys identified only a single gene in the *P. carinii* genome, which appears to be the standard number in yeast other than *S. cerevisiae.*

**Table 3:** Unigene Representation in the Glycolytic Pathway.

Name of enzyme	Gene Present in Unigene/Genomic Database (U/G/NF)*	Unigene Identifier (s)
Hexokinase	U/G	cDNAv1_0.fasta.screen.Contig604
Phosphoglucose isomerase	G	
6-phosphofructokinase	U/G	14e02uni.f.ab1; 14e02uni.t.ab1
Aldolase	NF	
Triose phosphate isomerase	G	
Glyceraldehyde 3-phosphate dehydrogenase	NF	
Phosphoglycerate kinase	U/G	cDNAv1_0.fasta.screen.Contig 611
Phosphoglycerate mutase	U/G	cDNAv1_0.fasta.screen.Contig993
Enolase	G	
Pyruvate kinase	G	

* Homolog found in Unigene Set (U) or Genomic database (G) or Not Found (NF) in either database.

Oxidative decarboxylation of pyruvate by pyruvate dehydrogenase (EC: 1.2.4.1) is the first step in converting pyruvate to carbon dioxide, linking glycolysis to the TCA cycle. The multi-enzyme complex located in the mitochondrial matrix is comprised of 3 distinct components: pyruvate deydrogenase (E1); dihydrolipoamide acetyl transferase (EC: 2.3.1.12), E2; and dihydrolipoamide deydrogenase (EC: 1.8.1.4), E3. The E2 component was identified in the unigene set while E1 can be found in the *P. carinii* genome sequence, suggesting that *P. carinii* contains this essential enzyme complex. It appears unlikely that *P. carinii* possesses the capacity to perform the “pyruvate bypass” since none of the 3 genes associated with this function were identified in the unigene set or genomic sequences (pyruvate decarboxylase, acetaldehyde deydrogenase, acetyl coA synthetase).

### Pentose phosphate pathway

The pentose phosphate pathway (PPP) is a major pathway for the recycling of NADP+to NADPH and for the production of ribose-5-phosphate necessary for synthesis of nucleotides. An additional role is to protect the cell against reactive oxygen intermediates; a role especially important in a parasitic mode of life. The PPP has both an oxidative and a non-oxidative branch. The *P. carinii* genome contains homologs to all the proteins in the oxidative stage; of which 2 were found as transcripts in the current analysis (Table 4.). The branching of hexose metabolism between the glycolytic and the pentose pathway occurs at the level of glucose-6-phosphate (G6P). In the first step of the oxidative arm, G6P is oxidized to 6-phospho-δ-gluconolactone, resulting in one mole of NADPH. Subsequently, 6-phosphogluconate is decarboxylated by 6-phosphogluconate dehydrogenase (EC 1.1.1.44, *GND1*) to ribulose-5-phosphate and a second mole of NADPH is evolved. A five carbon sugar, D-ribulose-5-phosphate, is produced in this reaction. Ribulose -5-phosphate can either be isomerized to ribose-5-phosphate (R5P) or epimerized to xylulose-5-phosphate. For nucleic acid synthesis, R5P is transformed into 5-phospho-D-ribosyl- 1 pyrophosphoric acid (PRPP) by ribose phosphate diphosphokinase (EC:2.7.6.1), found in the *P. carinii* genome and expressed during infection (Table 4.).

**Table 4:** Unigenes represented in the Pentose Phosphate Pathway

Name of enzyme	Gene Present in Unigene/Genomic Database (U/G/NF)	Unigene Identifier (s)
**Oxidative Stage**		
Glucose-6-phosphate dehydrogenase	G	
Gluconolactonase	G	
6-phosphogluconate dehydrogenase	U/G	cDNAv1_0.fasta.screen.Contig940
Ribose-phosphate pyrophosphokinase	U/G	cDNAv1_0.fasta.screen.Contig616
**Non-oxidative Stage**		
Phosphoglucomutase	G	
Ribulose-5-phosphate 3-epimerase	U/G	cDNAv1_0.fasta.screen.Contig849
		14e02uni.f.abi
		14e02uni.t.abi
Ribose-5-phosphate isomerase	G	
Transketolase	G	
Transaldolase	NF	
Transketolase	G	

* Homolog found in Unigene Set (U) or Genomic database (G) or Not Found (NF) in either database.

The primary role of the non-oxidative arm of the PPP is to produce R5P. Homologs to all but one of the enzyme homologs, transaldolase (*TAL1* in yeast) were identified in the genome and abundant transcripts were observed for the ribulose-5-phosphate 3-epimerase (Table 4.) The epimerase serves to produce xylulose-5-phosphate which in turn donates 2 carbon atoms to R5P, yielding sedoheptulose-7-phosphate and glyceraldehyde 3-phosphate (generated by the transketolase). The transaldolase (missing in the *P. carinii* genome) facilitates the transfer of 3 carbons to glyceraldehyde 3-phosphate from sedoheptulose-7-phosphate to yield fructose-6-phosphate (F6P) and erythrose -4-phosphate. A second molecule of F6P and glyceraldehyde 3-phosphate (GAP) are generated by donation of 2 carbons from a second molecule of xylulose-5-phosphate (generated by transketolase) to erythrose 4-phosphate. The F6P and GAP be used by the cell via glycolysis to produce ATP. The non-oxidative arm of the PPP can work in the reverse by utilizing F6P and GAP to produce R5P via the transaldolase enzyme. Since the *P. carinii* genome does not appear to possess this homolog, reversal of this PPP branch may not be active in *P. carinii.*

### Gluconeogenesis

Gluconeogenesis is the synthesis of glucose from non-carbohydrate precursors such as lactate, glycerol, pyruvate and certain amino acids. It is essentially, a reversal of glycolysis. This is an important function in organisms that must be able to switch their metabolism depending on the carbon sources available. Besides use of alternate carbon sources, enzymes in gluconeogenesis have significant roles in other functions. For example, *PCK1*, a phosphoenolpyruvate carboxykinase involved in gluconeogenesis, has also been found to be essential for sporulation in yeast [42]. The 2 steps unique to gluconeogenesis are the conversion of oxaloacetate to phosphoenolpyruvate via phosphoenolpyruvate carboxykinase; and fructose-1,6-bisphosphatase (*FBP1*), which catalyzes the terminal step of gluconeogenesis and is required for metabolism of every non-sugar carbon source. Though not in the unigene set, the gene homolog to phosphoenolpyruvate carboxykinase was identified in the genomic sequences with highest homology to the *C. albicans* protein. A homolog to *FBP1* was not identified in the unigene or genome databases. Thus, it is questionable if *P. carinii* can carry out this metabolic process. It has been proposed that gluconeogenesis and the glyoxylate cycle (see below) may be important for supplying carbohydrates for cell wall synthesis in other fungi which in turn may be linked to the morphological switch from mycelium to yeast [43]. Certainly, *P. carinii* requires carbohydrates for its cell wall, but to date, a mycelial phase for members of this genus has not been identified.

### TCA cycle

The TCA cycle plays 2 essential roles in cells; its oxidative role is to provide reducing equivalents to drive mitochondrial respiration and thus, produces ATP; and biosynthetic, by contributing the carbon skeletons used in anabolic pathways. Homologs to all the enzymes in the TCA cycle were identified in the *P. carinii* genome as well as transcripts to most (Table 5.). Citrate synthase is present in all cells able to undergo oxidative metabolism. Three genes encoding citrate synthases have been identified in *S. cerevisiae, CIT1*(E.C.: 2.3.3.1) and *CIT3*, that encode mitochondrial enzymes and *CIT2* which encodes an isoenzyme located in the peroxisomes, involved in the glyoxylate cycle. One *P. carinii* unigene contig (contig961) had very high similarities to fungal citrate synthases (*CIT1*), as well as a multitude of corresponding genomic sequences (Table 5.). Contigs 749 and 808 shared high similarities to ATP citrate lyase subunits 1 and 2 (E.C: 2.3.3.8). This 2 subunit protein is encoded by 2 genes in *S. pombe*, neither of which is found in *S. cerevisiae.* A genomic contig in the *P. carinii* genome database appears to encode both subunits. These subunits catalyze the formation of cytosolic acetyl CoA, which is primarily used for the biosynthesis of fatty acids and sterols, rather than entering the TCA cycle. No homologs to either *CIT2* or *CIT3* were identified in either *P. carinii* database. The gene *ACO1* (EC: 4.2.1.3) encodes for the aconitase (a 4Fe-4S cluster containing protein) in *S. cerevisiae.* Homologs to this enzyme were found in the genome and transcriptome of *P. carinii.* . The activity of *ACO1* is dependent upon the gene product encoded by *YFH1*, a ferrochelatase, and a mitochondrial inner membrane protein which catalyzes the insertion of ferrous iron into protoporphyrin IX, the eighth and final step in the heme biosynthetic pathway. A putative *P. carinii YFH1* was identified in the genome database. Alpha-ketoglutarate is transformed into succinyl CoA via the alpha-ketoglutarate dehydrogenase complex. The E1 component of this complex (EC: 1.2.4.2) (*KGD1* in yeast) was identified in the unigene set. Homologs to fungal genes encoding both the E1 and E2 (*KGD2*) components were identified in the *P. carinii* genomic sequences. Two subunits of succinyl CoA synthetase are encoded by the genes *LSC1* and *LSC2* in the *S. cerevisiae* genome. Homologs to both subunits were identified in the genomic database, but no homologs were found in the transcriptome. The action of succinate dehydrognease catalyzes the formation of fumarate. *SDH1-4* encodes tetrameric components of this complex in *S. cerevisiae.* The *P. carinii* unigene set contains a homolog to the succinate dehydrogenase flavoprotein subunit (*SDH1*). *SDH1, 3* and 4 are represented in the genomic sequences. Fumarate hydratase (EC 4.2.1.2) *FUM1*, transforms the fumurate to malate in the subsequent reaction. Only genomic copies of the *P. carinii* homolog to fumarase were identified. In the final step of the TCA cycle, malate dehydrogenase (*MDH1*) converts malate to oxaloacetate which can enter the cycle again. Homologs to this enzyme were identified in both the unigene set and the genomic database.

**Table 5:** Unigenes represented in the Citrate Cycle (TCA).

Name of enzyme	Gene Present in Unigene/Genomic Database (U/G)	Unigene Identifier (s)
Citrate synthase (*CIT1*)	U/G	cDNAv1_0.fasta.screen.Contig961
ATP citrate synthase	U/G	cDNAv1_0.fasta.screen.Contig749
		cDNAv1_0.fasta.screen.Contig808
Aconitate hydratase 1 (aconitase)	U/G	cDNAv1_0.fasta.screen.Contig547
		15h06uni.e.abi
Isocitrate dehydrogenase	G	
Αlpha-ketoglutarate complex	U/G	1h12uni.t.ab1
Succinyl CoA synthetase	G	
Succinate dehydrogenase	U/G	cDNAv1_0.fasta.screen.Contig722
Fumarase	G	
Malate dehydrogenase	U/G	cDNAv1_0.fasta.screen.Contig186
		cDNAv1_0.fasta.screen.Contig846

* Homolog found in Unigene Set (U) or Genomic database (G) or Not Found (NF) in either database.

### Glyoxylate cycle

The glyoxylate cycle, an anaplerotic pathway of the TCA cycle, serves as a shortcut across the citric acid cycle and is used when yeast are grown on 2-carbon sources such as acetate or ethanol or fatty acids as the sole carbon sources. The glyoxylate pathway facilitates the synthesis of C4 dicarboxylic acids from acetyl-coA units, bypassing the 2 decarboxylation steps in the TCA cycle. The cycle is interesting for at least 2 reasons. First, because it is not found in humans it may be targeted for future drug development. Second, this pathway was recently linked to fungal pathogenesis. Many of the genes that were highly induced in phagocytized *S. cerevisiae* were members of the glyoxylate cycle [44], [45], while mutants of the rice blast fungus, *Magnaporthe grisea* lacking isocitrate lyase were unable to germinate and cause disease [46]. Isocitrate lyase is the main controlling protein in the glyoxylate shunt pathway. It hydrolyzes isocitrate to succinate (4 carbons) and glyoxylate (2 carbons). In the second critical step of the glyoxylate cycle, malate synthase condenses acetyl Co-A (2 carbons) with glyoxylate to produce malate (4 carbons). Malate then, is an intermediate in the TCA cycle and is subsequently converted to oxaloacetate, then to citrate and to isocitrate. Searches of both the unigene and genomic sequence databases did not identify any fungal homologs to “citrate lyase” or malate synthase, suggesting that *P. carinii* may not have a glyoxylate cycle. Further evidence that *P. carinii* does not possess a glyoxylate cycle include the absence of homologs to the genes encoding peroxisomal receptors, *PEX5* and *PEX7* [47], which facilitate the importation of the CIT2 protein into yeast peroxisomes, and the transcription factors, *RTG1* and *RGT2*, which control *CIT2* expression [48]. *P. carinii* may resemble *S. pombe* more in this respect than *S. cerevisiae.* Although the genome of *S. pombe* contains a homolog to yeast isocitrate lyase, it is likely to be involved in amino acid biosynthesis, rather than in the glyoxylate pathway since it does not have any of the other peroxisomal glyoxylate pathway enzymes (http://www.genedb.org/genedb/pombe/).

### Pathways of interest, not included in the automated analysis

In earlier studies, the presence of synaptonemal complexes shown by transmission electron microscopy provided evidence that *Pneumocystis* could replicate by a sexual process [49]. More recently, homologs to genes used in the mating process by other fungi such as the *STE3* receptor [27] [28] were identified in the genome of *P. carinii.* Analysis of the unigene database identified additional genes that are associated with mating or the sexual mode of replication in fungi, shown in Table 6. The numerous transcripts identified for these processes in the unigene set suggest that *P. carinii* may undergo sexual replication within the mammalian host in the context of infection. That this process is stimulated by stress or nutritional deprivation is supported by the abundant transcripts associated with these processes (Table 1.).

**Table 6:** Gene Homologs associated with mating/meiosis.

Fungal homolog	*P. carinii* unigene	Function
MATMC, *Schizosaccharomyces pombe*	*cDNAv1_0.fasta.screen.Contig913*	Mating-type M-specific polypeptide Mc
FAR10, *Saccharomyces cerevisiae*	*cDNAv1_0.fasta.screen.Contig439*	involved in G1 cell cycle arrest in response to pheromone
STE3, *Saccharomyces cerevisiae*	*cDNAv1_0.fasta.screen.Contig638*	mating-type a-factor pheromone receptor
STE11, *Pneumocystis carinii*	*cDNAv1_0.fasta.screen.Contig1031*	Signal transducing MEK kinase involved in pheromone response and pseudohyphal/invasive growth pathways
CDC31, *Schizosaccharomyces pombe*	*cDNAv1_0.fasta.screen.Contig97*	required for spindle pole body duplication in mitosis and meiosis II
FUS3, *Aspergillus fumigatus*	*cDNAv1_0. fasta.screen.Contig541*	Mitogen-activated protein kinase involved in mating pheromone response
SHO1, *Candidda albicans*	*cDNAv1_0.fasta.screen.Contig258*	Transmembrane osmosensor, participates in activation of both the Cdc42p-and MAP kinase-dependent filamentous growth pathway; mating tip projection pathway
ORC3, *Schizosaccharomyces pombe*	*cDNAv1_0.fasta.screen.Contig295*	chromatin silencing at silent mating-type cassette
ORC5, *Schizosaccharomyces pombe*	*cDNAv1_0.fasta.screen.Contig699*	chromatin silencing at silent mating-type cassette
CDC10, *Yarrowia lipolytica*	*cDNAv1_0.fasta.screen.Contig211*	cellular morphogenesis during conjugation with cellular fusion; establishment of cell polarity (sensu Fungi); spore wall assembly (sensu Fungi)
SPT7, *Saccharomyces cerevisiae*	*cDNAv1_0.fasta.screen.Contig898*	Subunit of the SAGA transcriptional regulatory complex conjugation with cellular fusion
MEU26, *Schizosaccharomyces pombe*	*cDNAv1_0.fasta.screen.Contig689*	meiotic expression upregulated; no apparent S. *cerevisiae* ortholog
DEP1, *Saccharomyces cerevisiae*	*cDNAv1_0. fasta.screen.Contig663*	Transcriptional modulator involved in maintenance of telomeres, mating efficiency and sporulation
MAM2, *Schizosaccharomyces pombe*	*cDNAv1_0.fasta.screen.Contig632*	Pheromone P-factor receptor

Another pathway that was represented in the transcriptome was that of sterol biosynthesis. This pathway is especially interesting in these organisms because ergosterol, the predominant bulk sterol found in most fungi and the target of most anti-fungal therapies, cannot be detected in any member of the genus. Instead, *Pneumocystis spp.* appear to use cholesterol as their primary sterol. Evidence includes biochemical analyses [50], [51], in vitro inhibition assays [52], and more recently, molecular analyses of the genome [53]–[55]. *Pneumocystis* pneumonia is recalcitrant to treatment with standard anti-fungal therapies that target ergosterol synthesis, which raises important questions as to the function of an operational sterol biosynthetic pathway in these organisms. A homolog to the first committed enzyme in the sterol biosynthetic pathway, *ERG1* (squalene epoxidase), was identified in the unigene set (Contigs 35 and 970). An earlier study identified the enzyme responsible for the next 2 reactions that produce lanosterol, *ERG7*, in the ESTs and its cDNA sequence [56]. The investigators of this study showed the enzyme was biochemically active using a recombinant protein. The demethylation of lanosterol is catalyzed by lanosterol demethylase (Erg11p). Erg11p is the target of the azole family of anti-fungal compounds. No transcript was identified for this gene, although a copy is present in the genomic database. The cDNA of the *P. carinii Erg11* gene was able to complement the deletion of this gene in yeast [54], suggesting it too may function at some time in the organisms' life cycle. Immediately downstream, actions of the ERG24p (C-14 sterol reductase) together with ERG25p, ERG26p, and ERG27p result in the synthesis of zymosterol, the substrate for the C-24 sterol methyltransferase, ERG6p. Homologs to *ERG24* and *ERG6* were present in the unigene database, while homologs to *ERG* 26 and 27 were found in the genome database. Since ergosterol is not the end product of sterol biosynthesis in *P. carinii,* but a homolog to the ERG4 gene is present in its genomic database, it will be interesting to identify the function of these enzymes during the infective process, since they could be potential drug targets.

## Discussion

Transcriptional analysis based on a non-normalized EST library is limited by the under representation of rare transcripts and the over representation of abundant messages. Nonetheless, it permits a look into the putative processes undertaken by an organism under a set of specific conditions. In the case of an uncultivable pathogen like *P. carinii,* analysis of gene expression during the infective process provides significant information about the metabolic processes used for basic growth functions as well as specialized processes that may permit it to adapt to the hostile environment of the host. In the mammalian lung, *P. carinii* faces significant challenges to its survival. Although fulminate infections result from a profoundly suppressed host immune system, some immune function is retained and the organisms must evade this response. At the same time, the pathogen must seek and take in nutrients; grow and replicate in this inhospitable environment.

The results presented here show that there is a wide variety of metabolic processes that are likely to be active in *P. carinii* during infection. It should be noted that there are likely to be other metabolic cycles active under these conditions that were not identified by the KASS analysis which only included 39% of the unigenes, and also due to our relative conservative inclusion criteria for putative *P. carinii* homologs detailed in the Methods section. In addition, the presence of splice intermediates in several genes as evidenced by preliminary analysis of cDNA and genomic DNA alignments may also contribute to an increased metabolic repertoire for these organisms. Intron/exon studies are ongoing and will be reported in another communication.

Analysis of the most abundant transcripts expressed by *P. carinii* at the time of isolation from an immunosuppressed rat with a fulminate infection revealed a multitude of gene homologs associated with stress responses as well as those involved in gene transcription, protein synthesis, and replication. The expression of genes involved in stress responses likely results from a variety of different stimuli. A rat with a fulminate infection is quite moribund as the organisms have reached very high numbers within the entire lung, invading almost every alveolar lumen. Respiration is shallow and oxygenation poor. It is likely this milieu results in nutritional limitation for the organisms which are packed layer upon layer within the alveoli. Genes such as *STE11* and *SFP1* are expressed in response to nutritional limitations [57], [58]. In the case of *STE11*, this can also lead to expression of genes associated with meiosis or hyphal growth in *S. cerevisiae* (*STE12*)[59]. The process of sporogenesis is associated with meiosis in ascosporogenous fungi, and the *CON7* gene, associated with this process in *Magnaporthe* was identified as an abundant transcript. The Pneumocystis-specific *MSG* genes were also expressed in high abundance. It is well known that the *MSG* gene family encodes a multitude of surface glycoproteins that have adhesion qualities. It has been postulated that a function of these adhesins may be similar to the *FLO* genes of *S. cerevisiae* [60], which also encode surface glycoproteins that promote cell to cell interactions, especially in a nutritionally depleted environment. Thus, it is plausible that *P. carinii* undergoes sexual replication stimulated by the nutritionally poor environment at end stage disease. A second stimulus for the stress responses may be a consequence of oxidative stress due to the production of reactive oxygen species by the host's immune cells. The *PDR3* [61]and *SIN1* [62]genes are associated with oxidative stress in other fungi . The *HSP90* gene of *S. pombe* (also called *CDC37*) was specifically associated with the positive regulation of a stress activated protein kinase (SAPK) that plays a crucial role in cellular survival to inflammatory responses [63]. Although *P. carinii* expressed superoxide dismutase (Contig346) and catalase (Contig327), they were not found as abundant transcripts, and thus the organisms could have experienced oxidative cell damage. It is interesting to note that a homolog to transaldolase (*TAL*) is apparently absent from the transcriptome and the genome of *P. carinii.* This is the key enzyme in the reversible non-oxidative branch of the PPP that is responsible for generation of NADPH. A primary function of the PPP is to maintain glutathione in a reduced state, which functions to provide protection of sulfhydryl groups and cellular integrity from oxygen radicals. If the reversible nature of the PPP is compromised by the absence of the transaldolase, the ability to fully ward off the detrimental effects of the reactive species may be attenuated, leading to the observed stress reaction. The third stimulus for the stress responses could have arisen from the lengthy purification process used to extricate the organisms from host lung tissue [64], [65]. This process involves the use of mechanical disruption of lung tissue with a tissue homogenizer and processing over a 3–4 hour period.

The majority of transcripts associated with metabolic cycles were dedicated to carbohydrate metabolism, specifically glycolysis. Glycolysis has been shown to be essential for growth in the mammalian host by a number of fungal pathogens. Recently, it was shown that the energy production strategies used by *C. albicans* changed in response to physiologically distinct host niches [66]. Gluconeogenic and glyoxylate–associated genes were active early in the infection when the yeast were phagocytized by host cells, but progression of systemic disease was dependent upon glycolysis. The authors postulated that the nutritionally poor environment of the phagocyte reflected starvation conditions that stimulated the alternative pathways. The emphasis on expression of glycolytic enzymes by *P. carinii* and not those in alternative pathways, suggests that although the alveolar compartment may immunologically challenge the organisms, the milieu may provide a sufficient nutritional environment, circumventing the need for alternative carbohydrate pathways. The paucity of homologs in the gluconeogenesis and glyoxylate pathways implies that these organisms may not be able to utilize non-fermentable carbon sources. This may have been an adaptation to the host environment that occurred during the evolution of the host-parasite relationship. It is also well known that Pneumocystis are easily phagocytized and killed by macrophages, suggesting a lack of survival strategy in this compartment of the immune response.

The apparent lack of a transaldolase homolog in the PPP may have effects on the organisms' ability to respond to oxidative damage (discussed above), but may also limit an alternative mode for ATP production via glycolysis by blocking the synthesis of F6P and GAP. Similarly, non-reversibility of the PPP would also reduce the ability to synthesize R5P from F6P and GAP obtained from glycolysis. It is notable that the genome of *Plasmodium falciparum,* another host-dependent parasite lacks a homolog to transaldolase as well [67].

Whether *P. carinii* is able to undergo fermentation remains a question. The characteristics of fermentation are at least 3 of the following: the release of energy from a sugar or other organic compound; no requirement for molecular oxygen; no requirement for an electron transport system; or use of an organic compound as the final electron acceptor. Fermentation in yeast uses the same processes as glycolysis, except in the absence of oxygen, it is blocked from entering the TCA cycle and thus converts the pyruvate to acetaldehyde via pyruvate decarboxylase, and then to ethanol via alcohol dehydrogenase, losing one carbon in the process that evolves as carbon dioxide. The evidence at hand that argues against fermentation by *P. carinii* includes the lack of a homolog to pyruvate dehydrogenase in the unigene set or the genomic database and its inability to survive under anaerobic conditions. Recent studies have shown that *P. carinii* rapidly loses viability in an anaerobic atmosphere and seems to require some oxygen, although it is able to survive in an atmosphere of reduced oxygen levels [68]. On the other hand, its genome contains 3 genes encoding putative alcohol dehydrogenases. In *S. cerevisiae*, there are five genes that encode alcohol dehydrogenases involved in ethanol metabolism, *ADH1* to *ADH5*. Four of these enzymes, ADH1p, ADH3p, ADH4p, and ADH5p, reduce acetaldehyde to ethanol during glucose fermentation, while ADH2p catalyzes the reverse reaction of oxidizing ethanol to acetaldehyde. Homologs to *ADH1, ADH2* and *ADH3* were identified in the *P. carinii* genomic database. In contrast, the genome of the fission yeast Schizosaccharomyces pombe, contains only one alcohol dehydrogenase gene, adh1(+), and is able to ferment [69]. It is clear that use of respiration or fermentation by yeasts is regulated by the availability of glucose and oxygen. Some yeasts are obligate respirers like the Cryptococcus species which are incapable of fermentation or anaerobic growth [35]. Others, like species of Candida, Kluveromyces and Pichia can respire anaerobically, but fermentation only occurs in pre-grown cells and they are not able to grow anaerobically. *S. pombe* is capable of aerobic fermentation, but also cannot grow under anaerobic conditions. At the other end of the spectrum, Torulopsis are obligate fermenters and cannot respire, but grow and ferment only under anaerobic conditions. The most versatile of the group is *S. cerevisiae,* which is considered a facultative aerobic fermenter that can ferment under both aerobic and anaerobic conditions and is capable of facultative growth under anaerobic conditions. Without direct experimental data, it is difficult to place *P. carinii* within its proper category, but assuming it cannot grow without a source of molecular oxygen, it would seem to be either an obligate respirer or perhaps a facultative aerobic fermenter, depending on the function of its alcohol dehydrogenases.

The entire life cycle of any member of the genus Pneumocystis has not been defined. Microscopic observations of organisms within the alveoli have led to many proposed life cycles that focus on development only within the lung [7]. Most agree that there is an asexual cycle that consists of the smaller trophic forms dividing by binary fission and a phase which results in formation of a cyst or ascus which contains 8 spores or daughter forms. The process used by the organisms to produce the cyst and spores has been hypothesized to be through a sexual process, although there is not full consensus on this point. Several genes related to the sexual reproductive cycle were identified in the transcriptome of *P. carinii,* suggesting that sexual reproduction may occur in the mammalian lung during active infection. If this assumption is correct, it is in striking contrast to most other fungal pathogens causing human diseases that do not undergo sexual reproduction in their respective hosts, such as Cryptococcus spp., *Blastomyces dermatiditis,* Coccidioides spp., *Histoplasma capsulatum, Penicillium marnefeii,* Candida spp. or *Paracoccidioides brasiliensis.* With the exception of *Candida* spp., these and other fungal pathogens are not considered normal flora or commensals of the immunologically intact host. Moreover, most of these fungi have a primary environmental niche in which sex, if it does occur, takes place. *P. jirovecii* have been reported to “colonize” certain sectors of the human population [19], [70] and *P. carinii* are frequently detected in the lungs of healthy adult rodents [30], [71] and within hours after birth in neonatal rats [72]. The serological responses to *P. jirovecii* antigens early in life are also indicative of their role as normal flora [73]. There is no known environmental habitat for Pneumocystis, and thus it would follow that if sex does occur, it would necessarily take place in the mammalian host. A further distinction between Pneumocystis spp. and most other medically important fungal pathogens is communicability of the infection. Most fungal infections are acquired by inhalation of infectious propagules, by deep or superficial wound trauma, or other environmental exposures and terminate in the infected host. There is no transmission. In contrast, Pneumocystis appears to be a highly transmissible infection as evidenced by serological responses and extensive experimental transmission studies. In that sense, Pneumocystis are transmitted directly to the next host, much like Candida spp., but by an airborne rather than contact route. Thus, members of Pneumocystis spp. are distinct from other medically significant fungi in that they appear to undergo sexual replication in their mammalian hosts and are able to transmit the infection from host to host.

We posit that through the process of evolution, Pneumocystis spp. have adapted to their specific mammalian hosts to form a sustainable relationship. In many microbial infections, the host: parasite relationship is defined by the virulence factors produced by the parasite and the resistant counter defenses by the host. This is largely due to the sophisticated immune surveillance systems and innate defense mechanisms by the host and the lack of adaptation of many microbial pathogens, resulting in a tug of war between resistance and virulence. However, that relationship is just one of a continuum of combinations among two species. The concept of “compatibility” is emerging as an alternative to the standard mode of thinking where there is a constant battle of the invader vs prey. In the fungal plant pathogen field, “compatibility” is defined as the complementary relationship between a plant species and an adapted pathogen species that underlies susceptibility and ultimately results in disease [74]. Biotrophic fungi derive their energy from the living cells of their plant hosts without loss of host cell viability. Obligate biotrophs complete their entire life cycle within the plant host, including the sexual cycle, and are incapable of ex vivo growth or limited in vitro cultivation, like Pneumocystis. It is generally accepted that members of the genus Pneumocystis in the immunologically intact mammalian host, cause little or no clinical disease. It is only when the host tips the balance toward these organisms by losing immune capability to control the organisms that disease ensues. This would infer there may be“compatibility” between the mammalian host and its resident Pneumocystis species. Seen in this light, Pneumocystis are quite accommodating parasite partners. There is no invasive entry into the host; rather it is likely through a passive inhalation of spores wafting in the air. Once inside the lung alveoli of the immune intact host, there is no massive infection, rapid reproduction, or invasive process. Although these early steps are poorly understood, it appears likely that a cryptic, low level infection ensues which the host immune system recognizes by humoral and cellular means. Indeed, the presence of only a single copy of the rDNA locus has been hypothesized to permit slow growth of the organisms [60], which also could serve to maintain the balance of host and pathogen. As a counter defense to immune recognition, all members of Pneumocystis spp. surveyed to date contain a multigene family capable of encoding perhaps hundreds of isotypes of their major surface antigens, the Major Surface Glycoproteins (*MSG*) [75]. Switching of the surface antigen coat provides a survival strategy similar to that used by other microbial pathogens, such as the African trypanosomes. The length of time that the host: Pneumocystis tête á tête may last is not presently known, but it appears that there may be a constant re-infection of the hosts by Pneumocystis without serious harm to the immune intact host. Molecular evidence presented in the present report suggests that *P. carinii* is capable of a wide variety of metabolic processes, which would also accommodate its stay in the host. Without the need to pirate host metabolic products or processes by Pneumocystis, the inter-relationship between the 2 species would not result in death of the host or expulsion of the parasite, leading to a sustainable situation. And like the biotrophic plant fungi, the entire life cycle appears to take place in the host and they are also uncultivable outside the host.

The pathology created by Pneumocystis during active infection in the immunocompromised host is due to the mechanical obstruction of the air-gas interchange of the host, leading to decreased oxygenation and to the inflammatory response by the host, largely controlled by the CD8 cell infiltration [76]. The Pneumocystis organisms have not been reported to secrete virulence factors and likely do not facilitate the infection by these means. The last piece of evidence regarding the adaptation of the members of the Pneumocystis spp. is their host specificity. Most mammalian species harbor at least one species of Pneumocystis which is genetically and phenotypically distinct from those members in other mammalian hosts. This would infer an adaptation that occurred over millions of years, resulting in an almost commensal relationship with each host. This adaptation is in stark contrast to most of the fungal pathogens discussed above that do not depend on the host for survival.

Many of the genes expressed by *P. carinii* were organism-specific. The *MSG, MSR*, and the family of proteases that are thought to be necessary for processing of the surface antigens were highly expressed. The MSGp are known to bind extra cellular matrix proteins like vitronectin and fibronectin [77]–[79]; aid in adherence to the Type I pneumocyte in the alveoli [80]; and also aid in adherence of the organisms to one another [60]. Thus it is likely that these glycoproteins perform many functions that are necessary for the infective process and may contribute to the adaptation process.

*P. carinii* is not an opportunistic pathogen in the fungal pathogen sense of the word. The mammalian host appears to be necessary for its survival and life cycle. In what may be its natural host, the immunologically intact mammal, it exerts little to no pathogenic effects. Loss of immune competence permits the organisms to grow, resulting in pathogenesis. The apparent presence of a large number of metabolic pathways as detected by the present analysis, the slow growth of the organisms, its host specificity and the lack of pathogenesis imply that these organisms have adapted to form a compatible relationship within their natural habitat, the immune intact mammalian host.

## Supporting Information

Figure S1 Annotation distribution of ESTs. The number of ESTs that were assigned to GO categories.
(0.23 MB TIF)

Figure S2 Binning of *P. carinii* unigene homologs by general organism groups. The unigenes were analyzed for similarities to gene products of other organisms using BLASTx. Significance was set at E≤10-6.
(0.08 MB TIF)

Figure S3 Unigenes were analyzed using the BLAST2GO software. Shown are level 2 categories for Biological Processes
(0.41 MB TIF)

Table S1 EST BLAST2GO Biological Processes-All Levels. All hierarchical levels of BLAST2GO Biological Processes for the ESTs.
(0.30 MB XLS)

Table S2 EST BLAST2GO Molecular Function-All levels. Hierarchical distribution of the Molecular Function of the ESTs using BLAST2GO.
(0.15 MB XLS)

Table S3 EST BLAST2GO Cellular Compartmentalization Assignments-All levels. Hierarchical distribution of the cellular compartmentalization assignments of the ESTs by BLAST2GO.
(0.08 MB XLS)

Table S4 Unigene Population of KEGG Pathways
(0.10 MB DOC)

Table S5 Orthology List for Unigenes in KEGG pathways.
(0.24 MB DOC)
