# Supplementary figures and images for: Transcriptome of Pneumocystis carinii during Fulminate Infection: Carbohydrate Metabolism and the Concept of a Compatible Parasite
Source: PLoS One. 2007 May 9;2(5):e423. doi: 10.1371/journal.pone.0000423 (PMC1855432; doi:10.1371/journal.pone.0000423)

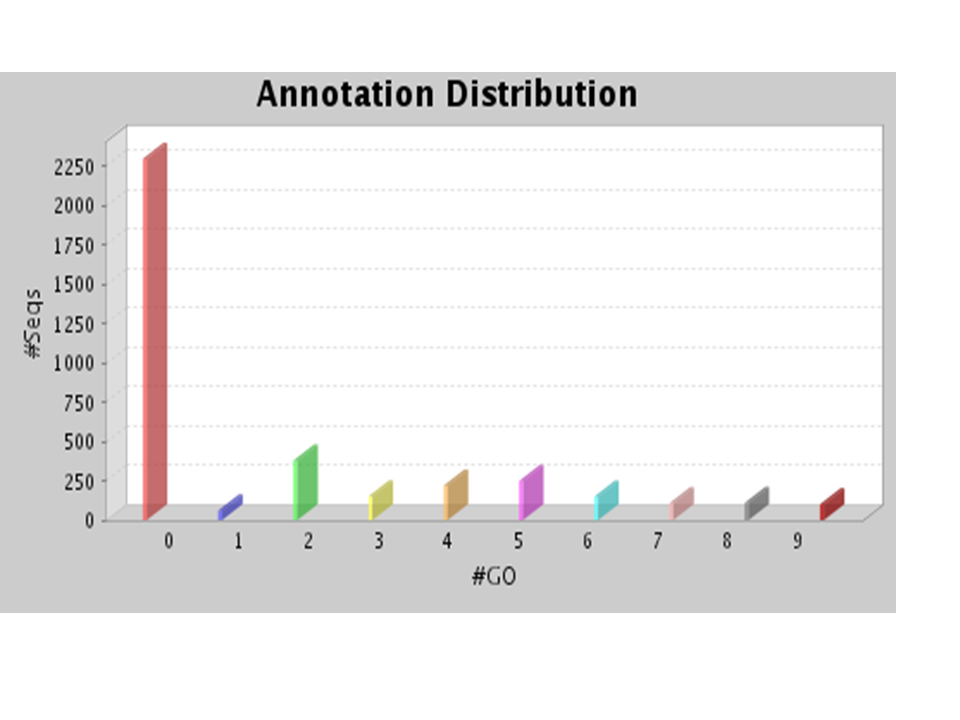

Supplement: Figure S1 — Annotation distribution of ESTs. The number of ESTs that were assigned to GO categories. (0.23 MB TIF) [file pone.0000423.s001.tif]

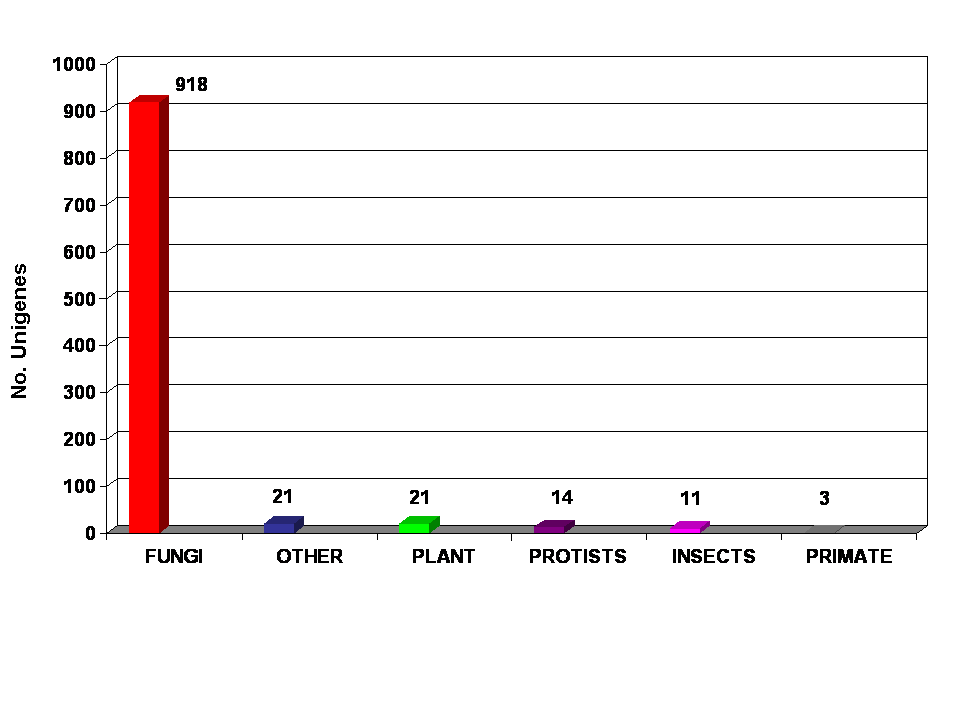

Supplement: Figure S2 — Binning of P. carinii unigene homologs by general organism groups. The unigenes were analyzed for similarities to gene products of other organisms using BLASTx. Significance was set at E≤10-6. (0.08 MB TIF) [file pone.0000423.s002.tif]

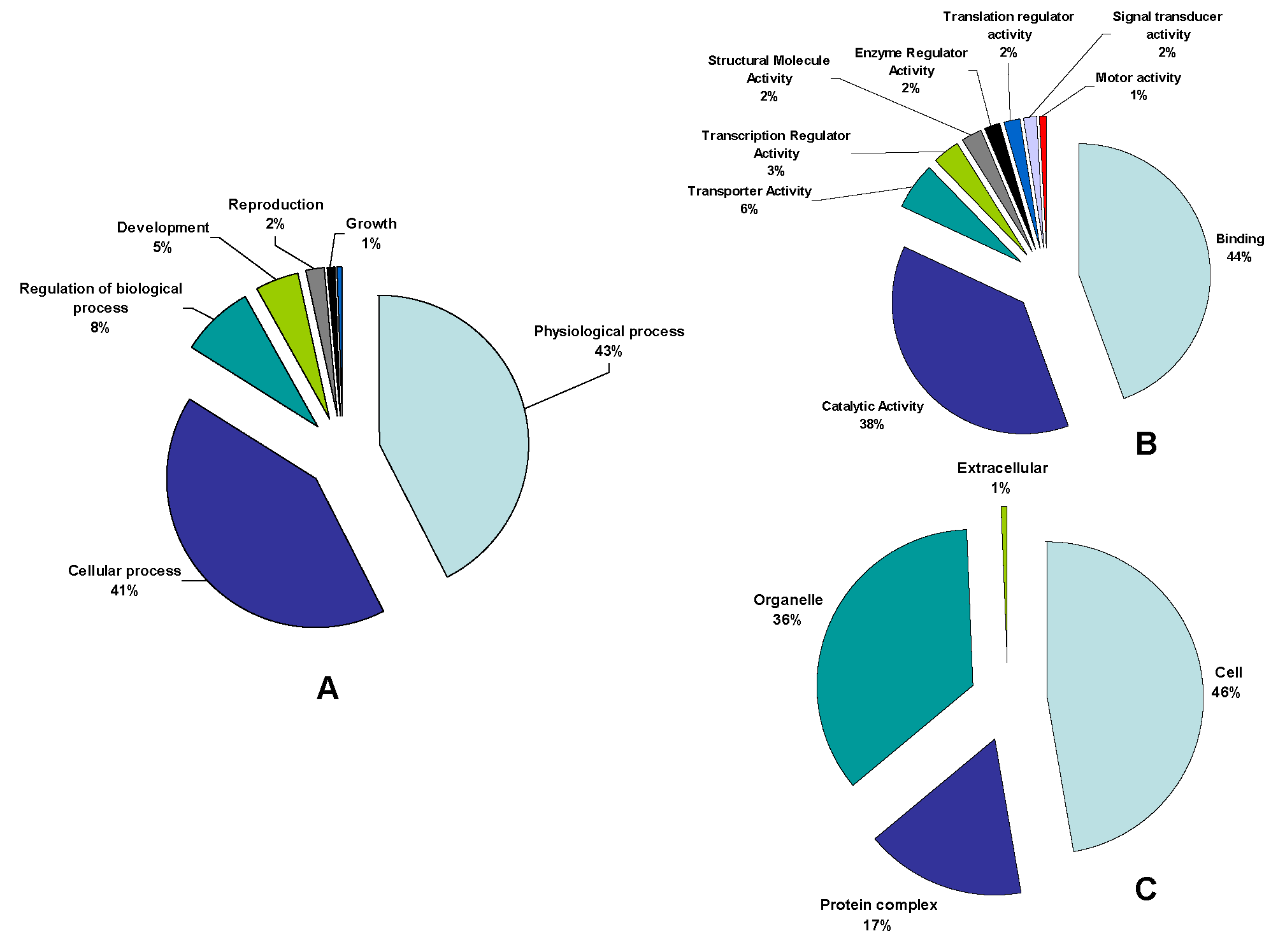

Supplement: Figure S3 — Unigenes were analyzed using the BLAST2GO software. Shown are level 2 categories for Biological Processes (0.41 MB TIF) [file pone.0000423.s003.tif]
