# Supplementary material for: Transcriptome of Pneumocystis carinii during Fulminate Infection: Carbohydrate Metabolism and the Concept of a Compatible Parasite
Source: PLoS One. 2007 May 9;2(5):e423. doi: 10.1371/journal.pone.0000423 (PMC1855432; doi:10.1371/journal.pone.0000423)
Supplement: Table S4 — Unigene Population of KEGG Pathways (0.10 MB DOC) [file pone.0000423.s007.doc]

**01110 Carbohydrate Metabolism**

[00010](http://www.genome.jp/kegg-bin/mark_pathway_www?@map00010/reference%3Dwhite/default%3D%23bfffbf/K00844/K00850/K00927/K01834/K00162/K00627/K00001/K01835) Glycolysis / Gluconeogenesis [PATH:ko00010] [GO:0006096 0006094] (8)

[00020](http://www.genome.jp/kegg-bin/mark_pathway_www?@map00020/reference%3Dwhite/default%3D%23bfffbf/K01647/K01648/K01681/K00164/K00239/K00026) Citrate cycle (TCA cycle) [PATH:ko00020] [GO:0006099] (6)

[00030](http://www.genome.jp/kegg-bin/mark_pathway_www?@map00030/reference%3Dwhite/default%3D%23bfffbf/K00033/K01783/K00850/K01835/K00948) Pentose phosphate pathway [PATH:ko00030] [GO:0006098] (5)

[00040](http://www.genome.jp/kegg-bin/mark_pathway_www?@map00040/reference%3Dwhite/default%3D%23bfffbf/K00963/K01783) Pentose and glucuronate interconversions [PATH:ko00040] (2)

[00051](http://www.genome.jp/kegg-bin/mark_pathway_www?@map00051/reference%3Dwhite/default%3D%23bfffbf/K00966/K00844/K00850) Fructose and mannose metabolism [PATH:ko00051] [GO:0006000 0006013] (3)

[00052](http://www.genome.jp/kegg-bin/mark_pathway_www?@map00052/reference%3Dwhite/default%3D%23bfffbf/K00963/K01835/K00844/K00850) Galactose metabolism [PATH:ko00052] [GO:0006012] (4)

[00500](http://www.genome.jp/kegg-bin/mark_pathway_www?@map00500/reference%3Dwhite/default%3D%23bfffbf/K00963/K01835/K00844/K00688/K01529) Starch and sucrose metabolism [PATH:ko00500] (5)

[00530](http://www.genome.jp/kegg-bin/mark_pathway_www?@map00530/reference%3Dwhite/default%3D%23bfffbf/K01836/K00820/K00844) Aminosugars metabolism [PATH:ko00530] (3)

[00520](http://www.genome.jp/kegg-bin/mark_pathway_www?@map00520/reference%3Dwhite/default%3D%23bfffbf/K00963) Nucleotide sugars metabolism [PATH:ko00520] (1)

[00620](http://www.genome.jp/kegg-bin/mark_pathway_www?@map00620/reference%3Dwhite/default%3D%23bfffbf/K00162/K00627/K00026) Pyruvate metabolism [PATH:ko00620] [GO:0006090] (3)

[00630](http://www.genome.jp/kegg-bin/mark_pathway_www?@map00630/reference%3Dwhite/default%3D%23bfffbf/K00026/K01647/K01681) Glyoxylate and dicarboxylate metabolism [PATH:ko00630] (3)

[00650](http://www.genome.jp/kegg-bin/mark_pathway_www?@map00650/reference%3Dwhite/default%3D%23bfffbf/K00239/K01641/K00162) Butanoate metabolism [PATH:ko00650] (3)

[00562](http://www.genome.jp/kegg-bin/mark_pathway_www?@map00562/reference%3Dwhite/default%3D%23bfffbf/K00888) Inositol phosphate metabolism [PATH:ko00562] (1)

**01120 Energy Metabolism**

[00190](http://www.genome.jp/kegg-bin/mark_pathway_www?@map00190/reference%3Dwhite/default%3D%23bfffbf/K00334/K00336/K00338/K03937/K03946/K03885/K00239/K02301/K02258/K01507) Oxidative phosphorylation [PATH:ko00190] (10)

[00193](http://www.genome.jp/kegg-bin/mark_pathway_www?@map00193/reference%3Dwhite/default%3D%23bfffbf/K02112/K02117/K02120/K02154/K02144) ATP synthesis [PATH:ko00193] [TC:3.A.2] (5)

[00710](http://www.genome.jp/kegg-bin/mark_pathway_www?@map00710/reference%3Dwhite/default%3D%23bfffbf/K00927/K01783/K00813/K00026) Carbon fixation [PATH:ko00710] (4)

[00720](http://www.genome.jp/kegg-bin/mark_pathway_www?@map00720/reference%3Dwhite/default%3D%23bfffbf/K00026/K00239/K01681) Reductive carboxylate cycle (CO2 fixation) [PATH:ko00720] (3)

[00680](http://www.genome.jp/kegg-bin/mark_pathway_www?@map00680/reference%3Dwhite/default%3D%23bfffbf/K03782) Methane metabolism [PATH:ko00680] (1)

[00910](http://www.genome.jp/kegg-bin/mark_pathway_www?@map00910/reference%3Dwhite/default%3D%23bfffbf/K01915) Nitrogen metabolism [PATH:ko00910] (1)

[00920](http://www.genome.jp/kegg-bin/mark_pathway_www?@map00920/reference%3Dwhite/default%3D%23bfffbf/K01738) Sulfur metabolism [PATH:ko00920] (1)

**01130 Lipid Metabolism**

[00061](http://www.genome.jp/kegg-bin/mark_pathway_www?@map00061/reference%3Dwhite/default%3D%23bfffbf/K01946/K00647/K00059) Fatty acid biosynthesis [PATH:ko00061] (3)

[00071](http://www.genome.jp/kegg-bin/mark_pathway_www?@map00071/reference%3Dwhite/default%3D%23bfffbf/K00001) Fatty acid metabolism [PATH:ko00071] (1)

[00072](http://www.genome.jp/kegg-bin/mark_pathway_www?@map00072/reference%3Dwhite/default%3D%23bfffbf/K01641) Synthesis and degradation of ketone bodies [PATH:ko00072] (1)

[00100](http://www.genome.jp/kegg-bin/mark_pathway_www?@map00100/reference%3Dwhite/default%3D%23bfffbf/K00801/K00511) Biosynthesis of steroids [PATH:ko00100] (2)

[00120](http://www.genome.jp/kegg-bin/mark_pathway_www?@map00120/reference%3Dwhite/default%3D%23bfffbf/K00001) Bile acid biosynthesis [PATH:ko00120] (1)

[00561](http://www.genome.jp/kegg-bin/mark_pathway_www?@map00561/reference%3Dwhite/default%3D%23bfffbf/K00001/K00864/K01046) Glycerolipid metabolism [PATH:ko00561] [GO:0045017 0046486 0046503] (3)

[00564](http://www.genome.jp/kegg-bin/mark_pathway_www?@map00564/reference%3Dwhite/default%3D%23bfffbf/K00111/K01115/K00998/K01613) Glycerophospholipid metabolism [PATH:ko00564] [GO:0006650 0046474 0046475] (4)

**01140 Nucleotide Metabolism**

[00230](http://www.genome.jp/kegg-bin/mark_pathway_www?@map00230/reference%3Dwhite/default%3D%23bfffbf/K00948/K01588/K00088/K01951/K00525/K00856/K00939/K01509/K01553/K01939) Purine metabolism [PATH:ko00230] (10)

[00240](http://www.genome.jp/kegg-bin/mark_pathway_www?@map00240/reference%3Dwhite/default%3D%23bfffbf/K00609/K00384/K00525/K01493) Pyrimidine metabolism [PATH:ko00240] (4)

**01150 Amino Acid Metabolism**

[00251](http://www.genome.jp/kegg-bin/mark_pathway_www?@map00251/reference%3Dwhite/default%3D%23bfffbf/K00813/K01885/K00820/K01915/K01920/K01951) Glutamate metabolism [PATH:ko00251] (6)

[00252](http://www.genome.jp/kegg-bin/mark_pathway_www?@map00252/reference%3Dwhite/default%3D%23bfffbf/K00813/K00609/K01939) Alanine and aspartate metabolism [PATH:ko00252] (3)

[00260](http://www.genome.jp/kegg-bin/mark_pathway_www?@map00260/reference%3Dwhite/default%3D%23bfffbf/K00639/K01880/K00998/K01613/K01697/K01875) Glycine, serine and threonine metabolism [PATH:ko00260] (6)

[00271](http://www.genome.jp/kegg-bin/mark_pathway_www?@map00271/reference%3Dwhite/default%3D%23bfffbf/K01697/K01874) Methionine metabolism [PATH:ko00271] (2)

[00272](http://www.genome.jp/kegg-bin/mark_pathway_www?@map00272/reference%3Dwhite/default%3D%23bfffbf/K01883/K01738/K00813) Cysteine metabolism [PATH:ko00272] (3)

[00280](http://www.genome.jp/kegg-bin/mark_pathway_www?@map00280/reference%3Dwhite/default%3D%23bfffbf/K01641) Valine, leucine and isoleucine degradation [PATH:ko00280] (1)

[00290](http://www.genome.jp/kegg-bin/mark_pathway_www?@map00290/reference%3Dwhite/default%3D%23bfffbf/K00162) Valine, leucine and isoleucine biosynthesis [PATH:ko00290] (1)

[00300](http://www.genome.jp/kegg-bin/mark_pathway_www?@map00300/reference%3Dwhite/default%3D%23bfffbf/K01439/K05822/K04567) Lysine biosynthesis [PATH:ko00300] (3)

[00310](http://www.genome.jp/kegg-bin/mark_pathway_www?@map00310/reference%3Dwhite/default%3D%23bfffbf/K00164/K01423) Lysine degradation [PATH:ko00310] (2)

[00330](http://www.genome.jp/kegg-bin/mark_pathway_www?@map00330/reference%3Dwhite/default%3D%23bfffbf/K00813/K01881) Arginine and proline metabolism [PATH:ko00330] (2)

[00350](http://www.genome.jp/kegg-bin/mark_pathway_www?@map00350/reference%3Dwhite/default%3D%23bfffbf/K00813/K00001) Tyrosine metabolism [PATH:ko00350] (2)

[00360](http://www.genome.jp/kegg-bin/mark_pathway_www?@map00360/reference%3Dwhite/default%3D%23bfffbf/K00813) Phenylalanine metabolism [PATH:ko00360] (1)

[00380](http://www.genome.jp/kegg-bin/mark_pathway_www?@map00380/reference%3Dwhite/default%3D%23bfffbf/K00486/K00164/K03782) Tryptophan metabolism [PATH:ko00380] (3)

[00400](http://www.genome.jp/kegg-bin/mark_pathway_www?@map00400/reference%3Dwhite/default%3D%23bfffbf/K01817/K00813) Phenylalanine, tyrosine and tryptophan biosynthesis [PATH:ko00400] (2)

**01160 Metabolism of Other Amino Acids**

[00450](http://www.genome.jp/kegg-bin/mark_pathway_www?@map00450/reference%3Dwhite/default%3D%23bfffbf/K01697/K01874/K01738) Selenoamino acid metabolism [PATH:ko00450] (3)

[00480](http://www.genome.jp/kegg-bin/mark_pathway_www?@map00480/reference%3Dwhite/default%3D%23bfffbf/K01256/K01920) Glutathione metabolism [PATH:ko00480] [GO:0006749] (2)

**01170 Glycan Biosynthesis and Metabolism**

[00510](http://www.genome.jp/kegg-bin/mark_pathway_www?@map00510/reference%3Dwhite/default%3D%23bfffbf/K03842/K00730/K07252) N-Glycan biosynthesis [PATH:ko00510] (3)

[00531](http://www.genome.jp/kegg-bin/mark_pathway_www?@map00531/reference%3Dwhite/default%3D%23bfffbf/K01217/K01137) Glycosaminoglycan degradation [PATH:ko00531] (2)

[00550](http://www.genome.jp/kegg-bin/mark_pathway_www?@map00550/reference%3Dwhite/default%3D%23bfffbf/K01000/K01915) Peptidoglycan biosynthesis [PATH:ko00550] (2)

[00600](http://www.genome.jp/kegg-bin/mark_pathway_www?@map00600/reference%3Dwhite/default%3D%23bfffbf/K00720) Glycosphingolipid metabolism [PATH:ko00600] [GO:0006665 0030148] (1)

**01190 Metabolism of Cofactors and Vitamins**

[00740](http://www.genome.jp/kegg-bin/mark_pathway_www?@map00740/reference%3Dwhite/default%3D%23bfffbf/K01497/K00953) Riboflavin metabolism [PATH:ko00740] (2)

[00750](http://www.genome.jp/kegg-bin/mark_pathway_www?@map00750/reference%3Dwhite/default%3D%23bfffbf/K00868) Vitamin B6 metabolism [PATH:ko00750] [GO:0008615] (1)

[00760](http://www.genome.jp/kegg-bin/mark_pathway_www?@map00760/reference%3Dwhite/default%3D%23bfffbf/K01463) Nicotinate and nicotinamide metabolism [PATH:ko00760] (1)

[00770](http://www.genome.jp/kegg-bin/mark_pathway_www?@map00770/reference%3Dwhite/default%3D%23bfffbf/K01463) Pantothenate and CoA biosynthesis [PATH:ko00770] (1)

[00780](http://www.genome.jp/kegg-bin/mark_pathway_www?@map00780/reference%3Dwhite/default%3D%23bfffbf/K00652/K01423) Biotin metabolism [PATH:ko00780] (2)

[00790](http://www.genome.jp/kegg-bin/mark_pathway_www?@map00790/reference%3Dwhite/default%3D%23bfffbf/K01529/K03637) Folate biosynthesis [PATH:ko00790] (2)

[00860](http://www.genome.jp/kegg-bin/mark_pathway_www?@map00860/reference%3Dwhite/default%3D%23bfffbf/K01885/K01698/K01599/K00228/K01764) Porphyrin and chlorophyll metabolism [PATH:ko00860] (5)

[00130](http://www.genome.jp/kegg-bin/mark_pathway_www?@map00130/reference%3Dwhite/default%3D%23bfffbf/K00334/K00336/K00338) Ubiquinone biosynthesis [PATH:ko00130] (3)

**01195 Biosynthesis of Secondary Metabolites**

[00900](http://www.genome.jp/kegg-bin/mark_pathway_www?@map00900/reference%3Dwhite/default%3D%23bfffbf/K00801/K00511) Terpenoid biosynthesis [PATH:ko00900] (2)

[00950](http://www.genome.jp/kegg-bin/mark_pathway_www?@map00950/reference%3Dwhite/default%3D%23bfffbf/K00813) Alkaloid biosynthesis I [PATH:ko00950] (1)

[00521](http://www.genome.jp/kegg-bin/mark_pathway_www?@map00521/reference%3Dwhite/default%3D%23bfffbf/K00844/K01835) Streptomycin biosynthesis [PATH:ko00521] (2)

[00401](http://www.genome.jp/kegg-bin/mark_pathway_www?@map00401/reference%3Dwhite/default%3D%23bfffbf/K00813) Novobiocin biosynthesis [PATH:ko00401] (1)

**01196 Xenobiotics Biodegradation and Metabolism**

[00930](http://www.genome.jp/kegg-bin/mark_pathway_www?@map00930/reference%3Dwhite/default%3D%23bfffbf/K01463) Caprolactam degradation [PATH:ko00930] (1)

[00632](http://www.genome.jp/kegg-bin/mark_pathway_www?@map00632/reference%3Dwhite/default%3D%23bfffbf/K00239) Benzoate degradation via CoA ligation [PATH:ko00632] (1)

[00624](http://www.genome.jp/kegg-bin/mark_pathway_www?@map00624/reference%3Dwhite/default%3D%23bfffbf/K00001) 1- and 2-Methylnaphthalene degradation [PATH:ko00624] (1)

[00980](http://www.genome.jp/kegg-bin/mark_pathway_www?@map00980/reference%3Dwhite/default%3D%23bfffbf/K00001) Metabolism of xenobiotics by cytochrome P450 [PATH:ko00980] (1)

**01210 Transcription**

[03020](http://www.genome.jp/kegg-bin/mark_pathway_www?@map03020/reference%3Dwhite/default%3D%23bfffbf/K03046/K03041/K03012/K03013/K03026/K03025/K03000) RNA polymerase [PATH:ko03020] (7)

[03022](http://www.genome.jp/kegg-bin/mark_pathway_www?@map03022/reference%3Dwhite/default%3D%23bfffbf/K03120/K03131/K03126/K03124/K03122/K03138/K03136/K03145) Basal transcription factors [PATH:ko03022] (8)

**01220 Translation**

[03010](http://www.genome.jp/kegg-bin/mark_pathway_www?@map03010/reference%3Dwhite/default%3D%23bfffbf/K02967/K02982/K02988/K02963/K02987/K02989/K02906/K02884/K02930/K02936/K02903/K02908/K02912/K02920/K02924/K02927/K02941) Ribosome [PATH:ko03010] [BR:ko03010] (17)

[00970](http://www.genome.jp/kegg-bin/mark_pathway_www?@map00970/reference%3Dwhite/default%3D%23bfffbf/K01874/K01883/K01885/K01875/K01881/K04567/K01880) Aminoacyl-tRNA biosynthesis [PATH:ko00970] (7)

**01230 Folding, Sorting and Degradation**

[03060](http://www.genome.jp/kegg-bin/mark_pathway_www?@map03060/reference%3Dwhite/default%3D%23bfffbf/K03076/K03106/K03110) Protein export [PATH:ko03060] (3)

[03090](http://www.genome.jp/kegg-bin/mark_pathway_www?@map03090/reference%3Dwhite/default%3D%23bfffbf/K02657/K01493) Type II secretion system [PATH:ko03090] (2)

[04120](http://www.genome.jp/kegg-bin/mark_pathway_www?@map04120/reference%3Dwhite/default%3D%23bfffbf/K03350/K03874/K03363/K03364/K03360) Ubiquitin mediated proteolysis [PATH:ko04120] (5)

[03050](http://www.genome.jp/kegg-bin/mark_pathway_www?@map03050/reference%3Dwhite/default%3D%23bfffbf/K02726/K02728/K03032/K03033/K03039/K03029/K03432) Proteasome [PATH:ko03050] [GO:0000502] (7)

**01240 Replication and Repair**

[03030](http://www.genome.jp/kegg-bin/mark_pathway_www?@map03030/reference%3Dwhite/default%3D%23bfffbf/K02325) DNA polymerase [PATH:ko03030] (1)

**01310 Membrane Transport**

[02010](http://www.genome.jp/kegg-bin/mark_pathway_www?@map02010/reference%3Dwhite/default%3D%23bfffbf/K01990/K02503/K05667) ABC transporters [PATH:ko02010] [BR:ko02000] [TC:3.A.1] (3)

**01320 Signal Transduction**

[02020](http://www.genome.jp/kegg-bin/mark_pathway_www?@map02020/reference%3Dwhite/default%3D%23bfffbf/K02657) Two-component system [PATH:ko02020] [GO:0000160] (1)

[04010dme](http://www.genome.jp/kegg-bin/mark_pathway_www?@map04010dme/reference%3Dwhite/default%3D%23bfffbf/K06268/K04438/K04441) MAPK signaling pathway [PATH:ko04010hsa] (3)

[04010hsa](http://www.genome.jp/kegg-bin/mark_pathway_www?@map04010hsa/reference%3Dwhite/default%3D%23bfffbf/K06268/K04438/K04441) MAPK signaling pathway [PATH:ko04010hsa] (3)

[04010sce](http://www.genome.jp/kegg-bin/mark_pathway_www?@map04010sce/reference%3Dwhite/default%3D%23bfffbf/K06268/K04438/K04441) MAPK signaling pathway [PATH:ko04010hsa] (3)

[04310](http://www.genome.jp/kegg-bin/mark_pathway_www?@map04310/reference%3Dwhite/default%3D%23bfffbf/K03456/K04354/K04508/K06268) Wnt signaling pathway [PATH:ko04310] [GO:0016055] (4)

[04330](http://www.genome.jp/kegg-bin/mark_pathway_www?@map04330/reference%3Dwhite/default%3D%23bfffbf/K06053/K06063/K06067) Notch signaling pathway [PATH:ko04330] [GO:0007219] (3)

[04340](http://www.genome.jp/kegg-bin/mark_pathway_www?@map04340/reference%3Dwhite/default%3D%23bfffbf/K06230/K02218) Hedgehog signaling pathway [PATH:ko04340] (2)

[04350](http://www.genome.jp/kegg-bin/mark_pathway_www?@map04350/reference%3Dwhite/default%3D%23bfffbf/K03456/K04354) TGF-beta signaling pathway [PATH:ko04350] [GO:0007179] (2)

[04370](http://www.genome.jp/kegg-bin/mark_pathway_www?@map04370/reference%3Dwhite/default%3D%23bfffbf/K06268/K04441) VEGF signaling pathway [PATH:ko04370] [GO:0048010] (2)

[04630](http://www.genome.jp/kegg-bin/mark_pathway_www?@map04630/reference%3Dwhite/default%3D%23bfffbf/K04705/K04707) Jak-STAT signaling pathway [PATH:ko04630] [GO:0007259] (2)

[04020](http://www.genome.jp/kegg-bin/mark_pathway_www?@map04020/reference%3Dwhite/default%3D%23bfffbf/K02183/K06268) Calcium signaling pathway [PATH:ko04020] [GO:0019722] (2)

[04070](http://www.genome.jp/kegg-bin/mark_pathway_www?@map04070/reference%3Dwhite/default%3D%23bfffbf/K00888/K00921/K02183) Phosphatidylinositol signaling system [PATH:ko04070] (3)

[04910](http://www.genome.jp/kegg-bin/mark_pathway_www?@map04910/reference%3Dwhite/default%3D%23bfffbf/K00688/K06269/K02183/K04707/K04438/K00850/K07199/K07204/K07207) Insulin signaling pathway [PATH:ko04910] (9)

[04920](http://www.genome.jp/kegg-bin/mark_pathway_www?@map04920/reference%3Dwhite/default%3D%23bfffbf/K07199) Adipocytokine signaling pathway [PATH:ko04920] (1)

[04150](http://www.genome.jp/kegg-bin/mark_pathway_www?@map04150/reference%3Dwhite/default%3D%23bfffbf/K07207/K08266/K07204/K03258) mTOR signaling pathway [PATH:ko04150] (4)

**01330 Signaling Molecules and Interaction**

[04514](http://www.genome.jp/kegg-bin/mark_pathway_www?@map04514/reference%3Dwhite/default%3D%23bfffbf/K06547) Cell adhesion molecules (CAMs) [PATH:ko04514] [BR:ko04514] [GO:0050839] (1)

**01410 Cell Motility**

[04810](http://www.genome.jp/kegg-bin/mark_pathway_www?@map04810/reference%3Dwhite/default%3D%23bfffbf/K04438/K06269/K00921/K05757/K05758/K05692/K05767/K05699) Regulation of actin cytoskeleton [PATH:ko04810] [GO:0030036] (8)

**01420 Cell Growth and Death**

[04110hsa](http://www.genome.jp/kegg-bin/mark_pathway_www?@map04110hsa/reference%3Dwhite/default%3D%23bfffbf/K06067/K05868/K06630/K03350/K03363/K03364/K06639/K02605/K02607/K02541/K02209/K02542/K02210/K03360/K02220/K06666/K02219/K06670/K06675/K06677) Cell cycle [PATH:ko04110hsa] (20)

[04110sce](http://www.genome.jp/kegg-bin/mark_pathway_www?@map04110sce/reference%3Dwhite/default%3D%23bfffbf/K06067/K05868/K06630/K03350/K03363/K03364/K06639/K02605/K02607/K02541/K02209/K02542/K02210/K03360/K02220/K06666/K02219/K06670/K06675/K06677) Cell cycle [PATH:ko04110hsa] (20)

[04210](http://www.genome.jp/kegg-bin/mark_pathway_www?@map04210/reference%3Dwhite/default%3D%23bfffbf/K06268) Apoptosis [PATH:ko04210] [GO:0006915] (1)

**01430 Cell Communication**

[04510](http://www.genome.jp/kegg-bin/mark_pathway_www?@map04510/reference%3Dwhite/default%3D%23bfffbf/K06269/K05692/K05699/K04438) Focal adhesion [PATH:ko04510] [GO:0005925] (4)

[04520](http://www.genome.jp/kegg-bin/mark_pathway_www?@map04520/reference%3Dwhite/default%3D%23bfffbf/K05699/K05692) Adherens junction [PATH:ko04520] [GO:0005912] (2)

[04530](http://www.genome.jp/kegg-bin/mark_pathway_www?@map04530/reference%3Dwhite/default%3D%23bfffbf/K03456/K04354/K06096/K05692/K06106/K05699) Tight junction [PATH:ko04530] [GO:0005923] (6)

[04540](http://www.genome.jp/kegg-bin/mark_pathway_www?@map04540/reference%3Dwhite/default%3D%23bfffbf/K02218) Gap junction [PATH:ko04540] [GO:0005921] (1)

**01460 Immune System**

[04640](http://www.genome.jp/kegg-bin/mark_pathway_www?@map04640/reference%3Dwhite/default%3D%23bfffbf/K01256) Hematopoietic cell lineage [PATH:ko04640] (1)

[04620](http://www.genome.jp/kegg-bin/mark_pathway_www?@map04620/reference%3Dwhite/default%3D%23bfffbf/K04441) Toll-like receptor signaling pathway [PATH:ko04620] (1)

[04650hsa](http://www.genome.jp/kegg-bin/mark_pathway_www?@map04650hsa/reference%3Dwhite/default%3D%23bfffbf/K06268) Natural killer cell mediated cytotoxicity [PATH:ko04650hsa] [GO:0042267] (1)

[04650mmu](http://www.genome.jp/kegg-bin/mark_pathway_www?@map04650mmu/reference%3Dwhite/default%3D%23bfffbf/K06268) Natural killer cell mediated cytotoxicity [PATH:ko04650hsa] [GO:0042267] (1)

[04612](http://www.genome.jp/kegg-bin/mark_pathway_www?@map04612/reference%3Dwhite/default%3D%23bfffbf/K04079) Antigen processing and presentation [PATH:ko04612] [GO:0019885 0019886 0042591] (1)

[04660](http://www.genome.jp/kegg-bin/mark_pathway_www?@map04660/reference%3Dwhite/default%3D%23bfffbf/K06268/K04707) T cell receptor signaling pathway [PATH:ko04660] (2)

[04662](http://www.genome.jp/kegg-bin/mark_pathway_www?@map04662/reference%3Dwhite/default%3D%23bfffbf/K06268) B cell receptor signaling pathway [PATH:ko04662] (1)

[04664](http://www.genome.jp/kegg-bin/mark_pathway_www?@map04664/reference%3Dwhite/default%3D%23bfffbf/K04441) Fc epsilon RI signaling pathway [PATH:ko04664] [GO:0045576] (1)

[04670](http://www.genome.jp/kegg-bin/mark_pathway_www?@map04670/reference%3Dwhite/default%3D%23bfffbf/K05692/K04441) Leukocyte transendothelial migration [PATH:ko04670] [GO:0050900] (2)

**01470 Nervous System**

[04720](http://www.genome.jp/kegg-bin/mark_pathway_www?@map04720/reference%3Dwhite/default%3D%23bfffbf/K06269/K06268/K02183) Long-term potentiation [PATH:ko04720] [GO:0048169] (3)

[04730](http://www.genome.jp/kegg-bin/mark_pathway_www?@map04730/reference%3Dwhite/default%3D%23bfffbf/K03456/K04354) Long-term depression [PATH:ko04730] [GO:0048169] (2)

**01440 Development**

[04360](http://www.genome.jp/kegg-bin/mark_pathway_www?@map04360/reference%3Dwhite/default%3D%23bfffbf/K06268) Axon guidance [PATH:ko04360] [GO:0007411] (1)

**01450 Behavior**

[04710ath](http://www.genome.jp/kegg-bin/mark_pathway_www?@map04710ath/reference%3Dwhite/default%3D%23bfffbf/K02218) Circadian rhythm [PATH:ko04710dme] (1)

[04710dme](http://www.genome.jp/kegg-bin/mark_pathway_www?@map04710dme/reference%3Dwhite/default%3D%23bfffbf/K02218) Circadian rhythm [PATH:ko04710dme] (1)

[04710m](http://www.genome.jp/kegg-bin/mark_pathway_www?@map04710m/reference%3Dwhite/default%3D%23bfffbf/K02218) Circadian rhythm [PATH:ko04710dme] (1)

**01510 Neurodegenerative Disorders**

[05010](http://www.genome.jp/kegg-bin/mark_pathway_www?@map05010/reference%3Dwhite/default%3D%23bfffbf/K04524/K01408) Alzheimer's disease [PATH:ko05010] (2)

[05030](http://www.genome.jp/kegg-bin/mark_pathway_www?@map05030/reference%3Dwhite/default%3D%23bfffbf/K04567) Amyotrophic lateral sclerosis (ALS) [PATH:ko05030] (1)

[05040](http://www.genome.jp/kegg-bin/mark_pathway_www?@map05040/reference%3Dwhite/default%3D%23bfffbf/K04648/K01697/K02183) Huntington's disease [PATH:ko05040] (3)

[05060](http://www.genome.jp/kegg-bin/mark_pathway_www?@map05060/reference%3Dwhite/default%3D%23bfffbf/K04077) Prion disease [PATH:ko05060] (1)

**01530 Metabolic Disorders**

[04940](http://www.genome.jp/kegg-bin/mark_pathway_www?@map04940/reference%3Dwhite/default%3D%23bfffbf/K04077) Type I diabetes mellitus [PATH:ko04940] (1)
