# Supplementary material for: Transcriptome of Pneumocystis carinii during Fulminate Infection: Carbohydrate Metabolism and the Concept of a Compatible Parasite
Source: PLoS One. 2007 May 9;2(5):e423. doi: 10.1371/journal.pone.0000423 (PMC1855432; doi:10.1371/journal.pone.0000423)
Supplement: Table S5 — Orthology List for Unigenes in KEGG pathways. (0.24 MB DOC) [file pone.0000423.s008.doc]

# KEGG Orthology (KO)

**[**[**KO list**](http://www.genome.jp/kegg-bin/kaas_main?mode=result&id=1149190105&mail=beslaven@yahoo.com)**]**

**[KO hierarchy]**

**[**[**Pathway map**](http://www.genome.jp/kegg-bin/kaas_main?mode=map&id=1149190105&mail=beslaven@yahoo.com)**]**

**[** [**Threshold change**](http://www.genome.jp/kegg-bin/kaas_main?mode=threshold&id=1149190105&mail=beslaven@yahoo.com) **]**

**[**[**Download**](http://www.genome.jp/kegg-bin/kaas_main?mode=dl&id=1149190105&mail=beslaven@yahoo.com)**]**

**01110 Carbohydrate Metabolism**

00010 Glycolysis / Gluconeogenesis [PATH:ko00010]

[K00844](http://www.genome.jp/dbget-bin/www_bget?ko+K00844) E2.7.1.1; hexokinase [EC:2.7.1.1]

cDNAv1_0.fasta.screen.Contig604

[K00850](http://www.genome.jp/dbget-bin/www_bget?ko+K00850) E2.7.1.11, pfk; 6-phosphofructokinase [EC:2.7.1.11]

14e02uni.f.ab1

14e02uni.t.ab1

[K00927](http://www.genome.jp/dbget-bin/www_bget?ko+K00927) E2.7.2.3, pgk; phosphoglycerate kinase [EC:2.7.2.3]

cDNAv1_0.fasta.screen.Contig611

[K01834](http://www.genome.jp/dbget-bin/www_bget?ko+K01834) E5.4.2.1, gpm; phosphoglycerate mutase [EC:5.4.2.1]

cDNAv1_0.fasta.screen.Contig993

[K00162](http://www.genome.jp/dbget-bin/www_bget?ko+K00162) E1.2.4.1B, pdhB; pyruvate dehydrogenase E1 component, beta subunit [EC:1.2.4.1]

cDNAv1_0.fasta.screen.Contig55

[K00627](http://www.genome.jp/dbget-bin/www_bget?ko+K00627) E2.3.1.12, pdhC; pyruvate dehydrogenase E2 component (dihydrolipoamide acetyltransferase) [EC:2.3.1.12]

3h10uni.f.ab1

[K00001](http://www.genome.jp/dbget-bin/www_bget?ko+K00001) E1.1.1.1, adh; alcohol dehydrogenase [EC:1.1.1.1]

cDNAv1_0.fasta.screen.Contig685

[K01835](http://www.genome.jp/dbget-bin/www_bget?ko+K01835) E5.4.2.2, pgm; phosphoglucomutase [EC:5.4.2.2]

cDNAv1_0.fasta.screen.Contig398

00020 Citrate cycle (TCA cycle) [PATH:ko00020]

[K01647](http://www.genome.jp/dbget-bin/www_bget?ko+K01647) E2.3.3.1, gltA; citrate synthase [EC:2.3.3.1]

cDNAv1_0.fasta.screen.Contig961

[K01648](http://www.genome.jp/dbget-bin/www_bget?ko+K01648) E2.3.3.8, ACLY; ATP citrate (pro-S)-lyase [EC:2.3.3.8]

cDNAv1_0.fasta.screen.Contig749

cDNAv1_0.fasta.screen.Contig808

[K01681](http://www.genome.jp/dbget-bin/www_bget?ko+K01681) E4.2.1.3A, acnA; aconitate hydratase 1 [EC:4.2.1.3]

cDNAv1_0.fasta.screen.Contig547

15h06uni.e.ab1

[K00164](http://www.genome.jp/dbget-bin/www_bget?ko+K00164) E1.2.4.2, sucA; 2-oxoglutarate dehydrogenase E1 component [EC:1.2.4.2]

1h12uni.t.ab1

[K00239](http://www.genome.jp/dbget-bin/www_bget?ko+K00239) SDHA; succinate dehydrogenase flavoprotein subunit [EC:1.3.99.1]

cDNAv1_0.fasta.screen.Contig722

[K00026](http://www.genome.jp/dbget-bin/www_bget?ko+K00026) E1.1.1.37B, mdh; malate dehydrogenase [EC:1.1.1.37]

cDNAv1_0.fasta.screen.Contig186

cDNAv1_0.fasta.screen.Contig846

00030 Pentose phosphate pathway [PATH:ko00030]

[K00033](http://www.genome.jp/dbget-bin/www_bget?ko+K00033) E1.1.1.44, gnd; 6-phosphogluconate dehydrogenase [EC:1.1.1.44]

cDNAv1_0.fasta.screen.Contig940

[K01783](http://www.genome.jp/dbget-bin/www_bget?ko+K01783) E5.1.3.1, rpe; ribulose-phosphate 3-epimerase [EC:5.1.3.1]

cDNAv1_0.fasta.screen.Contig849

[K00850](http://www.genome.jp/dbget-bin/www_bget?ko+K00850) E2.7.1.11, pfk; 6-phosphofructokinase [EC:2.7.1.11]

14e02uni.f.ab1

14e02uni.t.ab1

[K01835](http://www.genome.jp/dbget-bin/www_bget?ko+K01835) E5.4.2.2, pgm; phosphoglucomutase [EC:5.4.2.2]

cDNAv1_0.fasta.screen.Contig398

[K00948](http://www.genome.jp/dbget-bin/www_bget?ko+K00948) E2.7.6.1, prsA; ribose-phosphate pyrophosphokinase [EC:2.7.6.1]

cDNAv1_0.fasta.screen.Contig616

00040 Pentose and glucuronate interconversions [PATH:ko00040]

[K00963](http://www.genome.jp/dbget-bin/www_bget?ko+K00963) E2.7.7.9, galU; UTP--glucose-1-phosphate uridylyltransferase [EC:2.7.7.9]

cDNAv1_0.fasta.screen.Contig696

[K01783](http://www.genome.jp/dbget-bin/www_bget?ko+K01783) E5.1.3.1, rpe; ribulose-phosphate 3-epimerase [EC:5.1.3.1]

cDNAv1_0.fasta.screen.Contig849

00051 Fructose and mannose metabolism [PATH:ko00051]

[K00966](http://www.genome.jp/dbget-bin/www_bget?ko+K00966) E2.7.7.13; mannose-1-phosphate guanylyltransferase [EC:2.7.7.13]

cDNAv1_0.fasta.screen.Contig904

[K00844](http://www.genome.jp/dbget-bin/www_bget?ko+K00844) E2.7.1.1; hexokinase [EC:2.7.1.1]

cDNAv1_0.fasta.screen.Contig604

[K00850](http://www.genome.jp/dbget-bin/www_bget?ko+K00850) E2.7.1.11, pfk; 6-phosphofructokinase [EC:2.7.1.11]

14e02uni.f.ab1

14e02uni.t.ab1

[K00900](http://www.genome.jp/dbget-bin/www_bget?ko+K00900) E2.7.1.105, PFK; 6-phosphofructo-2-kinase [EC:2.7.1.105]

cDNAv1_0.fasta.screen.Contig831

00052 Galactose metabolism [PATH:ko00052]

[K00963](http://www.genome.jp/dbget-bin/www_bget?ko+K00963) E2.7.7.9, galU; UTP--glucose-1-phosphate uridylyltransferase [EC:2.7.7.9]

cDNAv1_0.fasta.screen.Contig696

[K01835](http://www.genome.jp/dbget-bin/www_bget?ko+K01835) E5.4.2.2, pgm; phosphoglucomutase [EC:5.4.2.2]

cDNAv1_0.fasta.screen.Contig398

[K00844](http://www.genome.jp/dbget-bin/www_bget?ko+K00844) E2.7.1.1; hexokinase [EC:2.7.1.1]

cDNAv1_0.fasta.screen.Contig604

[K00850](http://www.genome.jp/dbget-bin/www_bget?ko+K00850) E2.7.1.11, pfk; 6-phosphofructokinase [EC:2.7.1.11]

14e02uni.f.ab1

14e02uni.t.ab1

00500 Starch and sucrose metabolism [PATH:ko00500]

[K00963](http://www.genome.jp/dbget-bin/www_bget?ko+K00963) E2.7.7.9, galU; UTP--glucose-1-phosphate uridylyltransferase [EC:2.7.7.9]

cDNAv1_0.fasta.screen.Contig696

[K01835](http://www.genome.jp/dbget-bin/www_bget?ko+K01835) E5.4.2.2, pgm; phosphoglucomutase [EC:5.4.2.2]

cDNAv1_0.fasta.screen.Contig398

[K00844](http://www.genome.jp/dbget-bin/www_bget?ko+K00844) E2.7.1.1; hexokinase [EC:2.7.1.1]

cDNAv1_0.fasta.screen.Contig604

[K00688](http://www.genome.jp/dbget-bin/www_bget?ko+K00688) E2.4.1.1, glgP, PYG; starch phosphorylase [EC:2.4.1.1]

cDNAv1_0.fasta.screen.Contig133

[K01529](http://www.genome.jp/dbget-bin/www_bget?ko+K01529) E3.6.1.-; [EC:3.6.1.-]

cDNAv1_0.fasta.screen.Contig659

cDNAv1_0.fasta.screen.Contig818

cDNAv1_0.fasta.screen.Contig879

00530 Aminosugars metabolism [PATH:ko00530]

[K00820](http://www.genome.jp/dbget-bin/www_bget?ko+K00820) E2.6.1.16, glmS; glucosamine--fructose-6-phosphate aminotransferase (isomerizing) [EC:2.6.1.16]

cDNAv1_0.fasta.screen.Contig275

[K00844](http://www.genome.jp/dbget-bin/www_bget?ko+K00844) E2.7.1.1; hexokinase [EC:2.7.1.1]

cDNAv1_0.fasta.screen.Contig604

00520 Nucleotide sugars metabolism [PATH:ko00520]

[K00963](http://www.genome.jp/dbget-bin/www_bget?ko+K00963) E2.7.7.9, galU; UTP--glucose-1-phosphate uridylyltransferase [EC:2.7.7.9]

cDNAv1_0.fasta.screen.Contig696

00620 Pyruvate metabolism [PATH:ko00620]

[K00162](http://www.genome.jp/dbget-bin/www_bget?ko+K00162) E1.2.4.1B, pdhB; pyruvate dehydrogenase E1 component, beta subunit [EC:1.2.4.1]

cDNAv1_0.fasta.screen.Contig55

[K00627](http://www.genome.jp/dbget-bin/www_bget?ko+K00627) E2.3.1.12, pdhC; pyruvate dehydrogenase E2 component (dihydrolipoamide acetyltransferase) [EC:2.3.1.12]

3h10uni.f.ab1

[K00026](http://www.genome.jp/dbget-bin/www_bget?ko+K00026) E1.1.1.37B, mdh; malate dehydrogenase [EC:1.1.1.37]

cDNAv1_0.fasta.screen.Contig186

cDNAv1_0.fasta.screen.Contig846

00630 Glyoxylate and dicarboxylate metabolism [PATH:ko00630]

[K00026](http://www.genome.jp/dbget-bin/www_bget?ko+K00026) E1.1.1.37B, mdh; malate dehydrogenase [EC:1.1.1.37]

cDNAv1_0.fasta.screen.Contig186

cDNAv1_0.fasta.screen.Contig846

[K01647](http://www.genome.jp/dbget-bin/www_bget?ko+K01647) E2.3.3.1, gltA; citrate synthase [EC:2.3.3.1]

cDNAv1_0.fasta.screen.Contig961

[K01681](http://www.genome.jp/dbget-bin/www_bget?ko+K01681) E4.2.1.3A, acnA; aconitate hydratase 1 [EC:4.2.1.3]

cDNAv1_0.fasta.screen.Contig547

15h06uni.e.ab1

00650 Butanoate metabolism [PATH:ko00650]

[K00239](http://www.genome.jp/dbget-bin/www_bget?ko+K00239) SDHA; succinate dehydrogenase flavoprotein subunit [EC:1.3.99.1]

cDNAv1_0.fasta.screen.Contig722

[K01641](http://www.genome.jp/dbget-bin/www_bget?ko+K01641) E2.3.3.10, pksG; hydroxymethylglutaryl-CoA synthase [EC:2.3.3.10]

cDNAv1_0.fasta.screen.Contig768

[K00162](http://www.genome.jp/dbget-bin/www_bget?ko+K00162) E1.2.4.1B, pdhB; pyruvate dehydrogenase E1 component, beta subunit [EC:1.2.4.1]

cDNAv1_0.fasta.screen.Contig55

00562 Inositol phosphate metabolism [PATH:ko00562]

[K00888](http://www.genome.jp/dbget-bin/www_bget?ko+K00888) E2.7.1.67; phosphatidylinositol 4-kinase [EC:2.7.1.67]

cDNAv1_0.fasta.screen.Contig791

[K00924](http://www.genome.jp/dbget-bin/www_bget?ko+K00924) E2.7.1.-; [EC:2.7.1.-]

cDNAv1_0.fasta.screen.Contig95

2e10uni.e.ab1

**01120 Energy Metabolism**

00190 Oxidative phosphorylation [PATH:ko00190]

[K00334](http://www.genome.jp/dbget-bin/www_bget?ko+K00334) NUOE; NADH dehydrogenase I chain E [EC:1.6.5.3]

cDNAv1_0.fasta.screen.Contig537

[K00336](http://www.genome.jp/dbget-bin/www_bget?ko+K00336) NUOG; NADH dehydrogenase I chain G [EC:1.6.5.3]

cDNAv1_0.fasta.screen.Contig180

[K00338](http://www.genome.jp/dbget-bin/www_bget?ko+K00338) NUOI; NADH dehydrogenase I chain I [EC:1.6.5.3]

cDNAv1_0.fasta.screen.Contig268

[K03937](http://www.genome.jp/dbget-bin/www_bget?ko+K03937) NDUFS4; NADH dehydrogenase (ubiquinone) Fe-S protein 4 [EC:1.6.5.3 1.6.99.3]

cDNAv1_0.fasta.screen.Contig142

[K03885](http://www.genome.jp/dbget-bin/www_bget?ko+K03885) NDH; NADH dehydrogenase [EC:1.6.99.3]

cDNAv1_0.fasta.screen.Contig118

[K00239](http://www.genome.jp/dbget-bin/www_bget?ko+K00239) SDHA; succinate dehydrogenase flavoprotein subunit [EC:1.3.99.1]

cDNAv1_0.fasta.screen.Contig722

[K00415](http://www.genome.jp/dbget-bin/www_bget?ko+K00415) QCR2; ubiquinol-cytochrome c reductase core subunit 2 [EC:1.10.2.2]

cDNAv1_0.fasta.screen.Contig1030

[K02301](http://www.genome.jp/dbget-bin/www_bget?ko+K02301) CYOE; protoheme IX farnesyltransferase [EC:2.5.1.-]

cDNAv1_0.fasta.screen.Contig718

[K02258](http://www.genome.jp/dbget-bin/www_bget?ko+K02258) COX11; cytochrome c oxidase subunit XI assembly protein

cDNAv1_0.fasta.screen.Contig347

[K01507](http://www.genome.jp/dbget-bin/www_bget?ko+K01507) E3.6.1.1, ppa; inorganic pyrophosphatase [EC:3.6.1.1]

cDNAv1_0.fasta.screen.Contig502

00193 ATP synthesis [PATH:ko00193] [TC:3.A.2]

[K02112](http://www.genome.jp/dbget-bin/www_bget?ko+K02112) ATPF1B, atpD; F-type H+-transporting ATPase beta chain [EC:3.6.3.14] [TC:3.A.2.1]

11a11uni.e.ab1

[K02117](http://www.genome.jp/dbget-bin/www_bget?ko+K02117) ATPVA, ntpA; V-type H+-transporting ATPase subunit A [EC:3.6.3.14] [TC:3.A.2.2]

cDNAv1_0.fasta.screen.Contig693

[K02154](http://www.genome.jp/dbget-bin/www_bget?ko+K02154) ATPeVI, ATP6N1A; V-type H+-transporting ATPase subunit I [EC:3.6.3.14] [TC:3.A.2.2]

cDNAv1_0.fasta.screen.Contig885

[K02149](http://www.genome.jp/dbget-bin/www_bget?ko+K02149) ATPeVD, ATP6M; V-type H+-transporting ATPase subunit D [EC:3.6.3.14] [TC:3.A.2.2]

cDNAv1_0.fasta.screen.Contig876

[K02144](http://www.genome.jp/dbget-bin/www_bget?ko+K02144) ATPeV54kD; V-type H+-transporting ATPase 54 kD subunit [EC:3.6.3.14] [TC:3.A.2.2]

cDNAv1_0.fasta.screen.Contig814

00710 Carbon fixation [PATH:ko00710]

[K00927](http://www.genome.jp/dbget-bin/www_bget?ko+K00927) E2.7.2.3, pgk; phosphoglycerate kinase [EC:2.7.2.3]

cDNAv1_0.fasta.screen.Contig611

[K01783](http://www.genome.jp/dbget-bin/www_bget?ko+K01783) E5.1.3.1, rpe; ribulose-phosphate 3-epimerase [EC:5.1.3.1]

cDNAv1_0.fasta.screen.Contig849

[K00813](http://www.genome.jp/dbget-bin/www_bget?ko+K00813) E2.6.1.1B, aspC; aspartate aminotransferase [EC:2.6.1.1]

cDNAv1_0.fasta.screen.Contig702

[K00026](http://www.genome.jp/dbget-bin/www_bget?ko+K00026) E1.1.1.37B, mdh; malate dehydrogenase [EC:1.1.1.37]

cDNAv1_0.fasta.screen.Contig186

cDNAv1_0.fasta.screen.Contig846

00720 Reductive carboxylate cycle (CO2 fixation) [PATH:ko00720]

[K00026](http://www.genome.jp/dbget-bin/www_bget?ko+K00026) E1.1.1.37B, mdh; malate dehydrogenase [EC:1.1.1.37]

cDNAv1_0.fasta.screen.Contig186

cDNAv1_0.fasta.screen.Contig846

[K00239](http://www.genome.jp/dbget-bin/www_bget?ko+K00239) SDHA; succinate dehydrogenase flavoprotein subunit [EC:1.3.99.1]

cDNAv1_0.fasta.screen.Contig722

[K01681](http://www.genome.jp/dbget-bin/www_bget?ko+K01681) E4.2.1.3A, acnA; aconitate hydratase 1 [EC:4.2.1.3]

cDNAv1_0.fasta.screen.Contig547

15h06uni.e.ab1

00680 Methane metabolism [PATH:ko00680]

[K03782](http://www.genome.jp/dbget-bin/www_bget?ko+K03782) KATG; catalase/peroxidase [EC:1.11.1.6]

cDNAv1_0.fasta.screen.Contig327

00910 Nitrogen metabolism [PATH:ko00910]

[K01915](http://www.genome.jp/dbget-bin/www_bget?ko+K01915) E6.3.1.2, glnA; glutamine synthetase [EC:6.3.1.2]

cDNAv1_0.fasta.screen.Contig173

00920 Sulfur metabolism [PATH:ko00920]

[K01738](http://www.genome.jp/dbget-bin/www_bget?ko+K01738) E2.5.1.47, cysK; cysteine synthase [EC:2.5.1.47]

cDNAv1_0.fasta.screen.Contig932

[K01537](http://www.genome.jp/dbget-bin/www_bget?ko+K01537) E3.6.3.8; Ca2+-transporting ATPase [EC:3.6.3.8] [TC:3.A.3.2]

5f08uni.e.ab1

[K05853](http://www.genome.jp/dbget-bin/www_bget?ko+K05853) ATP2A; Ca2+ transporting ATPase, sarcoplasmic/endoplasmic reticulum [EC:3.6.3.8] [TC:3.A.3.2]

cDNAv1_0.fasta.screen.Contig217

5b07uni.e.ab1

**01130 Lipid Metabolism**

00061 Fatty acid biosynthesis [PATH:ko00061]

[K01946](http://www.genome.jp/dbget-bin/www_bget?ko+K01946) E6.3.4.14, accC; biotin carboxylase [EC:6.3.4.14]

cDNAv1_0.fasta.screen.Contig90

[K00647](http://www.genome.jp/dbget-bin/www_bget?ko+K00647) E2.3.1.41A, fabB, fabF; 3-oxoacyl-[acyl-carrier-protein] synthase I/II [EC:2.3.1.41]

cDNAv1_0.fasta.screen.Contig238

00071 Fatty acid metabolism [PATH:ko00071]

[K00001](http://www.genome.jp/dbget-bin/www_bget?ko+K00001) E1.1.1.1, adh; alcohol dehydrogenase [EC:1.1.1.1]

cDNAv1_0.fasta.screen.Contig685

00072 Synthesis and degradation of ketone bodies [PATH:ko00072]

[K01641](http://www.genome.jp/dbget-bin/www_bget?ko+K01641) E2.3.3.10, pksG; hydroxymethylglutaryl-CoA synthase [EC:2.3.3.10]

cDNAv1_0.fasta.screen.Contig768

00100 Biosynthesis of steroids [PATH:ko00100]

[K00801](http://www.genome.jp/dbget-bin/www_bget?ko+K00801) E2.5.1.21, FDFT1; farnesyl-diphosphate farnesyltransferase [EC:2.5.1.21]

cDNAv1_0.fasta.screen.Contig911

[K00511](http://www.genome.jp/dbget-bin/www_bget?ko+K00511) E1.14.99.7, SQLE; squalene monooxygenase [EC:1.14.99.7]

cDNAv1_0.fasta.screen.Contig970

00120 Bile acid biosynthesis [PATH:ko00120]

[K00001](http://www.genome.jp/dbget-bin/www_bget?ko+K00001) E1.1.1.1, adh; alcohol dehydrogenase [EC:1.1.1.1]

cDNAv1_0.fasta.screen.Contig685

00561 Glycerolipid metabolism [PATH:ko00561]

[K00001](http://www.genome.jp/dbget-bin/www_bget?ko+K00001) E1.1.1.1, adh; alcohol dehydrogenase [EC:1.1.1.1]

cDNAv1_0.fasta.screen.Contig685

[K00864](http://www.genome.jp/dbget-bin/www_bget?ko+K00864) E2.7.1.30, glpK; glycerol kinase [EC:2.7.1.30]

cDNAv1_0.fasta.screen.Contig1011

[K01046](http://www.genome.jp/dbget-bin/www_bget?ko+K01046) E3.1.1.3; triacylglycerol lipase [EC:3.1.1.3]

3f08uni.e.ab1

00564 Glycerophospholipid metabolism [PATH:ko00564]

[K00111](http://www.genome.jp/dbget-bin/www_bget?ko+K00111) E1.1.99.5A, glpA, glpD; glycerol-3-phosphate dehydrogenase [EC:1.1.99.5]

cDNAv1_0.fasta.screen.Contig840

[K00998](http://www.genome.jp/dbget-bin/www_bget?ko+K00998) E2.7.8.8, pssA; phosphatidylserine synthase [EC:2.7.8.8]

17d02uni.t.ab1

[K01613](http://www.genome.jp/dbget-bin/www_bget?ko+K01613) E4.1.1.65, psd; phosphatidylserine decarboxylase [EC:4.1.1.65]

cDNAv1_0.fasta.screen.Contig725

[K01062](http://www.genome.jp/dbget-bin/www_bget?ko+K01062) E3.1.1.47, PAFAH; 1-alkyl-2-acetylglycerophosphocholine esterase [EC:3.1.1.47]

cDNAv1_0.fasta.screen.Contig766

**01140 Nucleotide Metabolism**

00230 Purine metabolism [PATH:ko00230]

[K00948](http://www.genome.jp/dbget-bin/www_bget?ko+K00948) E2.7.6.1, prsA; ribose-phosphate pyrophosphokinase [EC:2.7.6.1]

cDNAv1_0.fasta.screen.Contig616

[K01588](http://www.genome.jp/dbget-bin/www_bget?ko+K01588) E4.1.1.21A, purE; phosphoribosylaminoimidazole carboxylase catalytic subunit [EC:4.1.1.21]

cDNAv1_0.fasta.screen.Contig984

[K00088](http://www.genome.jp/dbget-bin/www_bget?ko+K00088) E1.1.1.205, guaB; IMP dehydrogenase [EC:1.1.1.205]

cDNAv1_0.fasta.screen.Contig110

[K01951](http://www.genome.jp/dbget-bin/www_bget?ko+K01951) E6.3.5.2, guaA; GMP synthase (glutamine-hydrolysing) [EC:6.3.5.2]

cDNAv1_0.fasta.screen.Contig661

[K00525](http://www.genome.jp/dbget-bin/www_bget?ko+K00525) E1.17.4.1A, nrdA, nrdE; ribonucleoside-diphosphate reductase alpha chain [EC:1.17.4.1]

4c11uni.f.ab1

[K00856](http://www.genome.jp/dbget-bin/www_bget?ko+K00856) E2.7.1.20, ADK; adenosine kinase [EC:2.7.1.20]

cDNAv1_0.fasta.screen.Contig121

[K00939](http://www.genome.jp/dbget-bin/www_bget?ko+K00939) E2.7.4.3, adk; adenylate kinase [EC:2.7.4.3]

cDNAv1_0.fasta.screen.Contig194

cDNAv1_0.fasta.screen.Contig201

[K01509](http://www.genome.jp/dbget-bin/www_bget?ko+K01509) E3.6.1.3; adenosinetriphosphatase [EC:3.6.1.3]

cDNAv1_0.fasta.screen.Contig367

cDNAv1_0.fasta.screen.Contig368

cDNAv1_0.fasta.screen.Contig736

[K01553](http://www.genome.jp/dbget-bin/www_bget?ko+K01553) E3.6.4.1; myosin ATPase [EC:3.6.4.1]

cDNAv1_0.fasta.screen.Contig588

[K01939](http://www.genome.jp/dbget-bin/www_bget?ko+K01939) E6.3.4.4, purA; adenylosuccinate synthase [EC:6.3.4.4]

cDNAv1_0.fasta.screen.Contig218

00240 Pyrimidine metabolism [PATH:ko00240]

[K00609](http://www.genome.jp/dbget-bin/www_bget?ko+K00609) E2.1.3.2C, pyrB; aspartate carbamoyltransferase catalytic chain [EC:2.1.3.2]

cDNAv1_0.fasta.screen.Contig681

[K00384](http://www.genome.jp/dbget-bin/www_bget?ko+K00384) E1.8.1.9, trxB; thioredoxin reductase (NADPH) [EC:1.8.1.9]

cDNAv1_0.fasta.screen.Contig166

[K00525](http://www.genome.jp/dbget-bin/www_bget?ko+K00525) E1.17.4.1A, nrdA, nrdE; ribonucleoside-diphosphate reductase alpha chain [EC:1.17.4.1]

4c11uni.f.ab1

[K01493](http://www.genome.jp/dbget-bin/www_bget?ko+K01493) E3.5.4.12, comEB; dCMP deaminase [EC:3.5.4.12]

cDNAv1_0.fasta.screen.Contig120

**01150 Amino Acid Metabolism**

00251 Glutamate metabolism [PATH:ko00251]

[K00813](http://www.genome.jp/dbget-bin/www_bget?ko+K00813) E2.6.1.1B, aspC; aspartate aminotransferase [EC:2.6.1.1]

cDNAv1_0.fasta.screen.Contig702

[K01885](http://www.genome.jp/dbget-bin/www_bget?ko+K01885) E6.1.1.17, gltX; glutamyl-tRNA synthetase [EC:6.1.1.17]

cDNAv1_0.fasta.screen.Contig880

[K00820](http://www.genome.jp/dbget-bin/www_bget?ko+K00820) E2.6.1.16, glmS; glucosamine--fructose-6-phosphate aminotransferase (isomerizing) [EC:2.6.1.16]

cDNAv1_0.fasta.screen.Contig275

[K01915](http://www.genome.jp/dbget-bin/www_bget?ko+K01915) E6.3.1.2, glnA; glutamine synthetase [EC:6.3.1.2]

cDNAv1_0.fasta.screen.Contig173

[K01920](http://www.genome.jp/dbget-bin/www_bget?ko+K01920) E6.3.2.3, gshB; glutathione synthase [EC:6.3.2.3]

cDNAv1_0.fasta.screen.Contig315

[K01950](http://www.genome.jp/dbget-bin/www_bget?ko+K01950) E6.3.5.1, nadE; NAD+ synthase (glutamine-hydrolysing) [EC:6.3.5.1]

cDNAv1_0.fasta.screen.Contig855

[K01951](http://www.genome.jp/dbget-bin/www_bget?ko+K01951) E6.3.5.2, guaA; GMP synthase (glutamine-hydrolysing) [EC:6.3.5.2]

cDNAv1_0.fasta.screen.Contig661

00252 Alanine and aspartate metabolism [PATH:ko00252]

[K00813](http://www.genome.jp/dbget-bin/www_bget?ko+K00813) E2.6.1.1B, aspC; aspartate aminotransferase [EC:2.6.1.1]

cDNAv1_0.fasta.screen.Contig702

[K00609](http://www.genome.jp/dbget-bin/www_bget?ko+K00609) E2.1.3.2C, pyrB; aspartate carbamoyltransferase catalytic chain [EC:2.1.3.2]

cDNAv1_0.fasta.screen.Contig681

[K01939](http://www.genome.jp/dbget-bin/www_bget?ko+K01939) E6.3.4.4, purA; adenylosuccinate synthase [EC:6.3.4.4]

cDNAv1_0.fasta.screen.Contig218

00260 Glycine, serine and threonine metabolism [PATH:ko00260]

[K01880](http://www.genome.jp/dbget-bin/www_bget?ko+K01880) E6.1.1.14C, GRS1; glycyl-tRNA synthetase, class II [EC:6.1.1.14]

12f12uni.e.ab1

[K00998](http://www.genome.jp/dbget-bin/www_bget?ko+K00998) E2.7.8.8, pssA; phosphatidylserine synthase [EC:2.7.8.8]

17d02uni.t.ab1

[K01613](http://www.genome.jp/dbget-bin/www_bget?ko+K01613) E4.1.1.65, psd; phosphatidylserine decarboxylase [EC:4.1.1.65]

cDNAv1_0.fasta.screen.Contig725

[K01875](http://www.genome.jp/dbget-bin/www_bget?ko+K01875) E6.1.1.11, serS; seryl-tRNA synthetase [EC:6.1.1.11]

cDNAv1_0.fasta.screen.Contig989

00271 Methionine metabolism [PATH:ko00271]

[K01874](http://www.genome.jp/dbget-bin/www_bget?ko+K01874) E6.1.1.10, metG; methionyl-tRNA synthetase [EC:6.1.1.10]

cDNAv1_0.fasta.screen.Contig635

00272 Cysteine metabolism [PATH:ko00272]

[K01883](http://www.genome.jp/dbget-bin/www_bget?ko+K01883) E6.1.1.16A, cysS; cysteinyl-tRNA synthetase [EC:6.1.1.16]

cDNAv1_0.fasta.screen.Contig203

cDNAv1_0.fasta.screen.Contig509

[K01738](http://www.genome.jp/dbget-bin/www_bget?ko+K01738) E2.5.1.47, cysK; cysteine synthase [EC:2.5.1.47]

cDNAv1_0.fasta.screen.Contig932

[K00813](http://www.genome.jp/dbget-bin/www_bget?ko+K00813) E2.6.1.1B, aspC; aspartate aminotransferase [EC:2.6.1.1]

cDNAv1_0.fasta.screen.Contig702

00280 Valine, leucine and isoleucine degradation [PATH:ko00280]

[K01641](http://www.genome.jp/dbget-bin/www_bget?ko+K01641) E2.3.3.10, pksG; hydroxymethylglutaryl-CoA synthase [EC:2.3.3.10]

cDNAv1_0.fasta.screen.Contig768

00290 Valine, leucine and isoleucine biosynthesis [PATH:ko00290]

[K00162](http://www.genome.jp/dbget-bin/www_bget?ko+K00162) E1.2.4.1B, pdhB; pyruvate dehydrogenase E1 component, beta subunit [EC:1.2.4.1]

cDNAv1_0.fasta.screen.Contig55

00300 Lysine biosynthesis [PATH:ko00300]

[K04567](http://www.genome.jp/dbget-bin/www_bget?ko+K04567) LYSU, KARS; lysyl-tRNA synthetase, class II [EC:6.1.1.6]

cDNAv1_0.fasta.screen.Contig790

cDNAv1_0.fasta.screen.Contig1047

00310 Lysine degradation [PATH:ko00310]

[K00164](http://www.genome.jp/dbget-bin/www_bget?ko+K00164) E1.2.4.2, sucA; 2-oxoglutarate dehydrogenase E1 component [EC:1.2.4.2]

1h12uni.t.ab1

[K01423](http://www.genome.jp/dbget-bin/www_bget?ko+K01423) E3.4.-.-; [EC:3.4.-.-]

cDNAv1_0.fasta.screen.Contig187

00330 Arginine and proline metabolism [PATH:ko00330]

[K00813](http://www.genome.jp/dbget-bin/www_bget?ko+K00813) E2.6.1.1B, aspC; aspartate aminotransferase [EC:2.6.1.1]

cDNAv1_0.fasta.screen.Contig702

[K01881](http://www.genome.jp/dbget-bin/www_bget?ko+K01881) E6.1.1.15, proS; prolyl-tRNA synthetase [EC:6.1.1.15]

cDNAv1_0.fasta.screen.Contig243

00350 Tyrosine metabolism [PATH:ko00350]

[K00813](http://www.genome.jp/dbget-bin/www_bget?ko+K00813) E2.6.1.1B, aspC; aspartate aminotransferase [EC:2.6.1.1]

cDNAv1_0.fasta.screen.Contig702

[K00001](http://www.genome.jp/dbget-bin/www_bget?ko+K00001) E1.1.1.1, adh; alcohol dehydrogenase [EC:1.1.1.1]

cDNAv1_0.fasta.screen.Contig685

00360 Phenylalanine metabolism [PATH:ko00360]

[K00813](http://www.genome.jp/dbget-bin/www_bget?ko+K00813) E2.6.1.1B, aspC; aspartate aminotransferase [EC:2.6.1.1]

cDNAv1_0.fasta.screen.Contig702

00380 Tryptophan metabolism [PATH:ko00380]

[K00486](http://www.genome.jp/dbget-bin/www_bget?ko+K00486) E1.14.13.9; kynurenine 3-monooxygenase [EC:1.14.13.9]

cDNAv1_0.fasta.screen.Contig936

[K00164](http://www.genome.jp/dbget-bin/www_bget?ko+K00164) E1.2.4.2, sucA; 2-oxoglutarate dehydrogenase E1 component [EC:1.2.4.2]

1h12uni.t.ab1

[K03782](http://www.genome.jp/dbget-bin/www_bget?ko+K03782) KATG; catalase/peroxidase [EC:1.11.1.6]

cDNAv1_0.fasta.screen.Contig327

00400 Phenylalanine, tyrosine and tryptophan biosynthesis [PATH:ko00400]

[K01817](http://www.genome.jp/dbget-bin/www_bget?ko+K01817) E5.3.1.24, trpF; phosphoribosylanthranilate isomerase [EC:5.3.1.24]

cDNAv1_0.fasta.screen.Contig1051

[K00813](http://www.genome.jp/dbget-bin/www_bget?ko+K00813) E2.6.1.1B, aspC; aspartate aminotransferase [EC:2.6.1.1]

cDNAv1_0.fasta.screen.Contig702

**01160 Metabolism of Other Amino Acids**

00450 Selenoamino acid metabolism [PATH:ko00450]

[K01874](http://www.genome.jp/dbget-bin/www_bget?ko+K01874) E6.1.1.10, metG; methionyl-tRNA synthetase [EC:6.1.1.10]

cDNAv1_0.fasta.screen.Contig635

[K01738](http://www.genome.jp/dbget-bin/www_bget?ko+K01738) E2.5.1.47, cysK; cysteine synthase [EC:2.5.1.47]

cDNAv1_0.fasta.screen.Contig932

00480 Glutathione metabolism [PATH:ko00480]

[K01256](http://www.genome.jp/dbget-bin/www_bget?ko+K01256) E3.4.11.2, pepN; membrane alanyl aminopeptidase [EC:3.4.11.2]

cDNAv1_0.fasta.screen.Contig529

[K01920](http://www.genome.jp/dbget-bin/www_bget?ko+K01920) E6.3.2.3, gshB; glutathione synthase [EC:6.3.2.3]

cDNAv1_0.fasta.screen.Contig315

**01170 Glycan Biosynthesis and Metabolism**

00510 N-Glycan biosynthesis [PATH:ko00510]

[K01001](http://www.genome.jp/dbget-bin/www_bget?ko+K01001) E2.7.8.15, ALG7; UDP-N-acetylglucosamine--dolichyl-phosphate N-acetylglucosaminephosphstransferase [EC:2.7.8.15]

cDNAv1_0.fasta.screen.Contig465

[K03842](http://www.genome.jp/dbget-bin/www_bget?ko+K03842) ALG1; beta-1,4-mannosyltransferase [EC:2.4.1.142]

cDNAv1_0.fasta.screen.Contig802

[K00730](http://www.genome.jp/dbget-bin/www_bget?ko+K00730) E2.4.1.119; dolichyl-diphosphooligosaccharide--protein glycosyltransferase [EC:2.4.1.119]

cDNAv1_0.fasta.screen.Contig145

[K07252](http://www.genome.jp/dbget-bin/www_bget?ko+K07252) E3.6.1.43; dolichyldiphosphatase [EC:3.6.1.43]

cDNAv1_0.fasta.screen.Contig598

00531 Glycosaminoglycan degradation [PATH:ko00531]

[K01217](http://www.genome.jp/dbget-bin/www_bget?ko+K01217) E3.2.1.76, IDUA; L-iduronidase [EC:3.2.1.76]

cDNAv1_0.fasta.screen.Contig417

[K01137](http://www.genome.jp/dbget-bin/www_bget?ko+K01137) E3.1.6.14; N-acetylglucosamine-6-sulfatase [EC:3.1.6.14]

cDNAv1_0.fasta.screen.Contig285

00550 Peptidoglycan biosynthesis [PATH:ko00550]

[K01915](http://www.genome.jp/dbget-bin/www_bget?ko+K01915) E6.3.1.2, glnA; glutamine synthetase [EC:6.3.1.2]

cDNAv1_0.fasta.screen.Contig173

00600 Glycosphingolipid metabolism [PATH:ko00600]

[K00654](http://www.genome.jp/dbget-bin/www_bget?ko+K00654) E2.3.1.50; serine palmitoyltransferase [EC:2.3.1.50]

cDNAv1_0.fasta.screen.Contig757

[K04708](http://www.genome.jp/dbget-bin/www_bget?ko+K04708) E1.1.1.102; 3-dehydrosphinganine reductase [EC:1.1.1.102]

cDNAv1_0.fasta.screen.Contig363

[K00720](http://www.genome.jp/dbget-bin/www_bget?ko+K00720) E2.4.1.80, UGCG; ceramide glucosyltransferase [EC:2.4.1.80]

cDNAv1_0.fasta.screen.Contig1052

**01190 Metabolism of Cofactors and Vitamins**

00740 Riboflavin metabolism [PATH:ko00740]

[K01497](http://www.genome.jp/dbget-bin/www_bget?ko+K01497) E3.5.4.25, ribA; GTP cyclohydrolase II [EC:3.5.4.25]

cDNAv1_0.fasta.screen.Contig108

[K00953](http://www.genome.jp/dbget-bin/www_bget?ko+K00953) E2.7.7.2, ribF; FMN adenylyltransferase [EC:2.7.7.2]

11e02uni.e.ab1

00750 Vitamin B6 metabolism [PATH:ko00750]

00760 Nicotinate and nicotinamide metabolism [PATH:ko00760]

[K01950](http://www.genome.jp/dbget-bin/www_bget?ko+K01950) E6.3.5.1, nadE; NAD+ synthase (glutamine-hydrolysing) [EC:6.3.5.1]

cDNAv1_0.fasta.screen.Contig855

[K00924](http://www.genome.jp/dbget-bin/www_bget?ko+K00924) E2.7.1.-; [EC:2.7.1.-]

cDNAv1_0.fasta.screen.Contig95

2e10uni.e.ab1

[K01463](http://www.genome.jp/dbget-bin/www_bget?ko+K01463) E3.5.1.-; [EC:3.5.1.-]

cDNAv1_0.fasta.screen.Contig1045

00770 Pantothenate and CoA biosynthesis [PATH:ko00770]

[K00867](http://www.genome.jp/dbget-bin/www_bget?ko+K00867) E2.7.1.33, coaA; pantothenate kinase [EC:2.7.1.33]

cDNAv1_0.fasta.screen.Contig365

[K01463](http://www.genome.jp/dbget-bin/www_bget?ko+K01463) E3.5.1.-; [EC:3.5.1.-]

cDNAv1_0.fasta.screen.Contig1045

00780 Biotin metabolism [PATH:ko00780]

[K00652](http://www.genome.jp/dbget-bin/www_bget?ko+K00652) E2.3.1.47, bioF; 8-amino-7-oxononanoate synthase [EC:2.3.1.47]

cDNAv1_0.fasta.screen.Contig888

[K01423](http://www.genome.jp/dbget-bin/www_bget?ko+K01423) E3.4.-.-; [EC:3.4.-.-]

cDNAv1_0.fasta.screen.Contig187

00790 Folate biosynthesis [PATH:ko00790]

[K01529](http://www.genome.jp/dbget-bin/www_bget?ko+K01529) E3.6.1.-; [EC:3.6.1.-]

cDNAv1_0.fasta.screen.Contig659

cDNAv1_0.fasta.screen.Contig818

cDNAv1_0.fasta.screen.Contig879

00860 Porphyrin and chlorophyll metabolism [PATH:ko00860]

[K01885](http://www.genome.jp/dbget-bin/www_bget?ko+K01885) E6.1.1.17, gltX; glutamyl-tRNA synthetase [EC:6.1.1.17]

cDNAv1_0.fasta.screen.Contig880

[K01698](http://www.genome.jp/dbget-bin/www_bget?ko+K01698) E4.2.1.24, hemB; porphobilinogen synthase [EC:4.2.1.24]

11g04uni.f.ab1

[K01599](http://www.genome.jp/dbget-bin/www_bget?ko+K01599) E4.1.1.37, hemE; uroporphyrinogen decarboxylase [EC:4.1.1.37]

cDNAv1_0.fasta.screen.Contig74

[K00228](http://www.genome.jp/dbget-bin/www_bget?ko+K00228) E1.3.3.3, hemF; coproporphyrinogen III oxidase [EC:1.3.3.3]

cDNAv1_0.fasta.screen.Contig764

[K01764](http://www.genome.jp/dbget-bin/www_bget?ko+K01764) E4.4.1.17; cytochrome c heme-lyase [EC:4.4.1.17]

cDNAv1_0.fasta.screen.Contig856

00130 Ubiquinone biosynthesis [PATH:ko00130]

[K00334](http://www.genome.jp/dbget-bin/www_bget?ko+K00334) NUOE; NADH dehydrogenase I chain E [EC:1.6.5.3]

cDNAv1_0.fasta.screen.Contig537

[K00336](http://www.genome.jp/dbget-bin/www_bget?ko+K00336) NUOG; NADH dehydrogenase I chain G [EC:1.6.5.3]

cDNAv1_0.fasta.screen.Contig180

[K00338](http://www.genome.jp/dbget-bin/www_bget?ko+K00338) NUOI; NADH dehydrogenase I chain I [EC:1.6.5.3]

cDNAv1_0.fasta.screen.Contig268

**01195 Biosynthesis of Secondary Metabolites**

00900 Terpenoid biosynthesis [PATH:ko00900]

[K00801](http://www.genome.jp/dbget-bin/www_bget?ko+K00801) E2.5.1.21, FDFT1; farnesyl-diphosphate farnesyltransferase [EC:2.5.1.21]

cDNAv1_0.fasta.screen.Contig911

[K00511](http://www.genome.jp/dbget-bin/www_bget?ko+K00511) E1.14.99.7, SQLE; squalene monooxygenase [EC:1.14.99.7]

cDNAv1_0.fasta.screen.Contig970

00950 Alkaloid biosynthesis I [PATH:ko00950]

[K00813](http://www.genome.jp/dbget-bin/www_bget?ko+K00813) E2.6.1.1B, aspC; aspartate aminotransferase [EC:2.6.1.1]

cDNAv1_0.fasta.screen.Contig702

00521 Streptomycin biosynthesis [PATH:ko00521]

[K00844](http://www.genome.jp/dbget-bin/www_bget?ko+K00844) E2.7.1.1; hexokinase [EC:2.7.1.1]

cDNAv1_0.fasta.screen.Contig604

[K01835](http://www.genome.jp/dbget-bin/www_bget?ko+K01835) E5.4.2.2, pgm; phosphoglucomutase [EC:5.4.2.2]

cDNAv1_0.fasta.screen.Contig398

00401 Novobiocin biosynthesis [PATH:ko00401]

[K00813](http://www.genome.jp/dbget-bin/www_bget?ko+K00813) E2.6.1.1B, aspC; aspartate aminotransferase [EC:2.6.1.1]

cDNAv1_0.fasta.screen.Contig702

**01196 Xenobiotics Biodegradation and Metabolism**

00930 Caprolactam degradation [PATH:ko00930]

[K01463](http://www.genome.jp/dbget-bin/www_bget?ko+K01463) E3.5.1.-; [EC:3.5.1.-]

cDNAv1_0.fasta.screen.Contig1045

00632 Benzoate degradation via CoA ligation [PATH:ko00632]

[K00924](http://www.genome.jp/dbget-bin/www_bget?ko+K00924) E2.7.1.-; [EC:2.7.1.-]

cDNAv1_0.fasta.screen.Contig95

2e10uni.e.ab1

[K00239](http://www.genome.jp/dbget-bin/www_bget?ko+K00239) SDHA; succinate dehydrogenase flavoprotein subunit [EC:1.3.99.1]

cDNAv1_0.fasta.screen.Contig722

00624 1- and 2-Methylnaphthalene degradation [PATH:ko00624]

[K00001](http://www.genome.jp/dbget-bin/www_bget?ko+K00001) E1.1.1.1, adh; alcohol dehydrogenase [EC:1.1.1.1]

cDNAv1_0.fasta.screen.Contig685

00980 Metabolism of xenobiotics by cytochrome P450 [PATH:ko00980]

[K00001](http://www.genome.jp/dbget-bin/www_bget?ko+K00001) E1.1.1.1, adh; alcohol dehydrogenase [EC:1.1.1.1]

cDNAv1_0.fasta.screen.Contig685

**01210 Transcription**

03020 RNA polymerase [PATH:ko03020]

[K03006](http://www.genome.jp/dbget-bin/www_bget?ko+K03006) RPB1; DNA-directed RNA polymerase II subunit A [EC:2.7.7.6]

cDNAv1_0.fasta.screen.Contig167

[K03012](http://www.genome.jp/dbget-bin/www_bget?ko+K03012) RPB4; DNA-directed RNA polymerase II subunit D [EC:2.7.7.6]

cDNAv1_0.fasta.screen.Contig594

[K03013](http://www.genome.jp/dbget-bin/www_bget?ko+K03013) RPB5; DNA-directed RNA polymerase II subunit E [EC:2.7.7.6]

cDNAv1_0.fasta.screen.Contig364

[K03025](http://www.genome.jp/dbget-bin/www_bget?ko+K03025) RPC34; DNA-directed RNA polymerase III subunit C34 [EC:2.7.7.6]

cDNAv1_0.fasta.screen.Contig603

[K02999](http://www.genome.jp/dbget-bin/www_bget?ko+K02999) RPA1; DNA-directed RNA polymerase I subunit A1 [EC:2.7.7.6]

cDNAv1_0.fasta.screen.Contig532

[K03000](http://www.genome.jp/dbget-bin/www_bget?ko+K03000) RPA12; DNA-directed RNA polymerase I subunit A12 [EC:2.7.7.6]

cDNAv1_0.fasta.screen.Contig559

03022 Basal transcription factors [PATH:ko03022]

[K03120](http://www.genome.jp/dbget-bin/www_bget?ko+K03120) TBP; transcription initiation factor TFIID TATA binding protein

cDNAv1_0.fasta.screen.Contig947

[K03131](http://www.genome.jp/dbget-bin/www_bget?ko+K03131) TFIID5; transcription initiation factor TFIID subunit D5

17c08uni.e.ab1

[K03126](http://www.genome.jp/dbget-bin/www_bget?ko+K03126) TFIID10; transcription initiation factor TFIID subunit D10

cDNAv1_0.fasta.screen.Contig796

[K03124](http://www.genome.jp/dbget-bin/www_bget?ko+K03124) TFIIB; transcription initiation factor TFIIB

cDNAv1_0.fasta.screen.Contig637

[K03122](http://www.genome.jp/dbget-bin/www_bget?ko+K03122) TFIIA1; transcription initiation factor TFIIA large subunit

cDNAv1_0.fasta.screen.Contig561

[K03138](http://www.genome.jp/dbget-bin/www_bget?ko+K03138) TFIIF1; transcription initiation factor TFIIF alpha subunit

cDNAv1_0.fasta.screen.Contig655

[K03136](http://www.genome.jp/dbget-bin/www_bget?ko+K03136) TFIIE1; transcription initiation factor TFIIE alpha subunit

cDNAv1_0.fasta.screen.Contig742

[K03145](http://www.genome.jp/dbget-bin/www_bget?ko+K03145) TFIIS; transcription elongation factor S-II

cDNAv1_0.fasta.screen.Contig549

[K01974](http://www.genome.jp/dbget-bin/www_bget?ko+K01974) E6.5.1.4; RNA-3'-phosphate cyclase [EC:6.5.1.4]

cDNAv1_0.fasta.screen.Contig94

**01220 Translation**

03010 Ribosome [PATH:ko03010] [BR:ko03010]

[K02967](http://www.genome.jp/dbget-bin/www_bget?ko+K02967) RP-S2, rpsB; small subunit ribosomal protein S2

cDNAv1_0.fasta.screen.Contig558

[K02988](http://www.genome.jp/dbget-bin/www_bget?ko+K02988) RP-S5, rpsE; small subunit ribosomal protein S5

cDNAv1_0.fasta.screen.Contig478

[K02985](http://www.genome.jp/dbget-bin/www_bget?ko+K02985) RP-S3e, RPS3; small subunit ribosomal protein S3e

cDNAv1_0.fasta.screen.Contig627

[K02987](http://www.genome.jp/dbget-bin/www_bget?ko+K02987) RP-S4e, RPS4; small subunit ribosomal protein S4e

cDNAv1_0.fasta.screen.Contig900

[K02989](http://www.genome.jp/dbget-bin/www_bget?ko+K02989) RP-S5e, RPS5; small subunit ribosomal protein S5e

cDNAv1_0.fasta.screen.Contig290

[K02906](http://www.genome.jp/dbget-bin/www_bget?ko+K02906) RP-L3, rplC; large subunit ribosomal protein L3

cDNAv1_0.fasta.screen.Contig455

[K02925](http://www.genome.jp/dbget-bin/www_bget?ko+K02925) RP-L3e, RPL3; large subunit ribosomal protein L3e

cDNAv1_0.fasta.screen.Contig164

[K02930](http://www.genome.jp/dbget-bin/www_bget?ko+K02930) RP-L4e, RPL4; large subunit ribosomal protein L4e

cDNAv1_0.fasta.screen.Contig36

cDNAv1_0.fasta.screen.Contig908

[K02936](http://www.genome.jp/dbget-bin/www_bget?ko+K02936) RP-L7Ae, RPL7A; large subunit ribosomal protein L7Ae

cDNAv1_0.fasta.screen.Contig299

[K02908](http://www.genome.jp/dbget-bin/www_bget?ko+K02908) RP-L30e, RPL30; large subunit ribosomal protein L30e

cDNAv1_0.fasta.screen.Contig311

[K02912](http://www.genome.jp/dbget-bin/www_bget?ko+K02912) RP-L32e, RPL32; large subunit ribosomal protein L32e

cDNAv1_0.fasta.screen.Contig971

[K02920](http://www.genome.jp/dbget-bin/www_bget?ko+K02920) RP-L36e, RPL36; large subunit ribosomal protein L36e

cDNAv1_0.fasta.screen.Contig277

[K02941](http://www.genome.jp/dbget-bin/www_bget?ko+K02941) RP-LP0, RPLP0; large subunit ribosomal protein LP0

cDNAv1_0.fasta.screen.Contig227

[K03236](http://www.genome.jp/dbget-bin/www_bget?ko+K03236) eIF-1A, EIF1A; translation initiation factor eIF-1A

cDNAv1_0.fasta.screen.Contig228

[K03237](http://www.genome.jp/dbget-bin/www_bget?ko+K03237) eIF-2A, EIF2S1; translation initiation factor eIF-2 alpha subunit

cDNAv1_0.fasta.screen.Contig481

[K03238](http://www.genome.jp/dbget-bin/www_bget?ko+K03238) eIF-2B, EIF2S2; translation initiation factor eIF-2 beta subunit

cDNAv1_0.fasta.screen.Contig650

[K03680](http://www.genome.jp/dbget-bin/www_bget?ko+K03680) eIF-2BD, EIF2B4; translation initiation factor eIF-2B delta subunit

cDNAv1_0.fasta.screen.Contig907

[K03240](http://www.genome.jp/dbget-bin/www_bget?ko+K03240) eIF-2BE; translation initiation factor eIF-2B epsilon subunit

cDNAv1_0.fasta.screen.Contig850

[K03262](http://www.genome.jp/dbget-bin/www_bget?ko+K03262) eIF-5, EIF5; translation initiation factor eIF-5

cDNAv1_0.fasta.screen.Contig917

[K03246](http://www.genome.jp/dbget-bin/www_bget?ko+K03246) eIF-32, EIF3S2; translation initiation factor eIF-3 subunit 2

cDNAv1_0.fasta.screen.Contig421

[K03247](http://www.genome.jp/dbget-bin/www_bget?ko+K03247) eIF-33, EIF3S3; translation initiation factor eIF-3 subunit 3

cDNAv1_0.fasta.screen.Contig505

[K03248](http://www.genome.jp/dbget-bin/www_bget?ko+K03248) eIF-34, EIF3S4; translation initiation factor eIF-3 subunit 4

cDNAv1_0.fasta.screen.Contig247

[K03251](http://www.genome.jp/dbget-bin/www_bget?ko+K03251) eIF-37, EIF3S7; translation initiation factor eIF-3 subunit 7

14g11uni.f.ab1

[K03253](http://www.genome.jp/dbget-bin/www_bget?ko+K03253) eIF-39, EIF3S9; translation initiation factor eIF-3 subunit 9

cDNAv1_0.fasta.screen.Contig396

[K03256](http://www.genome.jp/dbget-bin/www_bget?ko+K03256) eIF-3Y; translation initiation factor eIF-3 subunit P62

cDNAv1_0.fasta.screen.Contig129

[K03257](http://www.genome.jp/dbget-bin/www_bget?ko+K03257) eIF-4A, EIF4A; translation initiation factor eIF-4A

cDNAv1_0.fasta.screen.Contig103

[K03258](http://www.genome.jp/dbget-bin/www_bget?ko+K03258) eIF-4B, EIF4B; translation initiation factor eIF-4B

cDNAv1_0.fasta.screen.Contig697

[K02355](http://www.genome.jp/dbget-bin/www_bget?ko+K02355) EF-G, fusA; elongation factor EF-G [EC:3.6.5.3]

cDNAv1_0.fasta.screen.Contig565

[K03231](http://www.genome.jp/dbget-bin/www_bget?ko+K03231) eEF-1A, ef1A; elongation factor EF-1 alpha subunit [EC:3.6.5.3]

cDNAv1_0.fasta.screen.Contig123

cDNAv1_0.fasta.screen.Contig298

[K03233](http://www.genome.jp/dbget-bin/www_bget?ko+K03233) eEF-1G, ef1G; elongation factor EF-1 gamma subunit

cDNAv1_0.fasta.screen.Contig351

[K03235](http://www.genome.jp/dbget-bin/www_bget?ko+K03235) eEF-3; elongation factor EF-3

cDNAv1_0.fasta.screen.Contig314

[K03266](http://www.genome.jp/dbget-bin/www_bget?ko+K03266) eRF-2; peptide chain release factor eRF subunit 2

cDNAv1_0.fasta.screen.Contig566

00970 Aminoacyl-tRNA biosynthesis [PATH:ko00970]

[K01874](http://www.genome.jp/dbget-bin/www_bget?ko+K01874) E6.1.1.10, metG; methionyl-tRNA synthetase [EC:6.1.1.10]

cDNAv1_0.fasta.screen.Contig635

[K01883](http://www.genome.jp/dbget-bin/www_bget?ko+K01883) E6.1.1.16A, cysS; cysteinyl-tRNA synthetase [EC:6.1.1.16]

cDNAv1_0.fasta.screen.Contig203

cDNAv1_0.fasta.screen.Contig509

[K01885](http://www.genome.jp/dbget-bin/www_bget?ko+K01885) E6.1.1.17, gltX; glutamyl-tRNA synthetase [EC:6.1.1.17]

cDNAv1_0.fasta.screen.Contig880

[K01875](http://www.genome.jp/dbget-bin/www_bget?ko+K01875) E6.1.1.11, serS; seryl-tRNA synthetase [EC:6.1.1.11]

cDNAv1_0.fasta.screen.Contig989

[K01881](http://www.genome.jp/dbget-bin/www_bget?ko+K01881) E6.1.1.15, proS; prolyl-tRNA synthetase [EC:6.1.1.15]

cDNAv1_0.fasta.screen.Contig243

[K04567](http://www.genome.jp/dbget-bin/www_bget?ko+K04567) LYSU, KARS; lysyl-tRNA synthetase, class II [EC:6.1.1.6]

cDNAv1_0.fasta.screen.Contig790

cDNAv1_0.fasta.screen.Contig1047

[K01880](http://www.genome.jp/dbget-bin/www_bget?ko+K01880) E6.1.1.14C, GRS1; glycyl-tRNA synthetase, class II [EC:6.1.1.14]

12f12uni.e.ab1

[K01147](http://www.genome.jp/dbget-bin/www_bget?ko+K01147) E3.1.13.1, rnb; exoribonuclease II [EC:3.1.13.1]

cDNAv1_0.fasta.screen.Contig930

[K03685](http://www.genome.jp/dbget-bin/www_bget?ko+K03685) RNC; ribonuclease III [EC:3.1.26.3]

cDNAv1_0.fasta.screen.Contig56

[K03684](http://www.genome.jp/dbget-bin/www_bget?ko+K03684) RND; ribonuclease D [EC:3.1.26.3]

cDNAv1_0.fasta.screen.Contig411

[K03500](http://www.genome.jp/dbget-bin/www_bget?ko+K03500) SUN; Sun protein

cDNAv1_0.fasta.screen.Contig284

[K02434](http://www.genome.jp/dbget-bin/www_bget?ko+K02434) GATB; glutamyl-tRNA (Gln) amidotransferase subunit B [EC:6.3.5.-]

cDNAv1_0.fasta.screen.Contig919

[K04795](http://www.genome.jp/dbget-bin/www_bget?ko+K04795) FLPA; fibrillarin-like pre-rRNA processing protein

cDNAv1_0.fasta.screen.Contig998

[K00791](http://www.genome.jp/dbget-bin/www_bget?ko+K00791) E2.5.1.8, miaA; tRNA delta(2)-isopentenylpyrophosphate transferase [EC:2.5.1.8]

cDNAv1_0.fasta.screen.Contig23

cDNAv1_0.fasta.screen.Contig905

[K00586](http://www.genome.jp/dbget-bin/www_bget?ko+K00586) E2.1.1.98, dph5; diphthine synthase [EC:2.1.1.98]

cDNAv1_0.fasta.screen.Contig869

[K00773](http://www.genome.jp/dbget-bin/www_bget?ko+K00773) E2.4.2.29, tgt; queuine tRNA-ribosyltransferase [EC:2.4.2.29]

cDNAv1_0.fasta.screen.Contig265

[K00809](http://www.genome.jp/dbget-bin/www_bget?ko+K00809) E2.5.1.46, dys1; deoxyhypusine synthase [EC:2.5.1.46]

cDNAv1_0.fasta.screen.Contig508

[K00970](http://www.genome.jp/dbget-bin/www_bget?ko+K00970) E2.7.7.19, pcnB; poly(A) polymerase [EC:2.7.7.19]

cDNAv1_0.fasta.screen.Contig1000

[K04077](http://www.genome.jp/dbget-bin/www_bget?ko+K04077) GROEL; chaperonin GroEL (Hsp60)

cDNAv1_0.fasta.screen.Contig1006

[K03283](http://www.genome.jp/dbget-bin/www_bget?ko+K03283) TC.HSP70; heat shock protein 70, Hsp70 family [TC:1.A.33]

cDNAv1_0.fasta.screen.Contig1035

[K04043](http://www.genome.jp/dbget-bin/www_bget?ko+K04043) DNAK; molecular chaperone DnaK (Hsp70) [TC:1.A.33]

cDNAv1_0.fasta.screen.Contig406

[K03686](http://www.genome.jp/dbget-bin/www_bget?ko+K03686) DNAJ; molecular chaperone DnaJ

cDNAv1_0.fasta.screen.Contig599

[K04079](http://www.genome.jp/dbget-bin/www_bget?ko+K04079) HTPG, HSP90; molecular chaperone HtpG (Hsp90)

cDNAv1_0.fasta.screen.Contig1039

[K03798](http://www.genome.jp/dbget-bin/www_bget?ko+K03798) FTSH, hflB; ATP-dependent Zn protease, cell division protein [EC:3.4.24.-]

cDNAv1_0.fasta.screen.Contig943

[K01829](http://www.genome.jp/dbget-bin/www_bget?ko+K01829) E5.3.4.1; protein disulfide-isomerase [EC:5.3.4.1]

cDNAv1_0.fasta.screen.Contig723

[K08056](http://www.genome.jp/dbget-bin/www_bget?ko+K08056) PDIA3, GRP58; protein disulfide isomerase family A, member 3 [EC:5.3.4.1]

cDNAv1_0.fasta.screen.Contig724

[K00685](http://www.genome.jp/dbget-bin/www_bget?ko+K00685) E2.3.2.8, ate1; arginine-tRNA-protein transferase [EC:2.3.2.8]

cDNAv1_0.fasta.screen.Contig584

[K01409](http://www.genome.jp/dbget-bin/www_bget?ko+K01409) E3.4.24.57, gcp; O-sialoglycoprotein endopeptidase [EC:3.4.24.57]

cDNAv1_0.fasta.screen.Contig852

[K01802](http://www.genome.jp/dbget-bin/www_bget?ko+K01802) E5.2.1.8; peptidylprolyl isomerase [EC:5.2.1.8]

cDNAv1_0.fasta.screen.Contig301

cDNAv1_0.fasta.screen.Contig332

cDNAv1_0.fasta.screen.Contig401

cDNAv1_0.fasta.screen.Contig521

5b02uni.t.ab1

[K03671](http://www.genome.jp/dbget-bin/www_bget?ko+K03671) TRXA; thioredoxin 1

cDNAv1_0.fasta.screen.Contig319

[K07390](http://www.genome.jp/dbget-bin/www_bget?ko+K07390) K07390; monothiol glutaredoxin

cDNAv1_0.fasta.screen.Contig122

**01230 Folding, Sorting and Degradation**

03060 Protein export [PATH:ko03060]

[K03076](http://www.genome.jp/dbget-bin/www_bget?ko+K03076) SECY; preprotein translocase SecY subunit [TC:3.A.5]

cDNAv1_0.fasta.screen.Contig767

[K03106](http://www.genome.jp/dbget-bin/www_bget?ko+K03106) SRP54, ffh; signal recognition particle, subunit SRP54

cDNAv1_0.fasta.screen.Contig812

[K03110](http://www.genome.jp/dbget-bin/www_bget?ko+K03110) SRPR, ftsY; signal recognition particle receptor

cDNAv1_0.fasta.screen.Contig735

03090 Type II secretion system [PATH:ko03090]

[K01493](http://www.genome.jp/dbget-bin/www_bget?ko+K01493) E3.5.4.12, comEB; dCMP deaminase [EC:3.5.4.12]

cDNAv1_0.fasta.screen.Contig120

04120 Ubiquitin mediated proteolysis [PATH:ko04120]

[K03355](http://www.genome.jp/dbget-bin/www_bget?ko+K03355) APC8, CDC23; anaphase-promoting complex component APC8

cDNAv1_0.fasta.screen.Contig746

[K03874](http://www.genome.jp/dbget-bin/www_bget?ko+K03874) HECT; hect domain ubiquitin protein ligase E3 component [EC:6.3.2.-]

cDNAv1_0.fasta.screen.Contig198

2a05uni.e.ab1

[K03364](http://www.genome.jp/dbget-bin/www_bget?ko+K03364) CDH1; cell division cycle 20 homolog 1, cofactor of APC complex

cDNAv1_0.fasta.screen.Contig414

03050 Proteasome [PATH:ko03050]

[K02730](http://www.genome.jp/dbget-bin/www_bget?ko+K02730) PSMA6; 20S proteasome subunit alpha 1 [EC:3.4.25.1]

cDNAv1_0.fasta.screen.Contig613

[K02726](http://www.genome.jp/dbget-bin/www_bget?ko+K02726) PSMA2; 20S proteasome subunit alpha 2 [EC:3.4.25.1]

cDNAv1_0.fasta.screen.Contig321

[K02728](http://www.genome.jp/dbget-bin/www_bget?ko+K02728) PSMA4; 20S proteasome subunit alpha 3 [EC:3.4.25.1]

cDNAv1_0.fasta.screen.Contig653

[K02729](http://www.genome.jp/dbget-bin/www_bget?ko+K02729) PSMA5; 20S proteasome subunit alpha 5 [EC:3.4.25.1]

cDNAv1_0.fasta.screen.Contig542

[K03032](http://www.genome.jp/dbget-bin/www_bget?ko+K03032) RPN2, PSMD1; 26S proteasome regulatory subunit N2

cDNAv1_0.fasta.screen.Contig609

[K03033](http://www.genome.jp/dbget-bin/www_bget?ko+K03033) RPN3, PSMD3; 26S proteasome regulatory subunit N3

cDNAv1_0.fasta.screen.Contig668

[K03039](http://www.genome.jp/dbget-bin/www_bget?ko+K03039) RPN9, PSMD13; 26S proteasome regulatory subunit N9

cDNAv1_0.fasta.screen.Contig740

[K03029](http://www.genome.jp/dbget-bin/www_bget?ko+K03029) RPN10, PSMD4; 26S proteasome regulatory subunit N10

cDNAv1_0.fasta.screen.Contig415

[K03065](http://www.genome.jp/dbget-bin/www_bget?ko+K03065) RPT5, PSMC3; 26S proteasome regulatory subunit T5

cDNAv1_0.fasta.screen.Contig416

[K01418](http://www.genome.jp/dbget-bin/www_bget?ko+K01418) E3.4.25.1; proteasome [EC:3.4.25.1]

17b03uni.e.ab1

**01240 Replication and Repair**

03030 DNA polymerase [PATH:ko03030]

[K02325](http://www.genome.jp/dbget-bin/www_bget?ko+K02325) POLE2; DNA Polymerase epsilon, subunit B [EC:2.7.7.7]

cDNAv1_0.fasta.screen.Contig921

[K02470](http://www.genome.jp/dbget-bin/www_bget?ko+K02470) GYRB; DNA gyrase subunit B [EC:5.99.1.3]

cDNAv1_0.fasta.screen.Contig744

[K03165](http://www.genome.jp/dbget-bin/www_bget?ko+K03165) TOP3; DNA topoisomerase III [EC:5.99.1.2]

cDNAv1_0.fasta.screen.Contig546

[K03469](http://www.genome.jp/dbget-bin/www_bget?ko+K03469) E3.1.26.4A, rnhA; ribonuclease HI [EC:3.1.26.4]

cDNAv1_0.fasta.screen.Contig484

[K04483](http://www.genome.jp/dbget-bin/www_bget?ko+K04483) RADA; DNA repair protein RadA

cDNAv1_0.fasta.screen.Contig144

[K03648](http://www.genome.jp/dbget-bin/www_bget?ko+K03648) UNG; uracil-DNA glycosylase [EC:3.2.2.-]

cDNAv1_0.fasta.screen.Contig255

[K04799](http://www.genome.jp/dbget-bin/www_bget?ko+K04799) FEN1, RAD2; flap endonuclease-1 [EC:3.-.-.-]

cDNAv1_0.fasta.screen.Contig593

[K03578](http://www.genome.jp/dbget-bin/www_bget?ko+K03578) HRPA; ATP-dependent helicase HrpA [EC:3.6.1.-]

cDNAv1_0.fasta.screen.Contig845

[K03654](http://www.genome.jp/dbget-bin/www_bget?ko+K03654) RECQ; ATP-dependent DNA helicase RecQ [EC:3.6.1.-]

cDNAv1_0.fasta.screen.Contig280

[K05592](http://www.genome.jp/dbget-bin/www_bget?ko+K05592) DEAD; ATP-dependent RNA helicase DeaD

cDNAv1_0.fasta.screen.Contig1025

[K04801](http://www.genome.jp/dbget-bin/www_bget?ko+K04801) RFCS; replication factor C, small subunit

cDNAv1_0.fasta.screen.Contig860

**01310 Membrane Transport**

02010 ABC transporters [PATH:ko02010] [BR:ko02000] [TC:3.A.1]

[K03295](http://www.genome.jp/dbget-bin/www_bget?ko+K03295) TC.CDF; cation efflux system protein, CDF family [TC:2.A.4]

cDNAv1_0.fasta.screen.Contig765

[K03305](http://www.genome.jp/dbget-bin/www_bget?ko+K03305) TC.POT; proton-dependent oligopeptide transporter, POT family [TC:2.A.17]

cDNAv1_0.fasta.screen.Contig968

[K03454](http://www.genome.jp/dbget-bin/www_bget?ko+K03454) TC.MC; mitochondrial carrier protein, MC family [TC:2.A.29]

cDNAv1_0.fasta.screen.Contig639

[K05863](http://www.genome.jp/dbget-bin/www_bget?ko+K05863) ANT; solute carrier family 25 (mitochondrial carrier; adenine nucleotide translocator) [TC:2.A.29]

cDNAv1_0.fasta.screen.Contig974

[K03283](http://www.genome.jp/dbget-bin/www_bget?ko+K03283) TC.HSP70; heat shock protein 70, Hsp70 family [TC:1.A.33]

cDNAv1_0.fasta.screen.Contig1035

[K04043](http://www.genome.jp/dbget-bin/www_bget?ko+K04043) DNAK; molecular chaperone DnaK (Hsp70) [TC:1.A.33]

cDNAv1_0.fasta.screen.Contig406

**01320 Signal Transduction**

04010 MAPK signaling pathway [PATH:ko04010hsa]

[K04371](http://www.genome.jp/dbget-bin/www_bget?ko+K04371) ERK1_2; extracellular signal-regulated kinase 1/2 [EC:2.7.11.24]

cDNAv1_0.fasta.screen.Contig541

[K04392](http://www.genome.jp/dbget-bin/www_bget?ko+K04392) RAC1; Ras-related C3 botulinum toxin substrate 1

cDNAv1_0.fasta.screen.Contig309

[K03283](http://www.genome.jp/dbget-bin/www_bget?ko+K03283) TC.HSP70; heat shock protein 70, Hsp70 family [TC:1.A.33]

cDNAv1_0.fasta.screen.Contig1035

04310 Wnt signaling pathway [PATH:ko04310]

[K03097](http://www.genome.jp/dbget-bin/www_bget?ko+K03097) CSNK2A; casein kinase 2, alpha polypeptide [EC:2.7.11.1]

cDNAv1_0.fasta.screen.Contig969

[K03083](http://www.genome.jp/dbget-bin/www_bget?ko+K03083) GSK3B; glycogen synthase kinase 3 beta [EC:2.7.11.1]

cDNAv1_0.fasta.screen.Contig822

[K03456](http://www.genome.jp/dbget-bin/www_bget?ko+K03456) PPP2R1; protein phosphatase 2 (formerly 2A), regulatory subunit A

cDNAv1_0.fasta.screen.Contig738

[K04354](http://www.genome.jp/dbget-bin/www_bget?ko+K04354) PPP2R2; protein phosphatase 2 (formerly 2A), regulatory subunit B

cDNAv1_0.fasta.screen.Contig205

[K04392](http://www.genome.jp/dbget-bin/www_bget?ko+K04392) RAC1; Ras-related C3 botulinum toxin substrate 1

cDNAv1_0.fasta.screen.Contig309

04330 Notch signaling pathway [PATH:ko04330]

[K06063](http://www.genome.jp/dbget-bin/www_bget?ko+K06063) SKIIP; SKI interacting protein

cDNAv1_0.fasta.screen.Contig939

[K06067](http://www.genome.jp/dbget-bin/www_bget?ko+K06067) HDAC1_2; histone deacetylase 1/2

cDNAv1_0.fasta.screen.Contig982

04340 Hedgehog signaling pathway [PATH:ko04340]

[K03083](http://www.genome.jp/dbget-bin/www_bget?ko+K03083) GSK3B; glycogen synthase kinase 3 beta [EC:2.7.11.1]

cDNAv1_0.fasta.screen.Contig822

[K02218](http://www.genome.jp/dbget-bin/www_bget?ko+K02218) CSNK1, CK1; casein kinase 1 [EC:2.7.11.1]

12e09uni.t.ab1

17a05uni.t.ab1

04350 TGF-beta signaling pathway [PATH:ko04350]

[K04371](http://www.genome.jp/dbget-bin/www_bget?ko+K04371) ERK1_2; extracellular signal-regulated kinase 1/2 [EC:2.7.11.24]

cDNAv1_0.fasta.screen.Contig541

[K03456](http://www.genome.jp/dbget-bin/www_bget?ko+K03456) PPP2R1; protein phosphatase 2 (formerly 2A), regulatory subunit A

cDNAv1_0.fasta.screen.Contig738

[K04354](http://www.genome.jp/dbget-bin/www_bget?ko+K04354) PPP2R2; protein phosphatase 2 (formerly 2A), regulatory subunit B

cDNAv1_0.fasta.screen.Contig205

04370 VEGF signaling pathway [PATH:ko04370]

[K04371](http://www.genome.jp/dbget-bin/www_bget?ko+K04371) ERK1_2; extracellular signal-regulated kinase 1/2 [EC:2.7.11.24]

cDNAv1_0.fasta.screen.Contig541

[K04392](http://www.genome.jp/dbget-bin/www_bget?ko+K04392) RAC1; Ras-related C3 botulinum toxin substrate 1

cDNAv1_0.fasta.screen.Contig309

04630 Jak-STAT signaling pathway [PATH:ko04630]

[K04705](http://www.genome.jp/dbget-bin/www_bget?ko+K04705) STAM; signal transducing adaptor molecule

cDNAv1_0.fasta.screen.Contig680

[K04707](http://www.genome.jp/dbget-bin/www_bget?ko+K04707) CBL; Cas-Br-M (murine) ecotropic retroviral transforming sequence [EC:6.3.2.-]

cDNAv1_0.fasta.screen.Contig686

04020 Calcium signaling pathway [PATH:ko04020]

[K05853](http://www.genome.jp/dbget-bin/www_bget?ko+K05853) ATP2A; Ca2+ transporting ATPase, sarcoplasmic/endoplasmic reticulum [EC:3.6.3.8] [TC:3.A.3.2]

cDNAv1_0.fasta.screen.Contig217

5b07uni.e.ab1

[K05863](http://www.genome.jp/dbget-bin/www_bget?ko+K05863) ANT; solute carrier family 25 (mitochondrial carrier; adenine nucleotide translocator) [TC:2.A.29]

cDNAv1_0.fasta.screen.Contig974

04070 Phosphatidylinositol signaling system [PATH:ko04070]

[K00888](http://www.genome.jp/dbget-bin/www_bget?ko+K00888) E2.7.1.67; phosphatidylinositol 4-kinase [EC:2.7.1.67]

cDNAv1_0.fasta.screen.Contig791

04910 Insulin signaling pathway [PATH:ko04910]

[K03083](http://www.genome.jp/dbget-bin/www_bget?ko+K03083) GSK3B; glycogen synthase kinase 3 beta [EC:2.7.11.1]

cDNAv1_0.fasta.screen.Contig822

[K00688](http://www.genome.jp/dbget-bin/www_bget?ko+K00688) E2.4.1.1, glgP, PYG; starch phosphorylase [EC:2.4.1.1]

cDNAv1_0.fasta.screen.Contig133

[K06269](http://www.genome.jp/dbget-bin/www_bget?ko+K06269) PPP1C; protein phosphatase 1, catalytic subunit [EC:3.1.3.16]

cDNAv1_0.fasta.screen.Contig138

[K04707](http://www.genome.jp/dbget-bin/www_bget?ko+K04707) CBL; Cas-Br-M (murine) ecotropic retroviral transforming sequence [EC:6.3.2.-]

cDNAv1_0.fasta.screen.Contig686

[K00850](http://www.genome.jp/dbget-bin/www_bget?ko+K00850) E2.7.1.11, pfk; 6-phosphofructokinase [EC:2.7.1.11]

14e02uni.f.ab1

14e02uni.t.ab1

[K07199](http://www.genome.jp/dbget-bin/www_bget?ko+K07199) PRKAB; 5'-AMP-activated protein kinase, regulatory beta subunit

cDNAv1_0.fasta.screen.Contig96

[K07204](http://www.genome.jp/dbget-bin/www_bget?ko+K07204) RAPTOR; regulatory associated protein of mTOR

cDNAv1_0.fasta.screen.Contig452

[K07207](http://www.genome.jp/dbget-bin/www_bget?ko+K07207) TSC2; tuberous sclerosis 2

cDNAv1_0.fasta.screen.Contig991

[K04371](http://www.genome.jp/dbget-bin/www_bget?ko+K04371) ERK1_2; extracellular signal-regulated kinase 1/2 [EC:2.7.11.24]

cDNAv1_0.fasta.screen.Contig541

04920 Adipocytokine signaling pathway [PATH:ko04920]

[K07199](http://www.genome.jp/dbget-bin/www_bget?ko+K07199) PRKAB; 5'-AMP-activated protein kinase, regulatory beta subunit

cDNAv1_0.fasta.screen.Contig96

[K07359](http://www.genome.jp/dbget-bin/www_bget?ko+K07359) CAMKK; calcium/calmodulin-dependent protein kinase kinase [EC:2.7.11.17]

cDNAv1_0.fasta.screen.Contig370

04150 mTOR signaling pathway [PATH:ko04150]

[K07207](http://www.genome.jp/dbget-bin/www_bget?ko+K07207) TSC2; tuberous sclerosis 2

cDNAv1_0.fasta.screen.Contig991

[K08266](http://www.genome.jp/dbget-bin/www_bget?ko+K08266) GBL; G protein beta subunit-like

cDNAv1_0.fasta.screen.Contig535

[K07204](http://www.genome.jp/dbget-bin/www_bget?ko+K07204) RAPTOR; regulatory associated protein of mTOR

cDNAv1_0.fasta.screen.Contig452

[K03258](http://www.genome.jp/dbget-bin/www_bget?ko+K03258) eIF-4B, EIF4B; translation initiation factor eIF-4B

cDNAv1_0.fasta.screen.Contig697

[K04371](http://www.genome.jp/dbget-bin/www_bget?ko+K04371) ERK1_2; extracellular signal-regulated kinase 1/2 [EC:2.7.11.24]

cDNAv1_0.fasta.screen.Contig541

[K04627](http://www.genome.jp/dbget-bin/www_bget?ko+K04627) K04627; fungal pheromone

cDNAv1_0.fasta.screen.Contig632

cDNAv1_0.fasta.screen.Contig638

[K07972](http://www.genome.jp/dbget-bin/www_bget?ko+K07972) GNB; guanine nucleotide binding protein (G protein), beta, other

cDNAv1_0.fasta.screen.Contig267

[K04392](http://www.genome.jp/dbget-bin/www_bget?ko+K04392) RAC1; Ras-related C3 botulinum toxin substrate 1

cDNAv1_0.fasta.screen.Contig309

[K07897](http://www.genome.jp/dbget-bin/www_bget?ko+K07897) RAB7; RAB7, member Ras oncogene family

cDNAv1_0.fasta.screen.Contig750

[K07976](http://www.genome.jp/dbget-bin/www_bget?ko+K07976) RAB; Rab family, other

cDNAv1_0.fasta.screen.Contig104

cDNAv1_0.fasta.screen.Contig177

cDNAv1_0.fasta.screen.Contig1019

[K07942](http://www.genome.jp/dbget-bin/www_bget?ko+K07942) ARL1; ADP-ribosylation factor-like 1

cDNAv1_0.fasta.screen.Contig114

[K07977](http://www.genome.jp/dbget-bin/www_bget?ko+K07977) ARF; Arf/Sar family, other

cDNAv1_0.fasta.screen.Contig756

[K05393](http://www.genome.jp/dbget-bin/www_bget?ko+K05393) CLCNF; chloride channel, other eukaryote [TC:1.A.11]

cDNAv1_0.fasta.screen.Contig962

**01330 Signaling Molecules and Interaction**

04514 Cell adhesion molecules (CAMs) [PATH:ko04514] [BR:ko04514]

[K06547](http://www.genome.jp/dbget-bin/www_bget?ko+K06547) ALCAM; activated leukocyte cell adhesion molecule

cDNAv1_0.fasta.screen.Contig253

[K01256](http://www.genome.jp/dbget-bin/www_bget?ko+K01256) E3.4.11.2, pepN; membrane alanyl aminopeptidase [EC:3.4.11.2]

cDNAv1_0.fasta.screen.Contig529

[K06547](http://www.genome.jp/dbget-bin/www_bget?ko+K06547) ALCAM; activated leukocyte cell adhesion molecule

cDNAv1_0.fasta.screen.Contig253

**01410 Cell Motility**

04810 Regulation of actin cytoskeleton [PATH:ko04810]

[K04371](http://www.genome.jp/dbget-bin/www_bget?ko+K04371) ERK1_2; extracellular signal-regulated kinase 1/2 [EC:2.7.11.24]

cDNAv1_0.fasta.screen.Contig541

[K04392](http://www.genome.jp/dbget-bin/www_bget?ko+K04392) RAC1; Ras-related C3 botulinum toxin substrate 1

cDNAv1_0.fasta.screen.Contig309

[K06269](http://www.genome.jp/dbget-bin/www_bget?ko+K06269) PPP1C; protein phosphatase 1, catalytic subunit [EC:3.1.3.16]

cDNAv1_0.fasta.screen.Contig138

[K05757](http://www.genome.jp/dbget-bin/www_bget?ko+K05757) ARPC1A_B; actin related protein 2/3 complex, subunit 1A/1B, 41kDa

cDNAv1_0.fasta.screen.Contig953

[K05758](http://www.genome.jp/dbget-bin/www_bget?ko+K05758) ARPC2; actin related protein 2/3 complex, subunit 2, 34kDa

cDNAv1_0.fasta.screen.Contig625

[K05692](http://www.genome.jp/dbget-bin/www_bget?ko+K05692) ACTB_G; actin, beta/gamma, cytoplasmic

cDNAv1_0.fasta.screen.Contig381

[K05767](http://www.genome.jp/dbget-bin/www_bget?ko+K05767) IQGAP; IQ motif containing GTPase activating protein

cDNAv1_0.fasta.screen.Contig124

[K05699](http://www.genome.jp/dbget-bin/www_bget?ko+K05699) ACTN; actinin, alpha

cDNAv1_0.fasta.screen.Contig105

[K03798](http://www.genome.jp/dbget-bin/www_bget?ko+K03798) FTSH, hflB; ATP-dependent Zn protease, cell division protein [EC:3.4.24.-]

cDNAv1_0.fasta.screen.Contig943

[K02427](http://www.genome.jp/dbget-bin/www_bget?ko+K02427) FTSJ; cell division protein methyltransferase FtsJ [EC:2.1.1.-]

cDNAv1_0.fasta.screen.Contig400

cDNAv1_0.fasta.screen.Contig1053

[K03529](http://www.genome.jp/dbget-bin/www_bget?ko+K03529) SMC; chromosome segregation protein

cDNAv1_0.fasta.screen.Contig958

cDNAv1_0.fasta.screen.Contig1028

[K03593](http://www.genome.jp/dbget-bin/www_bget?ko+K03593) MRP; ATP-binding protein involved in chromosome partitioning

cDNAv1_0.fasta.screen.Contig109

**01420 Cell Growth and Death**

04110 Cell cycle [PATH:ko04110hsa]

[K06067](http://www.genome.jp/dbget-bin/www_bget?ko+K06067) HDAC1_2; histone deacetylase 1/2

cDNAv1_0.fasta.screen.Contig982

[K03083](http://www.genome.jp/dbget-bin/www_bget?ko+K03083) GSK3B; glycogen synthase kinase 3 beta [EC:2.7.11.1]

cDNAv1_0.fasta.screen.Contig822

[K02214](http://www.genome.jp/dbget-bin/www_bget?ko+K02214) CDC7; cell division control protein CDC7

cDNAv1_0.fasta.screen.Contig560

[K05868](http://www.genome.jp/dbget-bin/www_bget?ko+K05868) CCNB; cyclin B

cDNAv1_0.fasta.screen.Contig378

[K06630](http://www.genome.jp/dbget-bin/www_bget?ko+K06630) YWHA; tyrosine 3-monooxygenase/tryptophan 5-monooxygenase activation protein

cDNAv1_0.fasta.screen.Contig154

[K03355](http://www.genome.jp/dbget-bin/www_bget?ko+K03355) APC8, CDC23; anaphase-promoting complex component APC8

cDNAv1_0.fasta.screen.Contig746

[K03364](http://www.genome.jp/dbget-bin/www_bget?ko+K03364) CDH1; cell division cycle 20 homolog 1, cofactor of APC complex

cDNAv1_0.fasta.screen.Contig414

[K06639](http://www.genome.jp/dbget-bin/www_bget?ko+K06639) CDC14; cell division cycle 14 [EC:3.1.3.48]

cDNAv1_0.fasta.screen.Contig28

cDNAv1_0.fasta.screen.Contig1005

[K02605](http://www.genome.jp/dbget-bin/www_bget?ko+K02605) ORC3; origin recognition complex subunit 3

cDNAv1_0.fasta.screen.Contig295

[K02607](http://www.genome.jp/dbget-bin/www_bget?ko+K02607) ORC5; origin recognition complex subunit 5

cDNAv1_0.fasta.screen.Contig699

[K02541](http://www.genome.jp/dbget-bin/www_bget?ko+K02541) MCM3; minichromosome maintenance protein 3

11h10uni.e.ab1

11h10uni.f.ab1

[K02209](http://www.genome.jp/dbget-bin/www_bget?ko+K02209) MCM5, CDC46; minichromosome maintenance protein 5 (cell division control protein 46)

cDNAv1_0.fasta.screen.Contig910

[K06647](http://www.genome.jp/dbget-bin/www_bget?ko+K06647) MBP1; transcription factor MBP1

cDNAv1_0.fasta.screen.Contig1044

[K06665](http://www.genome.jp/dbget-bin/www_bget?ko+K06665) SSN6; glucose repression mediator protein

cDNAv1_0.fasta.screen.Contig112

[K06666](http://www.genome.jp/dbget-bin/www_bget?ko+K06666) TUP1; glucose repression regulatory protein TUP1

cDNAv1_0.fasta.screen.Contig1020

[K06670](http://www.genome.jp/dbget-bin/www_bget?ko+K06670) SCC1, MCD1, RAD21; cohesin complex subunit SCC1

cDNAv1_0.fasta.screen.Contig804

[K06677](http://www.genome.jp/dbget-bin/www_bget?ko+K06677) YCS4, CNAP1; condensin complex subunit 1

cDNAv1_0.fasta.screen.Contig582

04210 Apoptosis [PATH:ko04210]

[K04725](http://www.genome.jp/dbget-bin/www_bget?ko+K04725) IAP; baculoviral IAP repeat-containing 2/3/4

cDNAv1_0.fasta.screen.Contig324

**01430 Cell Communication**

04510 Focal adhesion [PATH:ko04510]

[K06269](http://www.genome.jp/dbget-bin/www_bget?ko+K06269) PPP1C; protein phosphatase 1, catalytic subunit [EC:3.1.3.16]

cDNAv1_0.fasta.screen.Contig138

[K05692](http://www.genome.jp/dbget-bin/www_bget?ko+K05692) ACTB_G; actin, beta/gamma, cytoplasmic

cDNAv1_0.fasta.screen.Contig381

[K05699](http://www.genome.jp/dbget-bin/www_bget?ko+K05699) ACTN; actinin, alpha

cDNAv1_0.fasta.screen.Contig105

[K03083](http://www.genome.jp/dbget-bin/www_bget?ko+K03083) GSK3B; glycogen synthase kinase 3 beta [EC:2.7.11.1]

cDNAv1_0.fasta.screen.Contig822

[K04392](http://www.genome.jp/dbget-bin/www_bget?ko+K04392) RAC1; Ras-related C3 botulinum toxin substrate 1

cDNAv1_0.fasta.screen.Contig309

[K04371](http://www.genome.jp/dbget-bin/www_bget?ko+K04371) ERK1_2; extracellular signal-regulated kinase 1/2 [EC:2.7.11.24]

cDNAv1_0.fasta.screen.Contig541

[K04725](http://www.genome.jp/dbget-bin/www_bget?ko+K04725) IAP; baculoviral IAP repeat-containing 2/3/4

cDNAv1_0.fasta.screen.Contig324

04520 Adherens junction [PATH:ko04520]

[K04392](http://www.genome.jp/dbget-bin/www_bget?ko+K04392) RAC1; Ras-related C3 botulinum toxin substrate 1

cDNAv1_0.fasta.screen.Contig309

[K05699](http://www.genome.jp/dbget-bin/www_bget?ko+K05699) ACTN; actinin, alpha

cDNAv1_0.fasta.screen.Contig105

[K05692](http://www.genome.jp/dbget-bin/www_bget?ko+K05692) ACTB_G; actin, beta/gamma, cytoplasmic

cDNAv1_0.fasta.screen.Contig381

[K03097](http://www.genome.jp/dbget-bin/www_bget?ko+K03097) CSNK2A; casein kinase 2, alpha polypeptide [EC:2.7.11.1]

cDNAv1_0.fasta.screen.Contig969

[K04371](http://www.genome.jp/dbget-bin/www_bget?ko+K04371) ERK1_2; extracellular signal-regulated kinase 1/2 [EC:2.7.11.24]

cDNAv1_0.fasta.screen.Contig541

04530 Tight junction [PATH:ko04530]

[K03456](http://www.genome.jp/dbget-bin/www_bget?ko+K03456) PPP2R1; protein phosphatase 2 (formerly 2A), regulatory subunit A

cDNAv1_0.fasta.screen.Contig738

[K04354](http://www.genome.jp/dbget-bin/www_bget?ko+K04354) PPP2R2; protein phosphatase 2 (formerly 2A), regulatory subunit B

cDNAv1_0.fasta.screen.Contig205

[K03097](http://www.genome.jp/dbget-bin/www_bget?ko+K03097) CSNK2A; casein kinase 2, alpha polypeptide [EC:2.7.11.1]

cDNAv1_0.fasta.screen.Contig969

[K05692](http://www.genome.jp/dbget-bin/www_bget?ko+K05692) ACTB_G; actin, beta/gamma, cytoplasmic

cDNAv1_0.fasta.screen.Contig381

[K05699](http://www.genome.jp/dbget-bin/www_bget?ko+K05699) ACTN; actinin, alpha

cDNAv1_0.fasta.screen.Contig105

04540 Gap junction [PATH:ko04540]

[K04371](http://www.genome.jp/dbget-bin/www_bget?ko+K04371) ERK1_2; extracellular signal-regulated kinase 1/2 [EC:2.7.11.24]

cDNAv1_0.fasta.screen.Contig541

[K02218](http://www.genome.jp/dbget-bin/www_bget?ko+K02218) CSNK1, CK1; casein kinase 1 [EC:2.7.11.1]

12e09uni.t.ab1

17a05uni.t.ab1

**01460 Immune System**

04640 Hematopoietic cell lineage [PATH:ko04640]

[K01256](http://www.genome.jp/dbget-bin/www_bget?ko+K01256) E3.4.11.2, pepN; membrane alanyl aminopeptidase [EC:3.4.11.2]

cDNAv1_0.fasta.screen.Contig529

04620 Toll-like receptor signaling pathway [PATH:ko04620]

[K04392](http://www.genome.jp/dbget-bin/www_bget?ko+K04392) RAC1; Ras-related C3 botulinum toxin substrate 1

cDNAv1_0.fasta.screen.Contig309

04650 Natural killer cell mediated cytotoxicity [PATH:ko04650hsa]

[K04392](http://www.genome.jp/dbget-bin/www_bget?ko+K04392) RAC1; Ras-related C3 botulinum toxin substrate 1

cDNAv1_0.fasta.screen.Contig309

[K04371](http://www.genome.jp/dbget-bin/www_bget?ko+K04371) ERK1_2; extracellular signal-regulated kinase 1/2 [EC:2.7.11.24]

cDNAv1_0.fasta.screen.Contig541

04612 Antigen processing and presentation [PATH:ko04612]

[K03283](http://www.genome.jp/dbget-bin/www_bget?ko+K03283) TC.HSP70; heat shock protein 70, Hsp70 family [TC:1.A.33]

cDNAv1_0.fasta.screen.Contig1035

[K04079](http://www.genome.jp/dbget-bin/www_bget?ko+K04079) HTPG, HSP90; molecular chaperone HtpG (Hsp90)

cDNAv1_0.fasta.screen.Contig1039

[K08056](http://www.genome.jp/dbget-bin/www_bget?ko+K08056) PDIA3, GRP58; protein disulfide isomerase family A, member 3 [EC:5.3.4.1]

cDNAv1_0.fasta.screen.Contig724

04660 T cell receptor signaling pathway [PATH:ko04660]

04662 B cell receptor signaling pathway [PATH:ko04662]

[K04392](http://www.genome.jp/dbget-bin/www_bget?ko+K04392) RAC1; Ras-related C3 botulinum toxin substrate 1

cDNAv1_0.fasta.screen.Contig309

[K03083](http://www.genome.jp/dbget-bin/www_bget?ko+K03083) GSK3B; glycogen synthase kinase 3 beta [EC:2.7.11.1]

cDNAv1_0.fasta.screen.Contig822

04664 Fc epsilon RI signaling pathway [PATH:ko04664]

[K04392](http://www.genome.jp/dbget-bin/www_bget?ko+K04392) RAC1; Ras-related C3 botulinum toxin substrate 1

cDNAv1_0.fasta.screen.Contig309

[K04371](http://www.genome.jp/dbget-bin/www_bget?ko+K04371) ERK1_2; extracellular signal-regulated kinase 1/2 [EC:2.7.11.24]

cDNAv1_0.fasta.screen.Contig541

04670 Leukocyte transendothelial migration [PATH:ko04670]

[K05692](http://www.genome.jp/dbget-bin/www_bget?ko+K05692) ACTB_G; actin, beta/gamma, cytoplasmic

cDNAv1_0.fasta.screen.Contig381

[K04392](http://www.genome.jp/dbget-bin/www_bget?ko+K04392) RAC1; Ras-related C3 botulinum toxin substrate 1

cDNAv1_0.fasta.screen.Contig309

**01470 Nervous System**

04720 Long-term potentiation [PATH:ko04720]

[K06269](http://www.genome.jp/dbget-bin/www_bget?ko+K06269) PPP1C; protein phosphatase 1, catalytic subunit [EC:3.1.3.16]

cDNAv1_0.fasta.screen.Contig138

[K04371](http://www.genome.jp/dbget-bin/www_bget?ko+K04371) ERK1_2; extracellular signal-regulated kinase 1/2 [EC:2.7.11.24]

cDNAv1_0.fasta.screen.Contig541

04730 Long-term depression [PATH:ko04730]

[K03456](http://www.genome.jp/dbget-bin/www_bget?ko+K03456) PPP2R1; protein phosphatase 2 (formerly 2A), regulatory subunit A

cDNAv1_0.fasta.screen.Contig738

[K04354](http://www.genome.jp/dbget-bin/www_bget?ko+K04354) PPP2R2; protein phosphatase 2 (formerly 2A), regulatory subunit B

cDNAv1_0.fasta.screen.Contig205

[K04371](http://www.genome.jp/dbget-bin/www_bget?ko+K04371) ERK1_2; extracellular signal-regulated kinase 1/2 [EC:2.7.11.24]

cDNAv1_0.fasta.screen.Contig541

**01440 Development**

04320 Dorso-ventral axis formation [PATH:ko04320]

[K04371](http://www.genome.jp/dbget-bin/www_bget?ko+K04371) ERK1_2; extracellular signal-regulated kinase 1/2 [EC:2.7.11.24]

cDNAv1_0.fasta.screen.Contig541

04360 Axon guidance [PATH:ko04360]

[K04392](http://www.genome.jp/dbget-bin/www_bget?ko+K04392) RAC1; Ras-related C3 botulinum toxin substrate 1

cDNAv1_0.fasta.screen.Contig309

[K04371](http://www.genome.jp/dbget-bin/www_bget?ko+K04371) ERK1_2; extracellular signal-regulated kinase 1/2 [EC:2.7.11.24]

cDNAv1_0.fasta.screen.Contig541

[K03083](http://www.genome.jp/dbget-bin/www_bget?ko+K03083) GSK3B; glycogen synthase kinase 3 beta [EC:2.7.11.1]

cDNAv1_0.fasta.screen.Contig822

**01450 Behavior**

04710 Circadian rhythm [PATH:ko04710dme]

[K02218](http://www.genome.jp/dbget-bin/www_bget?ko+K02218) CSNK1, CK1; casein kinase 1 [EC:2.7.11.1]

12e09uni.t.ab1

17a05uni.t.ab1

[K03083](http://www.genome.jp/dbget-bin/www_bget?ko+K03083) GSK3B; glycogen synthase kinase 3 beta [EC:2.7.11.1]

cDNAv1_0.fasta.screen.Contig822

**01510 Neurodegenerative Disorders**

05010 Alzheimer's disease [PATH:ko05010]

[K04524](http://www.genome.jp/dbget-bin/www_bget?ko+K04524) APOE; apolipoprotein E

cDNAv1_0.fasta.screen.Contig132

[K03083](http://www.genome.jp/dbget-bin/www_bget?ko+K03083) GSK3B; glycogen synthase kinase 3 beta [EC:2.7.11.1]

cDNAv1_0.fasta.screen.Contig822

[K01408](http://www.genome.jp/dbget-bin/www_bget?ko+K01408) E3.4.24.56, IDE; insulysin [EC:3.4.24.56]

cDNAv1_0.fasta.screen.Contig729

05020 Parkinson's disease [PATH:ko05020]

[K04551](http://www.genome.jp/dbget-bin/www_bget?ko+K04551) UBB; ubiquitin B

cDNAv1_0.fasta.screen.Contig300

05030 Amyotrophic lateral sclerosis (ALS) [PATH:ko05030]

[K04567](http://www.genome.jp/dbget-bin/www_bget?ko+K04567) LYSU, KARS; lysyl-tRNA synthetase, class II [EC:6.1.1.6]

cDNAv1_0.fasta.screen.Contig790

cDNAv1_0.fasta.screen.Contig1047

05060 Prion disease [PATH:ko05060]

[K04077](http://www.genome.jp/dbget-bin/www_bget?ko+K04077) GROEL; chaperonin GroEL (Hsp60)

cDNAv1_0.fasta.screen.Contig1006

**01530 Metabolic Disorders**

04940 Type I diabetes mellitus [PATH:ko04940]

[K04077](http://www.genome.jp/dbget-bin/www_bget?ko+K04077) GROEL; chaperonin GroEL (Hsp60)

cDNAv1_0.fasta.screen.Contig1006

04930 Type II diabetes mellitus [PATH:ko04930]

[K04371](http://www.genome.jp/dbget-bin/www_bget?ko+K04371) ERK1_2; extracellular signal-regulated kinase 1/2 [EC:2.7.11.24]

cDNAv1_0.fasta.screen.Contig541

**Unclassified**

[K00222](http://www.genome.jp/dbget-bin/www_bget?ko+K00222) D14-sterol reductase

cDNAv1_0.fasta.screen.Contig296

[K00327](http://www.genome.jp/dbget-bin/www_bget?ko+K00327) NADPH-ferrihemoprotein reductase

12e08uni.t.ab1

[K00507](http://www.genome.jp/dbget-bin/www_bget?ko+K00507) stearoyl-CoA desaturase

17a01uni.t.ab1

17a01uni.e.ab1

[K00559](http://www.genome.jp/dbget-bin/www_bget?ko+K00559) sterol 24-C-methyltransferase

5f10uni.t.ab1

[K00653](http://www.genome.jp/dbget-bin/www_bget?ko+K00653) histone acetyltransferase

cDNAv1_0.fasta.screen.Contig644

[K00671](http://www.genome.jp/dbget-bin/www_bget?ko+K00671) glycylpeptide N-tetradecanoyltransferase

cDNAv1_0.fasta.screen.Contig708

[K00870](http://www.genome.jp/dbget-bin/www_bget?ko+K00870) protein kinase

cDNAv1_0.fasta.screen.Contig710

cDNAv1_0.fasta.screen.Contig733

cDNAv1_0.fasta.screen.Contig825

4a06uni.t.ab1

[K00908](http://www.genome.jp/dbget-bin/www_bget?ko+K00908) Ca2+/calmodulin-dependent protein kinase

cDNAv1_0.fasta.screen.Contig497

[K01072](http://www.genome.jp/dbget-bin/www_bget?ko+K01072) ubiquitin thiolesterase

cDNAv1_0.fasta.screen.Contig130

cDNAv1_0.fasta.screen.Contig552

cDNAv1_0.fasta.screen.Contig618

cDNAv1_0.fasta.screen.Contig674

[K01090](http://www.genome.jp/dbget-bin/www_bget?ko+K01090) protein phosphatase

cDNAv1_0.fasta.screen.Contig448

[K01372](http://www.genome.jp/dbget-bin/www_bget?ko+K01372) bleomycin hydrolase

cDNAv1_0.fasta.screen.Contig577

[K01381](http://www.genome.jp/dbget-bin/www_bget?ko+K01381) saccharopepsin

cDNAv1_0.fasta.screen.Contig612

[K01414](http://www.genome.jp/dbget-bin/www_bget?ko+K01414) endothelin-converting enzyme

cDNAv1_0.fasta.screen.Contig344

[K01422](http://www.genome.jp/dbget-bin/www_bget?ko+K01422) L-asparaginase

cDNAv1_0.fasta.screen.Contig816

[K01528](http://www.genome.jp/dbget-bin/www_bget?ko+K01528) dynamin GTPase

cDNAv1_0.fasta.screen.Contig382

[K01931](http://www.genome.jp/dbget-bin/www_bget?ko+K01931) phosphoribosylformylglycinamidine cyclo-ligase

cDNAv1_0.fasta.screen.Contig57

cDNAv1_0.fasta.screen.Contig240

cDNAv1_0.fasta.screen.Contig878

cDNAv1_0.fasta.screen.Contig906

[K03644](http://www.genome.jp/dbget-bin/www_bget?ko+K03644) lipoic acid synthetase

cDNAv1_0.fasta.screen.Contig682

[K03801](http://www.genome.jp/dbget-bin/www_bget?ko+K03801) lipoate-protein ligase B

9c07uni.e.ab1

[K03927](http://www.genome.jp/dbget-bin/www_bget?ko+K03927) carboxylesterase type B

cDNAv1_0.fasta.screen.Contig11

cDNAv1_0.fasta.screen.Contig1003

[K03979](http://www.genome.jp/dbget-bin/www_bget?ko+K03979) GTP-binding protein

cDNAv1_0.fasta.screen.Contig591

[K04564](http://www.genome.jp/dbget-bin/www_bget?ko+K04564) superoxide dismutase, Fe-Mn family

cDNAv1_0.fasta.screen.Contig346

[K05955](http://www.genome.jp/dbget-bin/www_bget?ko+K05955) [hydroxymethylglutaryl-CoA reductase (NADPH)] kinase

cDNAv1_0.fasta.screen.Contig376

[K06174](http://www.genome.jp/dbget-bin/www_bget?ko+K06174) ATP-binding cassette, sub-family E, member 1

cDNAv1_0.fasta.screen.Contig473

[K06207](http://www.genome.jp/dbget-bin/www_bget?ko+K06207) GTP-binding protein

cDNAv1_0.fasta.screen.Contig511

[K06867](http://www.genome.jp/dbget-bin/www_bget?ko+K06867) putative MFS transporter, AGZA family, xanthine/uracil permease

cDNAv1_0.fasta.screen.Contig211

[K06948](http://www.genome.jp/dbget-bin/www_bget?ko+K06948) GTPase EngC

cDNAv1_0.fasta.screen.Contig512

cDNAv1_0.fasta.screen.Contig769

[K06972](http://www.genome.jp/dbget-bin/www_bget?ko+K06972) aspartate dehydrogenase

cDNAv1_0.fasta.screen.Contig61

[K06999](http://www.genome.jp/dbget-bin/www_bget?ko+K06999) mannosyl-3-phosphoglycerate phosphatase

cDNAv1_0.fasta.screen.Contig569

[K07095](http://www.genome.jp/dbget-bin/www_bget?ko+K07095) transmembrane sensor

cDNAv1_0.fasta.screen.Contig794

[K08285](http://www.genome.jp/dbget-bin/www_bget?ko+K08285) cyclin-dependent kinase

cDNAv1_0.fasta.screen.Contig82

cDNAv1_0.fasta.screen.Contig100

[K08286](http://www.genome.jp/dbget-bin/www_bget?ko+K08286) protein-serine/threonine kinase

cDNAv1_0.fasta.screen.Contig349

cDNAv1_0.fasta.screen.Contig649

cDNAv1_0.fasta.screen.Contig714

cDNAv1_0.fasta.screen.Contig881

cDNAv1_0.fasta.screen.Contig976

[K08287](http://www.genome.jp/dbget-bin/www_bget?ko+K08287) dual-specificity kinase

cDNAv1_0.fasta.screen.Contig667

[K08288](http://www.genome.jp/dbget-bin/www_bget?ko+K08288) protein kinase C substrate 80K-H

cDNAv1_0.fasta.screen.Contig713

**[**[**KO list**](http://www.genome.jp/kegg-bin/kaas_main?mode=result&id=1149190105&mail=beslaven@yahoo.com)**]**

**[KO hierarchy]**

**[**[**Pathway map**](http://www.genome.jp/kegg-bin/kaas_main?mode=map&id=1149190105&mail=beslaven@yahoo.com)**]**

**[** [**Threshold change**](http://www.genome.jp/kegg-bin/kaas_main?mode=threshold&id=1149190105&mail=beslaven@yahoo.com) **]**

**[**[**Download**](http://www.genome.jp/kegg-bin/kaas_main?mode=dl&id=1149190105&mail=beslaven@yahoo.com)**]**

[**KEGG2**](http://www.genome.jp/kegg/kegg2.html)

[**KEGG**](http://www.genome.jp/kegg/)

[**GenomeNet**](http://www.genome.jp/)

[**Kanehisa Lab**](http://kanehisa.kuicr.kyoto-u.ac.jp/)
